# The impact on high‐grade serous ovarian cancer of obesity and lipid metabolism‐related gene expression patterns: the underestimated driving force affecting prognosis

**DOI:** 10.1111/jcmm.13463

**Published:** 2017-12-20

**Authors:** Mauricio A. Cuello, Sumie Kato, Francisca Liberona

**Affiliations:** ^1^ Division of Obstetrics and Gynecology School of Medicine Pontificia Universidad Católica de Chile Santiago Chile

**Keywords:** ovarian cancer, lipid metabolism, survival statistics, obesity, clusters, microarray, bioinformatics

## Abstract

To investigate whether specific obesity/metabolism‐related gene expression patterns affect the survival of patients with ovarian cancer. Clinical and genomic data of 590 samples from the high‐grade ovarian serous carcinoma (HGOSC) study of The Cancer Genome Atlas (TCGA) and 91 samples from the Australian Ovarian Cancer Study were downloaded from the International Cancer Genome Consortium (ICGC) portal. Clustering of mRNA microarray and reverse‐phase protein array (RPPA) data was performed with 83 consensus driver genes and 144 obesity and lipid metabolism‐related genes. Association between different clusters and survival was analyzed with the Kaplan–Meier method and a Cox regression. Mutually exclusive, co‐occurrence and network analyses were also carried out. Using RNA and RPPA data, it was possible to identify two subsets of HGOSCs with similar clinical characteristics and cancer driver mutation profiles (*e.g*. TP53), but with different outcome. These differences depend more on up‐regulation of specific obesity and lipid metabolism‐related genes than on the number of gene mutations or copy number alterations. It was also found that CD36 and TGF‐ß are highly up‐regulated at the protein levels in the cluster with the poorer outcome. In contrast, BSCL2 is highly up‐regulated in the cluster with better progression‐free and overall survival. Different obesity/metabolism‐related gene expression patterns constitute a risk factor for prognosis independent of the therapy results in the Cox regression. Prognoses were conditioned by the differential expression of obesity and lipid metabolism‐related genes in HGOSCs with similar cancer driver mutation profiles, independent of the initial therapeutic response.

## Introduction

Epidemiological evidence now points to the negative impact of obesity on ovarian carcinogenesis 1, 2. The low‐grade chronic inflammatory state characteristic of this condition not only acts as an inducing factor but can also control cancer cell behaviour and facilitate its adaptive evolution through the clinical course of the disease 3, 4. Our group and other researchers have recently provided evidence that overweight and obese ovarian cancer patients have worse outcome than leaner counterparts 5, 6. These more unfavourable results cannot be explained by differences in the completion rates of any standardized treatment (*e.g*. primary debulking *versus* neoadjuvant chemotherapy) 6, 7, 8, nor by a higher incidence of adverse effects or co‐morbidities 9, 10. Despite the facts, many clinicians continue to argue that obesity does not constitute a risk factor and do not include it among the key components that must be modified after completing treatment to improve survival 4, 11. The most important argument against considering obesity as a risk factor is that patients with similar clinical profiles and responses to treatment, regardless of their body mass index, have similar results 12. Those in favour of including obesity contend that obese and ill women represent the most severe end of the spectrum in the clinical course of a metabolic disease. In this context, there is a spectrum of conditions that include lean but metabolically unhealthy women and metabolically healthy but obese women. Both conditions are associated with an earlier onset of metabolic diseases or cardiovascular diseases 13.

Genetic and transcriptomic profiling of ovarian cancer samples allows for identifying at least four cancer subtypes, but without being able to relate them to a clinical course or prognosis. The analysis of The Cancer Genome Atlas (TCGA) makes it obvious that the mutational spectrum of ovarian cancer is limited, with most genetic events occurring at the level of copy number variation 14. Additionally, the genetic profile of the primary tumour is different from site‐specific metastatic foci, reflecting tumour heterogeneity and the influence of the tumour microenvironment. To date, 83 driver genes have been identified and are considered significant for ovarian carcinogenesis and disease progression 15. Using different combinations, several authors have proposed prognosis signatures, none of them with obvious translation to the clinical setting. Intriguingly, tumours harbouring similar mutations or driver gene expression patterns do not behave similarly, some recurring earlier and others later. Some remain sensitive, while others are completely resistant.

As has been performed with cancer, researchers have characterized the genetic profile of obesity and metabolic disease. A list of genes associated with these morbidities have been defined by consensus and serve as useful markers in identifying patients at risk, independent of their current body mass index or phenotype 16, 17. More importantly, some expression patterns have identified women who behave as morbidly obese, although they are thin, and in turn, metabolically healthy obese women who behave similarly to thin and healthy counterparts. Based on this information, we decided to assess whether such obesity and abnormal metabolism‐related gene expression patterns affect the survival of ovarian cancer patients with the same driver mutation profile.

## Methods

### Selection of driver and obesity/metabolism‐related genes

The list of 83 mutational cancer driver genes was retrieved from the catalogue of driver mutations (2016.5) available at the website www.intogen.org created by the Biomedical Genomics Group (Barcelona, Spain). The list resulted from the mutational analysis of 316 samples of HGSOC as part of the TCGA study and represents the most recurrently mutated cancer driver genes in this histological subtype 14. The obesity‐ and abnormal metabolism‐related gene list was built up by a curation approach using different sources including the most recent report of the human obesity gene map 17. A total of 144 genes were included on the list.

### Download and analysis of TCGA and Australian Ovarian Cancer Study data for high‐grade serous ovarian cancer

The latest data available for two cohorts, the TCGA (US‐OVCA) and Australian Ovarian Cancer Study (AUSY‐OVCA), were downloaded in March 2017 from the open‐access Genomic Data Commons (portal.gdc.cancer.gov) and the International Cancer Genome Consortium (ICGC) data portals (dcc.icgc.org), respectively. We also obtained controlled data access (DACO‐1040139) from ICGC that allowed us to download the raw data for a deeper and unbiased analysis. Only HGSOC cases were included in the analyses. Using cBioPortal tools 18, we first analyzed US‐OVCA mutation data from whole‐exome sequencing, putative copy number alterations (from 590 cases determined using GISTIC 2.0), mRNA expression *Z*‐scores (using a threshold ±2.0, Agilent microarray), protein expression *Z*‐scores (using a ±2.0 threshold measured by either a reverse‐phase protein array [RPPA] or mass spectrometry) and all available clinical data and uploaded later to GenomeSpace portal (gsui.genomespace.org) for *in‐silico* multistep analysis using pipelines available in the gene pattern platform version 3.9.9 (Broad Institute, MIT,USA) 19. AUSY‐OVCA raw mRNA expression and clinical data (91 cases) were first analyzed using ICGC tools (*e.g*. cohort comparison and oncogrid) and then uploaded to the genome space platform for *in‐silico* analysis with the gene pattern tools. The data sets from both cohorts were pre‐processed and clustered by driver and obesity‐related genes using non‐negative matrix factorization (NMF) 20. The cophenetic correlation coefficient and the average silhouette width calculation were used to determine the most robust clusters. Differential expression analyses were carried out using the comparative marker selection module (genepattern) to calculate the significant differences in gene expression between classes. Array‐based data, either for mRNA or protein expression levels, were displayed in a heat map format using the HeatMapViewer v14 module (genepattern) to facilitate pattern identification.

The most robust NMF consensus clustering of copy number variations (579 samples, 73 copy number focal peak regions, https://doi.org/10.7908/c1xs5ttz) mRNA (569 samples, using the 1500 most variable genes, https://doi.org/10.7908/c1dn44h7), methylation (582 samples, using the 2146 most variable genes, https://doi.org/10.7908/c1bv7g24), and RPPA data (405 samples, using the 208 most variable proteins, https://doi.org/10.7908/c1p55mzw) were retrieved from the Firehose portal (gdac.broadinstitute.org, Broad Institute, MIT, USA) to externally cross‐validate the relevance and robustness of obesity/metabolism‐related gene clustering.

### Survival analysis

Kaplan–Meier survival analysis was performed with JMP version 13 software (SAS Institute Inc., Cary, NC, USA); Significance was estimated with the log rank test. The Cox proportional hazards regression model and Wald tests were used to evaluate the relationship between survival time and different clinical and molecular variables. Primary therapy result was defined according RECIST criteria (complete remission/response, partial remission/response, stable disease, progressive) after completion of the originally planned treatment (either primary debulking surgery followed by chemotherapy or neoadjuvant chemotherapy plus interval debulking surgery). Overall survival (OS) was defined in the US‐OVCA cohort as the interval from the date of initial surgical resection to the date of last known contact or death. Progression‐free survival (PFS) was defined for the US‐OVCA cohort as the interval from the date of initial surgical resection to the date of progression, recurrence, or last known contact if the patient was alive and had not recurred. Follow‐up for the AUSY‐OVCA began after treatment was completed.

### Gene interactions, mutually exclusive, co‐occurrence and network analysis

Gene and biological interaction analyses were carried out using the open‐access cBioPortal tools, following same methodology as described by Ciriello *et al*. 21 and visualized using Cytoscape 3.5.1.

## Results

### Patients expressing a subset of obesity/metabolism‐related genes have poorer survival than those exhibiting the mirror pattern

**Figure 1:**
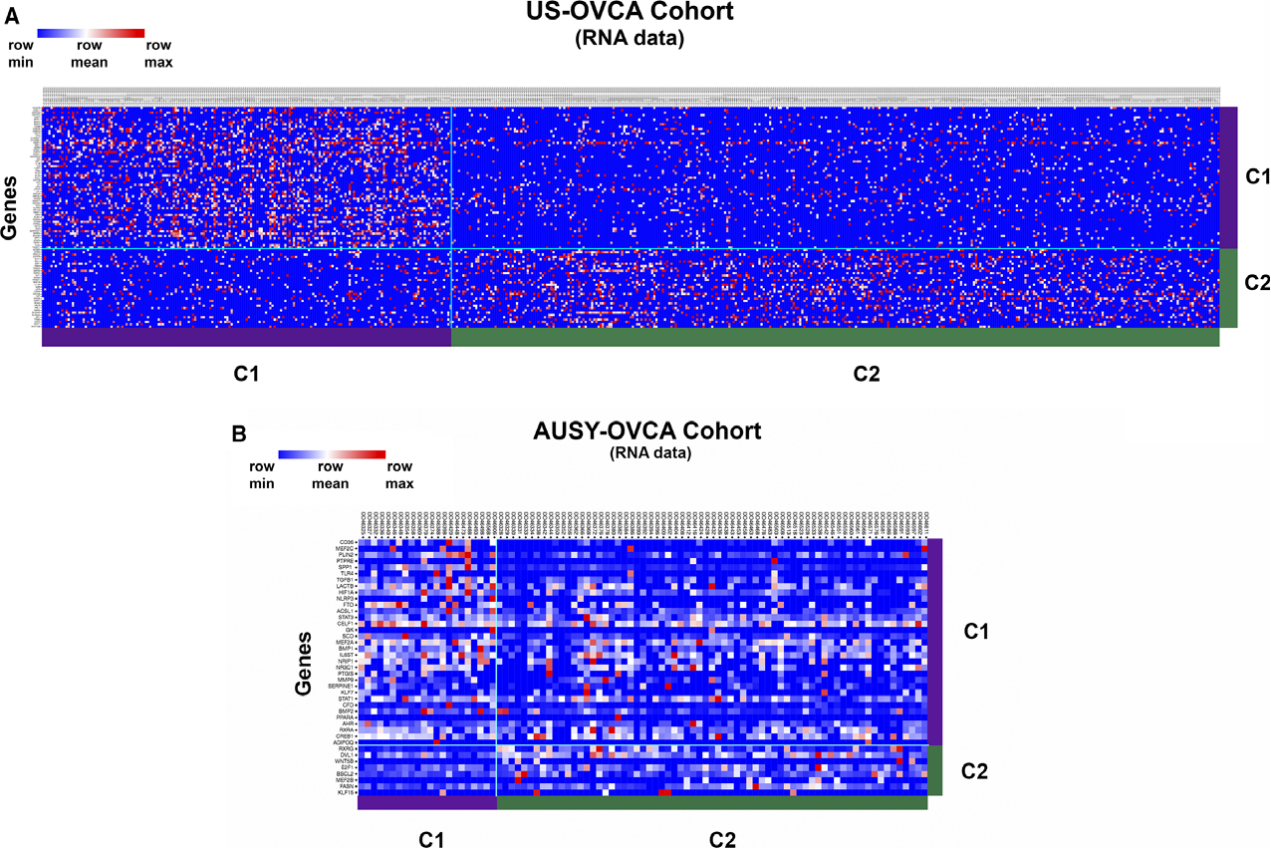
Heat map of NMF clustering of RNA expression microarray data of US‐OVCA (TCGA data set [**A**]) and AUSY‐OVCA (Australian Ovarian Cancer Study [**B**]) cohorts based on obesity and lipid metabolism‐related gene expression in primary tumour samples.

**Figure 2:**
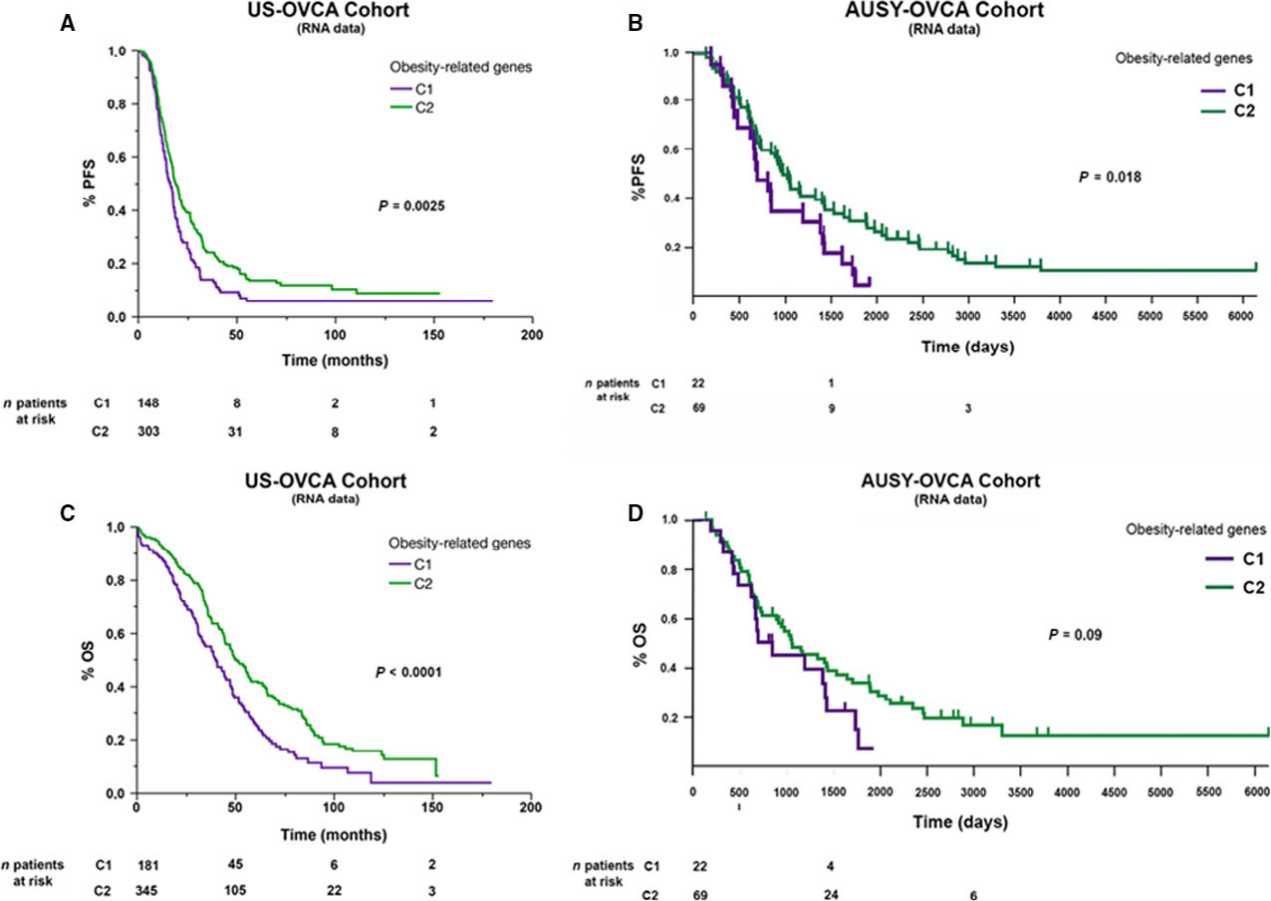
Comparative analysis of progression‐free survival (PFS) and overall survival (OS) of two clusters (named as C1 and C2) obtained after NMF clustering of RNA expression microarray data of obesity and lipid metabolism‐related genes of HGOSCs from the two cohorts.

Using NMF analysis of RNA microarray data, we robustly segregated the US‐OVCA (590 cases) and AUSY‐OVCA (91 cases) cohorts into two clusters based on our list of 144 selected obesity/metabolism‐related genes (cophenetic coef. 0.9947 for TCGA data and 0.97 for AUSY data, respectively). The list of 144 genes and details of their biological functions are provided in Table S1. Our clustering process was externally validated by comparing it to published examples of robust clustering accessible through the Firehose portal. Statistical analyses are provided in Table S2. Ninety‐eight genes of this list were expressed significantly differently between clusters in the US‐OVCA cohort and 34 genes in the AUSY‐OVCA (feature *P *<* *0.05) (detailed information is provided in Table S3). Among the genes consistently and more significantly (*P *<* *0.001) up‐regulated in cluster 1 in both cohorts were CD36, TGF‐ß, LACTB, TLR4, MEF2C and PTPRE. On the other hand, genes consistently up‐regulated in cluster 2 were RXRG, DVL1, MEF2B, BSCL2, and E2F1. Differences in gene expression patterns between clusters are presented as heat maps in Figure 1A and B. As shown in Figure 2, PFS and OS were significantly shorter in cluster 1 than in cluster 2 in the US‐OVCA cohort (PFS *P*‐value < 0.003 and OS *P*‐value < 0.0001). Median PFS and OS for clusters 1 and 2 were 16.1 and 19.2 months and 39.9 and 50.3 months, respectively. Similarly, PFS was significantly shorter in cluster 1 than in cluster 2 in AUSY‐OVCA cohort (PFS *P*‐value = 0.018), and OS showed a tendency towards longer survival among members of cluster 2 (OS *P*‐value = 0.09). The median PFS and OS for cluster 1 compared with cluster 2 were 3.6 *versus* 6 months and 28.1 *versus* 35 months, respectively.

**Figure 3:**
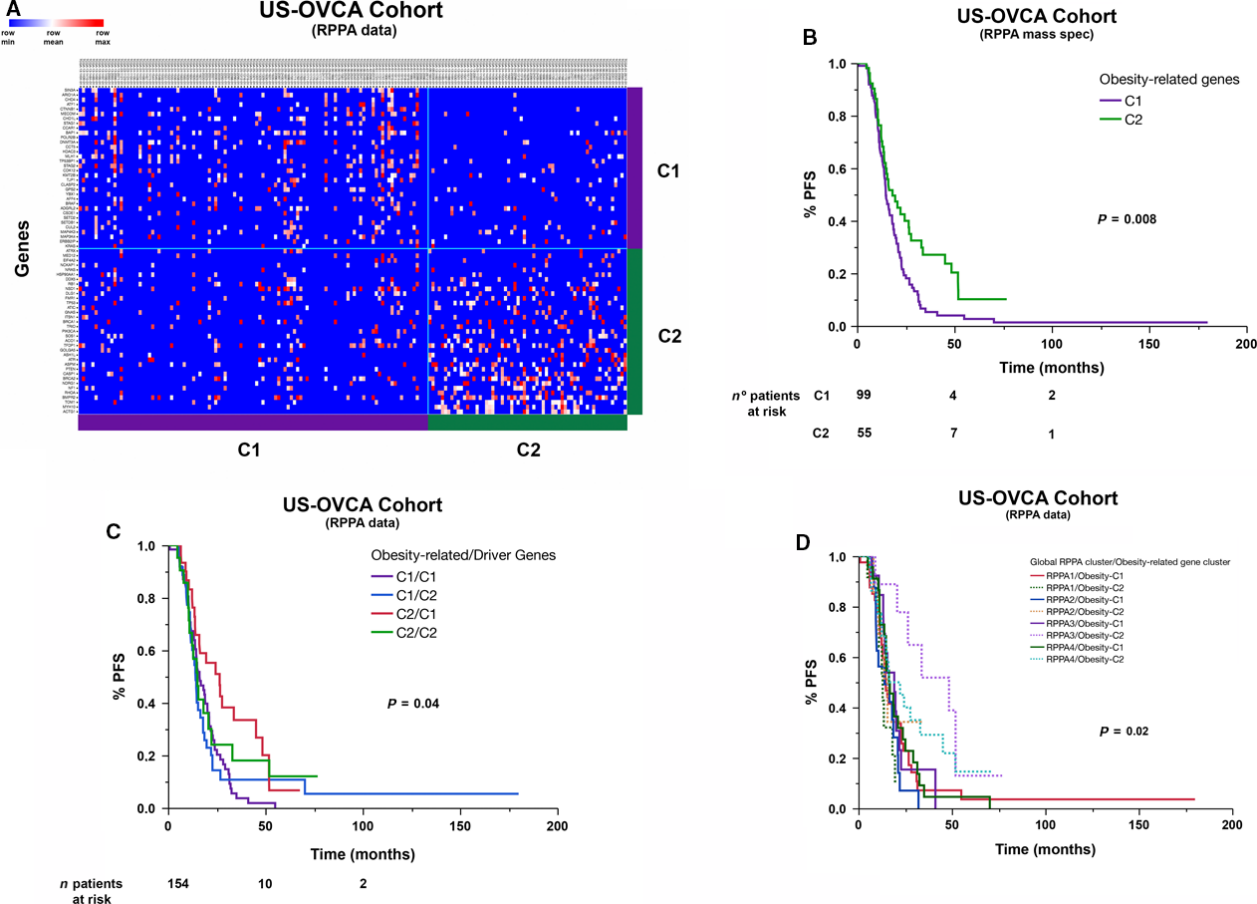
Heat map view and survival analyses of two clusters obtained after NMF analysis using RPPA expression (microarray and mass spectrometry) data of obesity and lipid metabolism‐related and cancer driver genes. (**A**) Heat map view of clusters obtained after NMF analysis of RPPA data of obesity and lipid metabolism‐related genes. (**B**) Comparative analysis of PFS between these two clusters, including only TP53 mutant samples. (**C**) Stratification of PFS curves after overlapping clusters obtained by NMF analysis of obesity/metabolism‐related gene RPPA expression on top of clusters obtained by NMF analysis of cancer driver genes in HGOSCs. (**D**) Stratification of PFS curves after overlapping clusters obtained by NMF analysis of obesity/metabolism‐related gene RPPA expression on top of clusters obtained by best NMF clustering of global RPPA expression data in HGOSCs.

Given that changes in RNA expression levels do not necessarily correlate with changes in protein levels, we sought to determine whether RNA expression patterns match protein expression patterns, given the relevance of the latter for diagnosis. Using NMF analysis of RPPA data of 174 matched RNA samples, we confirmed the two clusters already identified (cophenetic coef. 0.9867, see Fig. 3A). At the protein level, there were 28 genes expressed differently, with statistical significance (all with *P*‐value < 0.01). Among those considered significant at the RNA expression level, CD36 and TGF‐ß were also up‐regulated at protein levels in cluster 1 and BSCL2 in cluster 2, respectively. As observed with RNA data, different outcome in terms of PFS and OS were also found between clusters based on the expression patterns. As shown in Figure 3B, PFS was significantly shorter in cluster 1 (PFS *P*‐value < 0.007). The median PFS for cluster 1 compared with cluster 2 was 14.8 *versus* 18.2 months, respectively.

### HGOSCs harbouring cluster 1 obesity/metabolism‐related gene expression pattern have poorer survival rates than cluster 2, despite sharing the same cancer driver gene profile

To determine the impact of different obesity/metabolism‐related gene expression patterns in the prognosis of ovarian cancer prognosis, we carried out a second NMF clustering analysis of RPPA data, this time using the most variable of the 83 cancer driver genes for HGOSC (seeTable S4). Our hypothesis was that the differential expression of cancer driver genes can serve to identify clusters associated with different outcome. More importantly, such clusters combined with those identified by differential expression of obesity‐related genes cancelled out the effect observed with each other. We found 64 genes that are expressed with statistically significant differences sufficient to identify two clusters (*P*‐value < 0.01). Despite obtaining robust clustering, no differences were found in terms of PFS or OS between the two clusters (see Fig. S1). Unexpectedly, combining the two clustering did not cancel out the effect. On the contrary, it resulted in four clusters with different PFS. More importantly, the clusters harbouring a similar gene up‐regulation pattern, as seen with class 1 obesity/metabolism‐related gene clustering, showed shorter PFS (see Fig. 3C). To further confirm this finding, we repeated the exercise adding our obesity/metabolism‐related gene expression patterns on top of the most robust RPPA clustering so far published. This clustering included the 208 most variable proteins in HGOSC (see Tables S5 and S6). Re‐grouping the clusters with the combination of patterns did not eliminate or dilute the association of obesity‐related gene expression patterns with ovarian cancer prognosis. In fact, RPPA‐derived clusters with up‐regulation obesity/metabolism‐related genes, as seen in cluster 1, tended to have significantly shorter PFS than those with a cluster 2 obesity/metabolism‐related gene expression pattern (see Fig. 3D).

**Figure 4:**
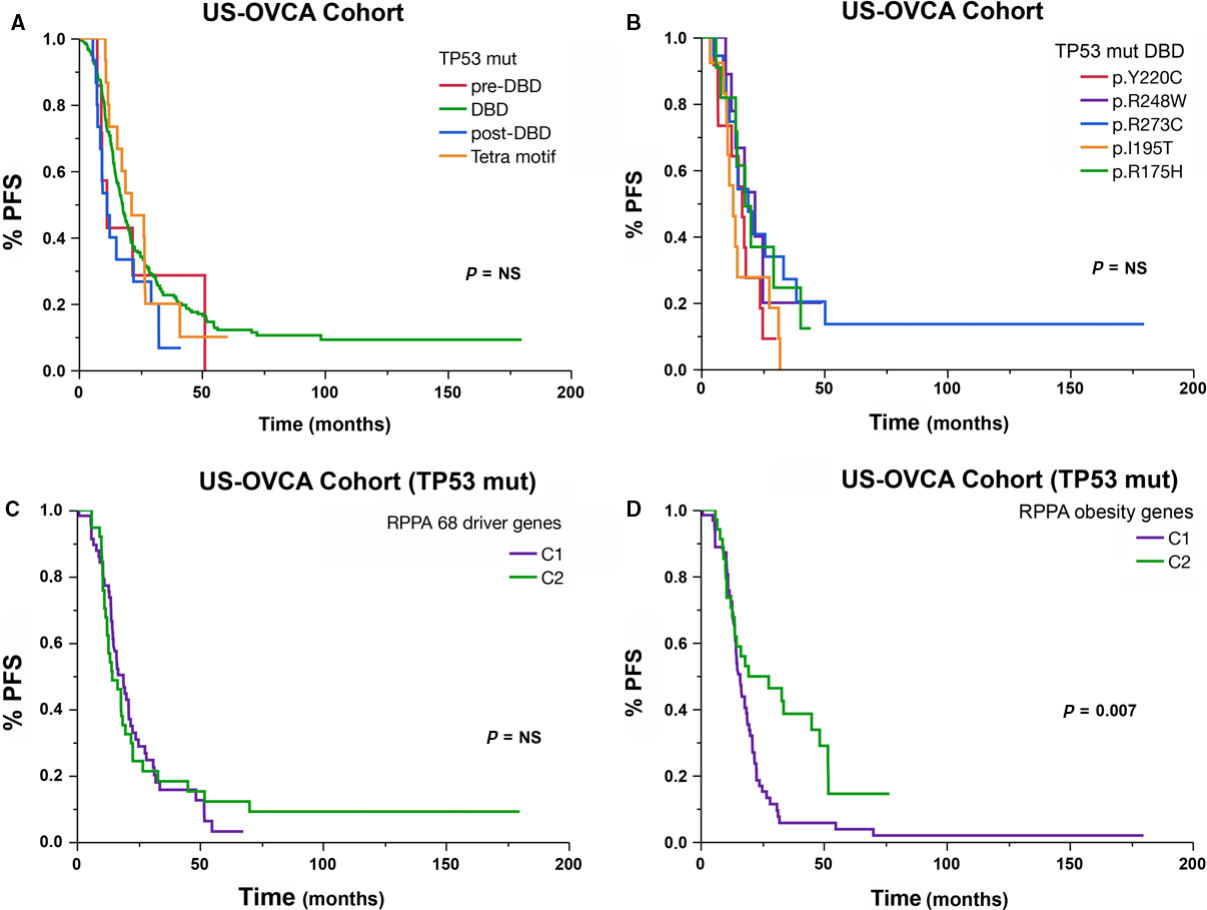
Comparative analysis of PFS curves after NMF clustering using RNA expression microarray data of obesity and lipid metabolism genes in HGOSCs carrying different TP53 mutations.

**Figure 5:**
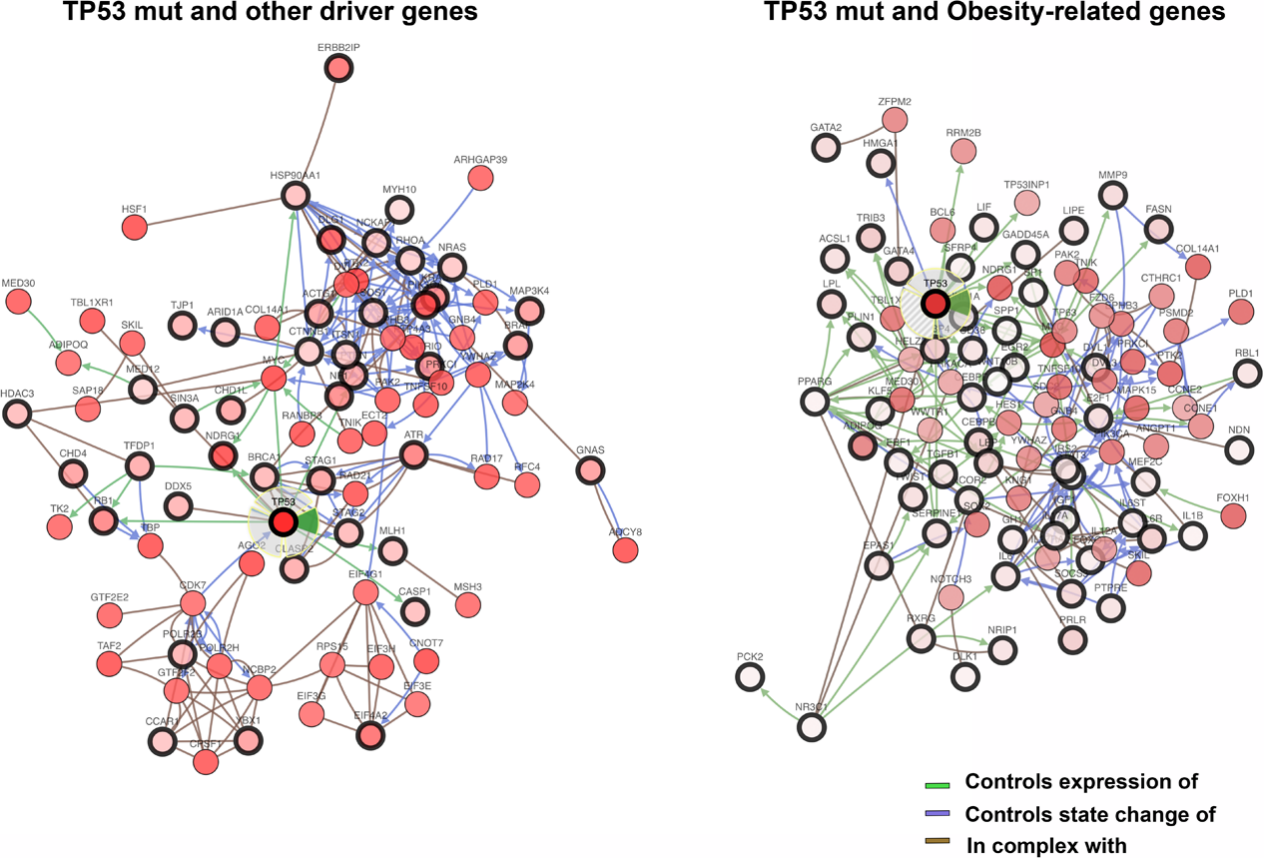
Graphical representation of network analysis and main biological interactions of cancer driver and obesity/metabolism‐related genes in relation to TP53 mutant in HGOSCs. Circles with wider black lines indicate the query genes, and those delimited by thin black lines highlight the more altered neighbour genes.

All the cases in our RPPA clustering analysis were carriers of TP53 mutations, one of the key cancer driver genes for HGOSC. As Figure 4 shows, no differences were found in terms of PFS and OS among different TP53 mutations. We did a network analysis to assess and compare the strengths of the biological interactions of TP53 mutant gene to those of other cancer driver genes and the obesity/metabolism‐related genes included in our analyses. We intended to weigh the relevance or impact of biological interactions determined by either cancer driver or obesity/metabolism‐related genes on cancer behaviour and therapeutic outcome. On the one hand, the network shown in Figure 5A contains 118 nodes, including our 68 cancer driver query genes and the 50 most frequently altered neighbour genes (of a total of 3138). On the other hand, the network in Figure 5B contains 146 nodes, including our 96 obesity/metabolism‐related query genes and the 50 most frequently altered neighbour genes (of a total of 2267). These findings suggest a higher and more closely matched number of TP53 mutant gene and obesity/metabolism‐related genes, with denser interactions between them than between TP53 mutant cells and other cancer driver genes. Many of these interactions are related to adaptive metabolism, acquisition of drug resistance, metastatic and pro‐survival signals. The co‐occurrence and mutually exclusive analyses are summarized in Table S7.

### The up‐regulation of obesity/metabolism‐related genes has an independent negative impact on HGOSC prognosis: a reflection of environmental influences on cancer behaviour

Table 1 summarizes the genetic and clinical characteristics of the HGOSCs of the two clusters. No difference was observed in terms of ethnicity or race between clusters. As shown, patients in cluster 1 share similar numbers of mutations but a significantly lower average number of gene copy alterations. Cluster 1 had a higher percentage of patients in stage IV, a lower number of cases achieving microscopic disease after surgical debulking and a lower number of complete responses after the completion of treatment (primary therapy result). To confirm the independent effect of obesity/metabolism‐related gene expression patterns on outcome, a Cox model was built that included obesity/metabolism‐related gene RPPA clustering, primary therapy result (complete response *versus* others), and stratified age. In the absence of clinical data related to BMI/obesity in the TCGA cohort, age at diagnosis was included in the model as indirect assessment of obesity and metabolic disorders, as both conditions significantly increase with ageing and particularly after menopause. We also assessed ethnicity and race, both were excluded from the model because of *P*‐value ≥ 0.1. According to the Cox model, patients exhibiting different obesity/metabolism‐related gene expression patterns had different outcome independent of their primary therapy results or their age (*P* =* *0.04). As expected, the primary therapy results constituted the most significant factor influencing the complete model (*P*‐value = 0.00002). In contrast, age did not constitute an independent factor.

**Table 1:** Clinical and molecular characteristics of two clusters obtained after NMF analysis using obesity and lipid metabolism gene expression data from TCGA

	NMF Clustering by Obesity‐related Genes		*P*‐value
	Cluster 1	Cluster 2	
*n*	186	352	
Age (years)	58.8 ± 11.2	59.9 ± 11.8	NS
Stage			0.02
I	2 (1.1%)	14 (4%)	NS
II	7 (3.8%)	19 (5.4%)	
III	137 (73.7%)	275 (78.1%)	0.03
IV	36 (19.4%)	41 (11.57%)	
NA	4 (2.2%)	3 (0.9%)	
Mutation count	49.5 ± 2.5	48.1 ± 2	NS
Copy number alterations	0.49 ± 0.18	0.59 ± 0.18	<0.0001
Histological grade			
G1	0 (0%)	4 (1.2%)	NS
G2	26 (14%)	41 (11.8%)	
G3	156 (83.9%)	296 (84.8%)	
Gx	3 (1.6%)	5 (1.4%)	
NA	1 (0.5%)	3 (0.9%)	
Primary diagnosis			
Tumour resection	143 (76.9%)	287 (81.5%)	NS
Fine needle aspiration biopsy	4 (2.2%)	6 (1.7%)	
Cytology (*e.g*. Peritoneal or pleural fluid)	27 (14.5%)	44 (12.5%)	
Incisional Biopsy	5 (2.7%)	7 (2%)	
Excisional Biopsy	4 (2.2%)	1 (0.3%)	
Other methods	0 (0%)	1 (0.3%)	
NA	3 (1.6%)	6 (1.7%)	
Residual disease after surgery			
No macroscopic disease	16 (8.6%)	87 (24.7%)	<0.0001
1–10 mm	96 (51.6%)	134 (38.1%)	
11–20 mm	14 (7.5%)	19 (5.4%)	
>20 mm	38 (20.4%)	61 (17.3%)	
NA	22 (11.8%)	51 (14.5%)	
Primary optimal debulking (<1 cm)	112 (60.2%)	221 (62.8%)	NS
Chemotherapy (Chemo)			
Adjuvant	115 (61.8%)	235 (73.9%)	NS
Progression	27 (14.5%)	30 (9.4%)	
Recurrence	22 (11.8%)	47 (14.8%)	
Other	2 (1.1%)	6 (1.9%)	
NA	20 (10.8%)	35 (10%)	
≥three rescue chemo lines	17 (10.2%)	30 (9.5%)	NS
Primary therapy result			
Complete remission/response	93 (50%)	203 (57.8%)	0.002
Partial remission/response	33 (17.7%)	27 (7.7%)	
Stable disease	11 (5.9%)	16 (4.6%)	
Progressive disease	16 (8.6%)	19 (5.4%)	
NA	33 (17.7%)	86 (24.5%)	
Disease Status			
Disease‐Free	32 (17.2%)	96 (27.3%)	0.008
Recurred/progressed	116 (62.4%)	210 (59.7%)	
NA	38 (20.4%)	46 (13.1%)	

## Discussion

In the present study, using two HGOSC cohorts, we demonstrated that there are at least two subsets of patients with similar clinical characteristics and cancer driver mutation profiles, but having different outcome based on expression patterns of genes related to obesity and lipid metabolism.

An additional finding of our analyses is the fact that the cancers included in the clusters share common patterns of mutated genes, including characteristic genes such as TP53 or BRCA. They also have similar numbers of mutations and only vary in the number of copies for such genes. By combining the clusters that are obtained with the list of cancer driver genes and the list of genes related to obesity and lipid metabolism, we obtained four clusters with different prognoses. Those with similar patterns of cancer driver gene expression have poorer prognoses when they express higher levels of a subset of genes related to obesity or lipid metabolism (*e.g*. CD36 or TGF‐ß). Interestingly, those are the same clusters that exhibit lower averages of gene copy number variations. These findings lead us to propose that once the chain of mutations necessary for a cancer to develop has taken place, its subsequent behaviour and evolution depends more on environmental influences to which the cancer cells must adapt. Additionally, it seems that fewer copy number alterations of cancer driver genes are required to modulate HGOSC behaviour when obesity and lipid metabolism alterations are in place. Obesity causes epigenetic changes that usually do not have a hereditary character, but that condition future risks and morbidities in the subjects that are exposed to it from an early age or for a prolonged period 22, 23. Another environmental influence that conditions gene expression and determines evolutionary adaptations in cancer cells is recurrent exposure to chemotherapy. In fact, the evidence supporting the acquisition of cross‐resistance to chemotherapy agents with different mechanisms of action is further substantiated 24; a condition that does not depend on mutations but rather on the differential expression of genes linked to resistance, including TGF‐ß 25, 26.

*In vitro* and *in vivo* studies support the adaptive capacity of cancer cells depending on the location and environmental conditions where they are exposed. In fact, ovarian cancer cells in response to such scenarios undergo mesenchymal‐epithelial transition (EMT), which determines not only changes in the expression of adhesion molecules, but also metabolic adaptations that allow them to survive in conditions of hypoxia or nutrient shortage 27, 28. This explains, for example, the presence of cellular aggregates floating in ascites, a defence and survival mechanism against adverse environmental conditions 29. There is now evidence that key mutations for ovarian carcinogenesis, such as in TP53, are related not only to cell proliferation or survival, but also to metabolic adaptations, particularly related to lipid metabolism. In fact, a hotspot and oncogenic mutation at the death‐binding domain (DBD) of TP53, at R273H residue, is associated with a mevalonate signature that results in a greater metastatic capacity in a syngeneic mouse model 30. Herein, we also analyzed the cluster allocation of cases harbouring this mutation and others similar at the DBD (*e.g*. R175H, Y220C, I195T). Interestingly, most of them (95%) allocated in the same cluster, with similar expression patterns of lipid metabolism‐related genes with no difference in PFS and OS.

We identified CD36 and BSCL2 as two significant genes defining cluster allocation and different outcome. CD36 plays a role in the regulation of angiogenesis and in fatty acid uptake by cancer cells promoting cell migration and proliferation 31, 32. Ovarian cancer cells commonly metastasize to adipose tissue (*e.g*. omentum). A key feature of peritumoural adipocytes is their loss of lipid content observed both *in vitro* and in human tumours. The free fatty acids (FFAs) released by adipocytes after lipolysis induced by tumour secretions are transferred and stored in tumour cells as triglycerides in lipid droplets, a process dependent on CD36 expression levels 33. FFAs can be released over time from lipid droplets through an adipose triglyceride lipase‐dependent (ATGL‐dependent) lipolitic pathway. The released FFAs are then used for fatty acid β‐oxidation (FAO), an active process in cancer cells, but not in normal epithelial cells, and regulated by co‐culture with adipocytes. However, in co‐cultivated cells, FAO is uncoupled from ATP production, leading to AMPK/acetyl‐CoA carboxylase activation, a circle that maintains this state of metabolic remodelling 33. Recently, it has been shown that higher expression of CD36/fatty acid translocase and elevated free fatty acid (FFA) levels is associated with hepatocellular progression *via* induction of EMT. Although obesity is manifested as elevated FFA levels, the degree of EMT was not associated with the body mass index of the patients with HCC, highlighting the specific roles of CD36 and FFA uptake 34. In contrast to CD36, BSCL2 encodes for multipass transmembrane protein seipin, a regulator of lipid catabolism essential for adipocyte differentiation. BSCL2 may also play a tissue‐autonomous role in controlling lipid storage in adipocytes and in preventing ectopic lipid droplet formation in non‐adipose tissue, among others in cancer cells 35.

Originally, we argued and provided molecular and clinical evidence to support that obese and overweight women affected by HGOSC have poorer outcome 6. A key determinant is the higher expression of leptin and its OB‐Rb receptor, which in turn prompts EMT and cell plasticity, leading to drug resistance and metastasis. These findings were externally validated by assessing the TCGA cohort, in which patients exhibiting higher levels of leptin at mRNA levels also had shorter PFS and OS. Herein, leptin was also included in the list of genes evaluated and remains as a significant gene for cluster allocation using RNA microarray data. Unfortunately, insufficient or no information was found to confirm its role when using RPPA mass spectrometry or microarray data at TCGA. We still argue that obesity is a risk factor to be considered in the comprehensive treatment of HGOSC—a matter that many of us, as clinicians, forget to consider, particularly during chemotherapy and follow‐up. In fact, we recently realized that the HGOSCs with poorer outcome in our own cohort were those patients with abnormal BMI or significantly higher weight during those stages. However, we also identified patients with normal or below normal BMI and with adverse outcome. These patients exhibited haematologic and biochemical parameters suggestive of sustained inflammation or abnormal levels of cholesterol (manuscript in preparation). Regrettably, both available collaborative data sets (TCGA and Australian Ovarian Cancer Study) have not included in clinical data collection information on BMI/obesity or diabetes to validate our current findings.

Based on our mutually exclusive, co‐occurrence and networking analysis, we show that obesity and lipid metabolism genes interact closely and densely with cancer driver genes, particularly TP53 mutants, determining the final biological effects and outcome. Reinartz *et al*. recently constructed a network of autocrine and paracrine signalling pathways comprising 358 common and 58 patient‐specific signalling mediators and their receptors using different transcriptome‐derived data sets 36. Through the meta‐analysis of the RNA microarray data of 1018 patients, they established clinical correlations for several components with different cellular origins and targets. As we demonstrated with our cohorts, they show obvious associations between earlier recurrences and the expression of STAT‐3‐inducing cytokines, TGFβ/BMP‐triggered pathways, secretory macrophage‐derived phospholipase PLA2 G7, its product arachidonic acid (AA) and signalling pathways controlled by the AA metabolites PGE2, PGI2, and LTB4. More recently, Ke Ch *et al*. did a metabolomic analysis to assess the metabolic changes in response to advanced HGOSC, surgery and recurrence 37. Among their findings, primary HGSOCs were characterized by abnormal lipid metabolism and energy disorders. More importantly, recurrent cases showed increased amino acid and lipid metabolism compared with the same cases at the time of diagnosis.

Taken together, it seems that metabolic adaptations are critical for cancer cells in terms of adapting to environmental conditions and evolving. Particularly, lipid metabolism seems to play a main role for ovarian cancer progression 38. From a clinical perspective, those women with these metabolic disorders (obese or not) present themselves, at the moment of diagnosis, with a disease whose distribution and characteristics make it more difficult to achieve the optimal cytoreduction, a handicap, that if such disorder is maintained after completing its treatment, by certainty, it will negatively impact in the final outcome. No doubt, these proposals deserve further research and consideration to provide proper, comprehensive and potentially more successful cancer treatment. We strongly believe that the future clinical data collections might include these parameters, before, through and after treatment, and during the follow‐up to confirm or discard the relevance of metabolic abnormalities (*e.g*. obesity or lipid metabolism disorders) in ovarian cancer biology and prognosis. In addition, our efforts must not be limited to earlier and correct diagnosis, optimal debulking and offering better chemotherapy. We should also consider patient characteristics, such as weight, adiposity, use of other medications (*e.g*. statins, metformin), diet and life style, all conditions that influence global gene expression and biological interactions among the different cell components present in the tumour microenvironment 39, 40, 41.

## Conflict of interest

The authors declare that there is no conflict of interest.

## Supporting information


**Fig. S1** Comparison of progression‐free (PFS) and overall (OS) survival between the two clusters obtained after NMF analysis using 83 cancer driver genes for HGOSC.

**Table S1** List of obesity and lipid metabolism‐related genes and their main functions.

**Table S2** Statistical analysis of differences in the distribution of robust RNAseq, copy number alteration, methylation and RPPA subgroups between the two clusters obtained by NMF analysis using obesity and lipid metabolism‐related gene expression.

**Table S3** Significant Obesity and lipid metabolism‐related genes for clustering

**Table S4** List of cancer driver genes and their functions

**Table S5** The list of driver and obesity/lipid metabolism‐related genes included in NMF clustering using RPPA data from TCGA.

**Table S6** The list of most variable genes in HGSOC according to RPPA from TCGA dataset.

**Table S7** Summary of co‐occurrence and mutually exclusive analyses carried out among TP53 mutant genes and the list of obesity and lipid metabolism‐related genes significant for NMF clustering of the HGOSC cohorts.
