# Supplementary figures and images for: The impact on high‐grade serous ovarian cancer of obesity and lipid metabolism‐related gene expression patterns: the underestimated driving force affecting prognosis
Source: J Cell Mol Med. 2017 Dec 20;22(3):1805–15. doi: 10.1111/jcmm.13463 (PMC5824367; doi:10.1111/jcmm.13463)

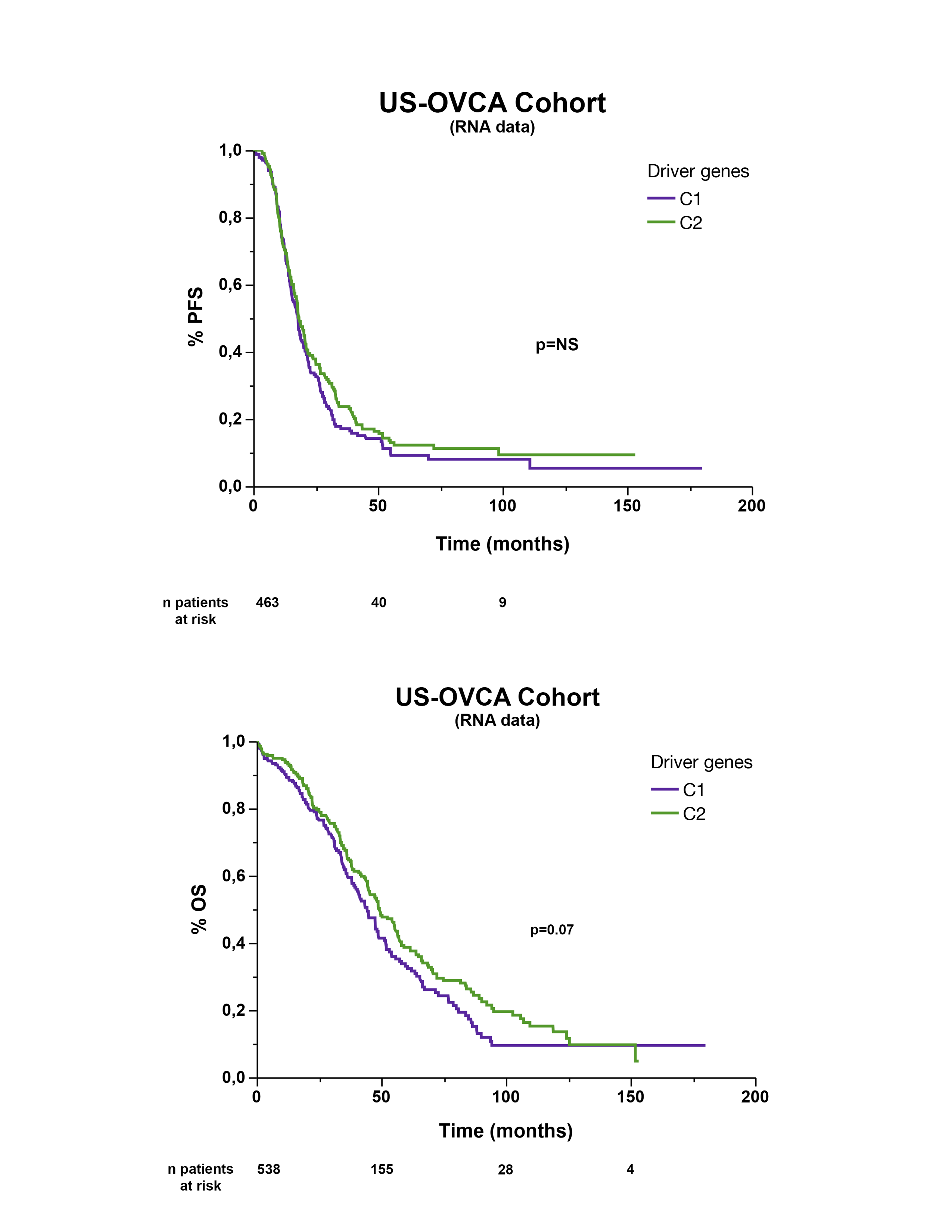

Supplement: Supplementary file 1 — Fig. S1 Comparison of progression‐free (PFS) and overall (OS) survival between the two clusters obtained after NMF analysis using 83 cancer driver genes for HGOSC. [file JCMM-22-1805-s001.tif]
