# Supplementary material for: The impact on high‐grade serous ovarian cancer of obesity and lipid metabolism‐related gene expression patterns: the underestimated driving force affecting prognosis
Source: J Cell Mol Med. 2017 Dec 20;22(3):1805–15. doi: 10.1111/jcmm.13463 (PMC5824367; doi:10.1111/jcmm.13463)
Supplement: Supplementary file 2 — Table S1 List of obesity and lipid metabolism‐related genes and their main functions. [file JCMM-22-1805-s002.docx]

**Supplementary Table 1 (S1):** List of obesity and lipid metabolism related genes and its main functions

| **Gene** | **Description** | **Function** |
| --- | --- | --- |
| **ACACA** | Acetyl-CoA Carboxylase Alpha | Catalyzes the carboxylation of acetyl-CoA to malonyl-CoA, the rate-limiting step in fatty acid synthesis |
| **ACACB** | Acetyl-CoA Carboxylase Beta | Catalyzes the carboxylation of acetyl-CoA to malonyl-CoA, the rate-limiting step in fatty acid synthesis |
| **ACAT1** | Acetyl-CoA Acetyltransferase 1 | Catalyzes the reversible formation of acetoacetyl-CoA from two molecules of acetyl-CoA |
| **ACSL1** | Acyl-CoA Synthetase Long-Chain Family Member 1 | Converts free long-chain fatty acids into fatty acyl-CoA esters, and thereby play a key role in lipid biosynthesis and fatty acid degradation |
| **ACSS2** | Acyl-CoA Synthetase Short-Chain Family Member 2 | Catalyzes the activation of acetate for use in lipid synthesis and energy generation |
| **ADIPOQ** | Adiponectin, C1Q And Collagen Domain Containing | Involved with metabolic and hormonal processes |
| **AGPAT2** | 1-Acylglycerol-3-Phosphate O-Acyltransferase 2 | Converts lysophosphatidic acid to phosphatidic acid, the second step in de novo phospholipid biosynthesis |
| **AGRP** | Agouti Related Neuropeptide | Regulates hypothalamic control of feeding behavior via melanocortin receptor and/or intracellular calcium regulation, and thus plays a role in weight homeostasis |
| **AGT** | Angiotensinogen | Involved in maintaining blood pressure and in the pathogenesis of essential hypertension |
| **AHR** | Aryl Hydrocarbon Receptor | Is a ligand-activated helix-loop-helix transcription factor involved in the regulation of biological responses to planar aromatic hydrocarbons |
| **AHSG** | Alpha 2-HS Glycoprotein | Involved in several functions, such as endocytosis, brain development and the formation of bone tissue |
| **AQP7** | Aquaporin 7 | Highly expressed in the adipose tissue where the encoded protein facilitates efflux of glycerol |
| **BMP1** | Bone Morphogenetic Protein 1 | Protein that is capable of inducing formation of cartilage in vivo |
| **BMP2** | Bone Morphogenetic Protein 2 | Encodes a secreted ligand of the TGF-beta (transforming growth factor-beta) superfamily of proteins |
| **BMP3** | Bone Morphogenetic Protein 3 | Encodes a secreted ligand of the TGF-beta (transforming growth factor-beta) superfamily of proteins |
| **BMP4** | Bone Morphogenetic Protein 4 | Encodes a secreted ligand of the TGF-beta (transforming growth factor-beta) superfamily of proteins |
| **BSCL2** | BSCL2, Seipin Lipid Droplet Biogenesis Associated | This protein localizes to the endoplasmic reticulum and may be important for lipid droplet morphology |
| **CD36** | CD36 Molecule | Binds long chain fatty acids and may function in the transport and/or as a regulator of fatty acid transport |
| **CEBPA** | CCAAT/Enhancer Binding Protein Alpha | Modulates the expression of genes involved in cell cycle regulation as well as in body weight homeostasis |
| **CEBPB** | CCAAT/Enhancer Binding Protein Beta | This protein is important in the regulation of genes involved in immune and inflammatory responses |
| **CEBPD** | CCAAT/Enhancer Binding Protein Delta | This protein is important in the regulation of genes involved in immune and inflammatory responses |
| **CELF1** | CUGBP, Elav-Like Family Member 1 | Regulates pre-mRNA alternative splicing and may also be involved in mRNA editing, and translation |
| **CETP** | Cholesteryl Ester Transfer Protein | Involved in the transfer of cholesteryl ester from high density lipoprotein (HDL) to other lipoproteins |
| **CFD** | Complement Factor D | Functions as an adipokine, a cell signaling protein secreted by adipocytes, which regulates insulin secretion |
| **CNTFR** | Ciliary Neurotrophic Factor Receptor | Plays a critical role in neuronal cell survival, differentiation and gene expression |
| **CREB1** | cAMP Responsive Element Binding Protein 1 | Induces transcription of genes in response to hormonal stimulation of the cAMP pathway |
| **CYP26A1** | Cytochrome P450 Family 26 Subfamily A Member 1 | Monooxygenase which catalyzes many reactions involved in drug metabolism and synthesis of cholesterol, steroids and other lipids |
| **CYP26B1** | Cytochrome P450 Family 26 Subfamily B Member 1 | Monooxygenase which catalyzes many reactions involved in drug metabolism and synthesis of cholesterol, steroids and other lipids |
| **DDIT3** | DNA Damage Inducible Transcript 3 | Implicated in adipogenesis and erythropoiesis, is activated by endoplasmic reticulum stress, and promotes apoptosis |
| **DGAT1** | Diacylglycerol O-Acyltransferase 1 | Key metabolic enzyme, its activity may be associated with obesity and other metabolic diseases |
| **DLK1** | Delta Like Non-Canonical Notch Ligand 1 | Involved in the differentiation of several cell types including adipocytes |
| **HLA-DQA1** | Major Histocompatibility Complex, Class II, DQ Alpha 1 | Plays a central role in the immune system by presenting peptides derived from extracellular proteins |
| **HLA-DQB1** | Major Histocompatibility Complex, Class II, DQ Beta 1 | Plays a central role in the immune system by presenting peptides derived from extracellular proteins |
| **HLA-DRB5** | Major Histocompatibility Complex, Class II, DR Beta 5 | Plays a central role in the immune system by presenting peptides derived from extracellular proteins |
| **DVL1** | Dishevelled Segment Polarity Protein 1 | Regulates cell proliferation, acting as a transducer molecule for developmental processes, including segmentation and neuroblast specification |
| **E2F1** | E2F Transcription Factor 1 | Can mediate both cell proliferation and p53-dependent/independent apoptosis |
| **E2F4** | E2F Transcription Factor 4 | Plays an important role in the suppression of proliferation-associated genes, and its gene mutation and increased expression may be associated with human cancer |
| **EBF1** | Early B-Cell Factor 1 | Among its related pathways are Regulation of lipid metabolism by Peroxisome proliferator-activated receptor alpha (PPARalpha) and Developmental Biology |
| **EGR2** | Early Growth Response 2 | Is a transcription factor with three tandem C2H2-type zinc fingers |
| **ENPP2** | Ectonucleotide Pyrophosphatase/Phosphodiesterase 2 | Phosphodiesterase and a phospholipase, which catalyzes production of lysophosphatidic acid (LPA) in extracellular fluids |
| **EPAS1** | Endothelial PAS Domain Protein 1 | Transcription factor involved in the induction of genes regulated by oxygen, which is induced as oxygen levels fall |
| **FABP4** | Fatty Acid Binding Protein 4 | Cytoplasmic protein that binds long-chain fatty acids and other hydrophobic ligands, found in adipocytes |
| **FASN** | Fatty Acid Synthase | Catalyzes the synthesis of palmitate from acetyl-CoA and malonyl-CoA, in the presence of NADPH, into long-chain saturated fatty acids |
| **FGF21** | Fibroblast Growth Factor 21 | Is a secreted endocrine factor that functions as a major metabolic regulator |
| **FOXC2** | Forkhead Box C2 | It may play a role in the development of mesenchymal tissues |
| **FOXO1** | Forkhead Box O1 | It may play a role in myogenic growth and differentiation |
| **FTO** | FTO, Alpha-Ketoglutarate Dependent Dioxygenase | Function unknown, studies in mice and humans indicate a role in nervous and cardiovascular systems and a strong association with body mass index, obesity risk, and type 2 diabetes |
| **GADD45A** | Growth Arrest And DNA Damage Inducible Alpha | Is a member of a group of genes whose transcript levels are increased following stressful growth arrest conditions and treatment with DNA-damaging agents |
| **GATA2** | GATA Binding Protein 2 | Plays an essential role in regulating transcription of genes involved in the development and proliferation of hematopoietic and endocrine cell lineages |
| **GATA3** | GATA Binding Protein 3 | Is an important regulator of T-cell development and plays an important role in endothelial cell biology |
| **GATA4** | GATA Binding Protein 4 | Is thought to regulate genes involved in embryogenesis and in myocardial differentiation and function, and is necessary for normal testicular development |
| **GH1** | Growth Hormone 1 | Is a member of the somatotropin/prolactin family of hormones which play an important role in growth control |
| **GINS3** | GINS Complex Subunit 3 | Is essential for the initiation of DNA replication and replisome progression in eukaryotes |
| **GK** | Glycerol Kinase | Is a key enzyme in the regulation of glycerol uptake and metabolism |
| **HIF1A** | Hypoxia Inducible Factor 1 Alpha Subunit | Master regulator of cellular and systemic homeostatic response to hypoxia by activating transcription of many genes, including those involved in energy metabolism, angiogenesis, apoptosis, and other genes whose protein products increase oxygen delivery or facilitate metabolic adaptation to hypoxia |
| **HMGA1** | High Mobility Group AT-Hook 1 | Involved in the regulation of gene transcription, integration of retroviruses into chromosomes, and the metastatic progression of cancer cells |
| **HNF1A** | HNF1 Homeobox A | Is a transcription factor required for the expression of several liver-specific genes |
| **ID3** | Inhibitor Of DNA Binding 3, HLH Protein | Inhibits the DNA binding of any HLH protein with which it interacts |
| **IGF1** | Insulin Like Growth Factor 1 | Is similar to insulin in function and structure and is a member of a family of proteins involved in mediating growth and development |
| **IL17A** | Interleukin 17A | This cytokine can stimulate the expression of IL6 and cyclooxygenase-2 (PTGS2/COX-2), as well as enhance the production of nitric oxide (NO) |
| **IL1B** | Interleukin 1 Beta | This cytokine is an important mediator of the inflammatory response, and is involved in a variety of cellular activities, including cell proliferation, differentiation, and apoptosis |
| **IL22** | Interleukin 22 | Cytokine that contributes to the inflammatory response in vivo |
| **IL6** | Interleukin 6 | Cytokine that functions in inflammation and the maturation of B cells |
| **IL6R** | Interleukin 6 Receptor | Is a potent pleiotropic cytokine that regulates cell growth and differentiation and plays an important role in the immune response |
| **IL6ST** | Interleukin 6 Signal Transducer | Is a signal transducer shared by many cytokines, including interleukin 6 (IL6), ciliary neurotrophic factor (CNTF), leukemia inhibitory factor (LIF), and oncostatin M (OSM) |
| **INS** | Insulin | Binding of insulin to the insulin receptor (INSR) stimulates glucose uptake |
| **IRS1** | Insulin Receptor Substrate 1 | May mediate the control of various cellular processes by insulin |
| **IRS2** | Insulin Receptor Substrate 2 | A cytoplasmic signaling molecule that mediates effects of insulin, insulin-like growth factor 1, and other cytokines by acting as a molecular adaptor between diverse receptor tyrosine kinases and downstream effectors |
| **IRS4** | Insulin Receptor Substrate 4 | Has been shown to associate with cytoplasmic signalling molecules that contain SH2 domains |
| **KLF15** | Kruppel Like Factor 15 | Is a negative regulator of TP53 acetylation. Inhibits NF-kappa-B activation through repression of EP300-dependent RELA acetylation |
| **KLF5** | Kruppel Like Factor 5 | This protein acts downstream of multiple different signaling pathways and is regulated by post-translational modification. It may participate in both promoting and suppressing cell proliferation. |
| **KLF6** | Kruppel Like Factor 6 | The zinc finger protein is a transcriptional activator, and functions as a tumor suppressor |
| **KLF7** | Kruppel Like Factor 7 | This protein may contribute to the progression of type 2 diabetes by inhibiting insulin expression and secretion in pancreatic beta-cells and by deregulating adipocytokine secretion in adipocytes |
| **LACTB** | Lactamase Beta | Increased expression of the related mouse gene was found to be associated with obesity |
| **LEP** | Leptin | This protein, which acts through the leptin receptor, functions as part of a signaling pathway that can inhibit food intake and/or regulate energy expenditure to maintain constancy of the adipose mass |
| **LIF** | Leukemia Inhibitory Factor | Is a pleiotropic cytokine with roles in several different systems. It is involved in the induction of hematopoietic differentiation in normal and myeloid leukemia cells |
| **LIFR** | Leukemia Inhibitory Factor Receptor Alpha | This protein combines with a high-affinity converter subunit, gp130, to form a receptor complex that mediates the action of the leukemia inhibitory factor, a polyfunctional cytokine that is involved in cellular differentiation, proliferation and survival in the adult and the embryo |
| **LIPE** | Lipase E, Hormone Sensitive Type | The long form is expressed in steroidogenic tissues such as testis, where it converts cholesteryl esters to free cholesterol for steroid hormone production. The short form is expressed in adipose tissue, among others, where it hydrolyzes stored triglycerides to free fatty acids |
| **LMNA** | Lamin A/C | Lamin proteins are thought to be involved in nuclear stability, chromatin structure and gene expression |
| **LPIN1** | Lipin 1 | Expression of this gene is required for adipocyte differentiation and it also functions as a nuclear transcriptional coactivator with some peroxisome proliferator-activated receptors to modulate expression of other genes involved in lipid metabolism |
| **LPIN2** | Lipin 2 | This gene functions during normal adipose tissue development and may play a role in human triglyceride metabolism |
| **LPIN3** | Lipin 3 | Lipin complexes act in the cytoplasm to catalyze the dephosphorylation of phosphatidic acid to produce diacylglycerol, which is the precursor of both triglycerides and phospholipids |
| **LPL** | Lipoprotein Lipase | functions as a homodimer, and has the dual functions of triglyceride hydrolase and ligand/bridging factor for receptor-mediated lipoprotein uptake |
| **MEF2A** | Myocyte Enhancer Factor 2A | Can act as a homodimer or as a heterodimer and is involved in several cellular processes, including muscle development, neuronal differentiation, cell growth control, and apoptosis |
| **MEF2B** | Myocyte Enhancer Factor 2B | Is thought to regulate gene expression, including expression of the smooth muscle myosin heavy chain gene |
| **MEF2C** | Myocyte Enhancer Factor 2C | May play a role in maintaining the differentiated state of muscle cells |
| **MEF2D** | Myocyte Enhancer Factor 2D | Involved in control of muscle and neuronal cell differentiation and development, and are regulated by class II histone deacetylases |
| **MIF** | Macrophage Migration Inhibitory Factor (Glycosylation-Inhibiting Factor) | It plays a role in the regulation of macrophage function in host defense through the suppression of anti-inflammatory effects of glucocorticoids |
| **MKKS** | McKusick-Kaufman Syndrome | Is a centrosome-shuttling protein and plays an important role in cytokinesis |
| **MMP9** | Matrix Metallopeptidase 9 | Involved in the breakdown of extracellular matrix in normal physiological processes, such as embryonic development, reproduction, and tissue remodeling, as well as in disease processes, such as arthritis and metastasis |
| **NAMPT** | Nicotinamide Phosphoribosyltransferase | The protein belongs to the nicotinic acid phosphoribosyltransferase (NAPRTase) family and is thought to be involved in many important biological processes, including metabolism, stress response and aging |
| **NCOA1** | Nuclear Receptor Coactivator 1 | Stimulates the transcriptional activities in a hormone-dependent fashion |
| **NCOR1** | Nuclear Receptor Corepressor 1 | Mediates ligand-independent transcription repression of thyroid-hormone and retinoic-acid receptors by promoting chromatin condensation and preventing access of the transcription machinery |
| **NCOR2** | Nuclear Receptor Corepressor 2 | Nuclear receptor co-repressor that mediates transcriptional silencing of certain target genes |
| **NDN** | Necdin, MAGE Family Member | May suppress growth in postmitotic neurons |
| **NDRG4** | NDRG Family Member 4 | Is a cytoplasmic protein that is required for cell cycle progression and survival in primary astrocytes and may be involved in the regulation of mitogenic signalling in vascular smooth muscles cells |
| **NLRP3** | NLR Family Pyrin Domain Containing 3 | Functions as an upstream activator of NF-kappaB signaling, and it plays a role in the regulation of inflammation, the immune response, and apoptosis |
| **NR1H3** | Nuclear Receptor Subfamily 1 Group H Member 3 | Key regulators of macrophage function, controlling transcriptional programs involved in lipid homeostasis and inflammation |
| **NR2F1** | Nuclear Receptor Subfamily 2 Group F Member 1 | Is a nuclear hormone receptor and transcriptional regulator |
| **NR3C1** | Nuclear Receptor Subfamily 3 Group C Member 1 | It is involved in inflammatory responses, cellular proliferation, and differentiation in target tissues |
| **NRIP1** | Nuclear Receptor Interacting Protein 1 | Modulates transcriptional activity of the estrogen receptor |
| **OSM** | Oncostatin M | Is a secreted cytokine and growth regulator that inhibits the proliferation of a number of tumor cell lines |
| **PCK1** | Phosphoenolpyruvate Carboxykinase 1 | This gene can be regulated by insulin, glucocorticoids, glucagon, cAMP, and diet |
| **PCK2** | Phosphoenolpyruvate Carboxykinase 2, Mitochondrial | Enzyme that catalyzes the conversion of oxaloacetate to phosphoenolpyruvate in the presence of guanosine triphosphate (GTP) |
| **PLIN1** | Perilipin 1 | Coats lipid storage droplets in adipocytes, thereby protecting them until they can be broken down by hormone-sensitive lipase |
| **PLIN2** | Perilipin 2 | Is associated with the lipid globule surface membrane material, and maybe involved in development and maintenance of adipose tissue |
| **PNPLA2** | Patatin Like Phospholipase Domain Containing 2 | Catalyzes the first step in the hydrolysis of triglycerides in adipose tissue |
| **PNPLA3** | Patatin Like Phospholipase Domain Containing 3 | Is a triacylglycerol lipase that mediates triacylglycerol hydrolysis in adipocytes |
| **PPARA** | Peroxisome Proliferator Activated Receptor Alpha | PPARs affect the expression of target genes involved in cell proliferation, cell differentiation and in immune and inflammation responses |
| **PPARD** | Peroxisome Proliferator Activated Receptor Delta | PPARs mediate a variety of biological processes, and may be involved in the development of several chronic diseases, including diabetes, obesity, atherosclerosis, and cancer |
| **PPARG** | Peroxisome Proliferator Activated Receptor Gamma | Is a regulator of adipocyte differentiation |
| **PPARGC1A** | PPARG Coactivator 1 Alpha | Is a transcriptional coactivator that regulates the genes involved in energy metabolism |
| **PRLR** | Prolactin Receptor | Receptor for the anterior pituitary hormone, prolactin, may function to modulate the endocrine and autocrine effects of prolactin in normal tissue and cancer |
| **PTGIS** | Prostaglandin I2 Synthase | The cytochrome P450 proteins are monooxygenases which catalyze many reactions involved in drug metabolism and synthesis of cholesterol, steroids and other lipids |
| **PTPRE** | Protein Tyrosine Phosphatase, Receptor Type E | PTPs are known to be signaling molecules that regulate a variety of cellular processes including cell growth, differentiation, mitotic cycle, and oncogenic transformation |
| **RARA** | Retinoic Acid Receptor Alpha | This gene has been implicated in regulation of development, differentiation, apoptosis, granulopoeisis, and transcription of clock genes |
| **RBL1** | RB Transcriptional Corepressor Like 1 | Due to the sequence and biochemical similarities with the RB1 protein, it is thought that the protein encoded by this gene may also be a tumor suppressor |
| **RBL2** | RB Transcriptional Corepressor Like 2 | Key regulator of entry into cell division |
| **RETN** | Resistin | Is secreted by adipocytes, and may be the hormone potentially linking obesity to type II diabetes |
| **RORA** | RAR Related Orphan Receptor A | Is a member of the NR1 subfamily of nuclear hormone receptors |
| **RXRA** | Retinoid X Receptor Alpha | Nuclear receptor that mediates the biological effects of retinoids by their involvement in retinoic acid-mediated gene activation |
| **RXRG** | Retinoid X Receptor Gamma | Nuclear receptor which is involved in mediating the antiproliferative effects of retinoic acid (RA) |
| **SCD** | Stearoyl-CoA Desaturase | Enzyme involved in fatty acid biosynthesis, primarily the synthesis of oleic acid |
| **SERPINE1** | Serpin Family E Member 1 | Is the principal inhibitor of tissue plasminogen activator (tPA) and urokinase (uPA), and hence is an inhibitor of fibrinolysis |
| **SFRP4** | Secreted Frizzled Related Protein 4 | Acts as soluble modulators of Wnt signaling |
| **SLC2A2** | Solute Carrier Family 2 Member 2 | Mediates facilitated bidirectional glucose transport. Because of its low affinity for glucose, it has been suggested as a glucose sensor |
| **SLC6A5** | Solute Carrier Family 6 Member 5 | Is responsible for the clearance of extracellular glycine during glycine-mediated neurotransmission |
| **SMAD3** | SMAD Family Member 3 | This protein functions as a transcriptional modulator activated by transforming growth factor-beta and is thought to play a role in the regulation of carcinogenesis |
| **SOCS3** | Suppressor Of Cytokine Signaling 3 | SSI family members are cytokine-inducible negative regulators of cytokine signaling |
| **SP1** | Sp1 Transcription Factor | Involved in many cellular processes, including cell differentiation, cell growth, apoptosis, immune responses, response to DNA damage, and chromatin remodeling |
| **SPP1** | Secreted Phosphoprotein 1 | Is involved in the attachment of osteoclasts to the mineralized bone matrix |
| **SREBF1** | Sterol Regulatory Element Binding Transcription Factor 1 | Transcription factor that binds to the sterol regulatory element-1 (SRE1), which is a decamer flanking the low density lipoprotein receptor gene and some genes involved in sterol biosynthesis |
| **STAT1** | Signal Transducer And Activator Of Transcription 1 | This protein mediates the expression of a variety of genes, which is thought to be important for cell viability in response to different cell stimuli and pathogens |
| **STAT3** | Signal Transducer And Activator Of Transcription 3 | This protein mediates the expression of a variety of genes in response to cell stimuli, and thus plays a key role in many cellular processes such as cell growth and apoptosis |
| **STAT5A** | Signal Transducer And Activator Of Transcription 5A | Activation of this protein in myeloma and lymphoma associated with a TEL/JAK2 gene fusion is independent of cell stimulus and has been shown to be essential for tumorigenesis |
| **TGFB1** | Transforming Growth Factor Beta 1 | Regulates cell proliferation, differentiation and growth, and can modulate expression and activation of other growth factors including interferon gamma and tumor necrosis factor alpha |
| **TLR4** | Toll Like Receptor 4 | Plays a fundamental role in pathogen recognition and activation of innate immunity |
| **TRIB3** | Tribbles Pseudokinase 3 | Is a negative regulator of NF-kappaB and can also sensitize cells to TNF- and TRAIL-induced apoptosis |
| **TWIST1** | Twist Family BHLH Transcription Factor 1 | Basic helix-loop-helix (bHLH) transcription factors have been implicated in cell lineage determination and differentiation |
| **UCP1** | Uncoupling Protein 1 | UCPs separate oxidative phosphorylation from ATP synthesis with energy dissipated as heat, also referred to as the mitochondrial proton leak |
| **UCP2** | Uncoupling Protein 2 | UCPs facilitate the transfer of anions from the inner to the outer mitochondrial membrane and the return transfer of protons from the outer to the inner mitochondrial membrane |
| **WNT1** | Wnt Family Member 1 | Implicated in oncogenesis and in several developmental processes, including regulation of cell fate and patterning during embryogenesis |
| **WNT10B** | Wnt Family Member 10B | It may be involved in breast cancer, and its protein signaling is likely a molecular switch that governs adipogenesis |
| **WNT5B** | Wnt Family Member 5B | Implicated in oncogenesis and in several developmental processes, including regulation of cell fate and patterning during embryogenesis |

*Built up using GeneCards®: The human gene database (Weizmann Institute of Science. v4.4.2 Build 18). Accessible through http://www.genecards.org/
