# Supplementary material for: The impact on high‐grade serous ovarian cancer of obesity and lipid metabolism‐related gene expression patterns: the underestimated driving force affecting prognosis
Source: J Cell Mol Med. 2017 Dec 20;22(3):1805–15. doi: 10.1111/jcmm.13463 (PMC5824367; doi:10.1111/jcmm.13463)
Supplement: Supplementary file 3 — Table S2 Statistical analysis of differences in the distribution of robust RNAseq, copy number alteration, methylation and RPPA subgroups between the two clusters obtained by NMF analysis using obesity and lipid metabolism‐related gene expression. [file JCMM-22-1805-s003.docx]

**Supplementary Table 2 (S2):** Significant Obesity and lipid metabolism-related genes for US-OVCA Cohort NMF Clustering (RNA microarray data)

| **Rank** | **Feature** | **Description** | **Upregulated in Cluster [C]** | **Feature P** | **FDR(BH)** | **Q Value** |
| --- | --- | --- | --- | --- | --- | --- |
| 25 | 100271849 | MEF2B | **G1** | 0,00020 | 0,00062 | 0,00011 |
| 26 | 55902 | ACSS2 | **G1** | 0,00020 | 0,00062 | 0,00011 |
| 31 | 6927 | HNF1A | **G1** | 0,00040 | 0,00109 | 0,00011 |
| 33 | 7350 | UCP1 | **G1** | 0,00040 | 0,00109 | 0,00011 |
| 36 | 5618 | PRLR | **G1** | 0,00040 | 0,00109 | 0,00011 |
| 41 | 6258 | RXRG | **G1** | 0,00040 | 0,00109 | 0,00011 |
| 42 | 8195 | MKKS | **G1** | 0,00040 | 0,00109 | 0,00011 |
| 45 | 3159 | HMGA1 | **G1** | 0,00140 | 0,00314 | 0,00030 |
| 49 | 5106 | PCK2 | **G1** | 0,00180 | 0,00391 | 0,00037 |
| 51 | 1855 | DVL1 | **G1** | 0,00080 | 0,00199 | 0,00019 |
| 53 | 57761 | TRIB3 | **G1** | 0,00180 | 0,00391 | 0,00037 |
| 54 | 1869 | E2F1 | **G1** | 0,00100 | 0,00236 | 0,00022 |
| 55 | 3570 | IL6R | **G1** | 0,00080 | 0,00199 | 0,00019 |
| 58 | 5933 | RBL1 | **G1** | 0,00140 | 0,00314 | 0,00030 |
| 61 | 64900 | LPIN3 | **G1** | 0,00260 | 0,00531 | 0,00050 |
| 62 | 2688 | GH1 | **G1** | 0,00300 | 0,00587 | 0,00055 |
| 65 | 2626 | GATA4 | **G1** | 0,00260 | 0,00531 | 0,00050 |
| 66 | 2624 | GATA2 | **G1** | 0,00280 | 0,00556 | 0,00052 |
| 67 | 3977 | LIFR | **G1** | 0,00380 | 0,00723 | 0,00068 |
| 68 | 197 | AHSG | **G1** | 0,00600 | 0,01097 | 0,00103 |
| 69 | 3605 | IL17A | **G1** | 0,00480 | 0,00901 | 0,00085 |
| 73 | 1271 | CNTFR | **G1** | 0,00780 | 0,01408 | 0,00133 |
| 77 | 8204 | NRIP1 | **G1** | 0,01000 | 0,01737 | 0,00164 |
| 16 | 1649 | DDIT3 | **G1** | 0,00020 | 0,00062 | 0,00011 |
| 85 | 2908 | NR3C1 | **G2** | 0,00940 | 0,01654 | 0,00156 |
| 82 | 181 | AGRP | **G2** | 0,00800 | 0,01425 | 0,00134 |
| 79 | 56603 | CYP26B1 | **G2** | 0,00580 | 0,01075 | 0,00101 |
| 78 | 688 | KLF5 | **G2** | 0,00220 | 0,00470 | 0,00044 |
| 76 | 8609 | KLF7 | **G2** | 0,00380 | 0,00723 | 0,00068 |
| 74 | 6774 | STAT3 | **G2** | 0,00140 | 0,00314 | 0,00030 |
| 72 | 4205 | MEF2A | **G2** | 0,00260 | 0,00531 | 0,00050 |
| 71 | 2308 | FOXO1 | **G2** | 0,00100 | 0,00236 | 0,00022 |
| 70 | 8694 | DGAT1 | **G2** | 0,00280 | 0,00556 | 0,00052 |
| 64 | 4000 | LMNA | **G2** | 0,00060 | 0,00154 | 0,00015 |
| 63 | 5468 | PPARG | **G2** | 0,00040 | 0,00109 | 0,00011 |
| 60 | 8660 | IRS2 | **G2** | 0,00060 | 0,00154 | 0,00015 |
| 59 | 4023 | LPL | **G2** | 0,00060 | 0,00154 | 0,00015 |
| 57 | 3991 | LIPE | **G2** | 0,00020 | 0,00062 | 0,00011 |
| 56 | 10135 | NAMPT | **G2** | 0,00100 | 0,00236 | 0,00022 |
| 52 | 3399 | ID3 | **G2** | 0,00020 | 0,00062 | 0,00011 |
| 50 | 9612 | NCOR2 | **G2** | 0,00020 | 0,00062 | 0,00011 |
| 48 | 1647 | GADD45A | **G2** | 0,00020 | 0,00062 | 0,00011 |
| 47 | 364 | AQP7 | **G2** | 0,00020 | 0,00062 | 0,00011 |
| 46 | 650 | BMP2 | **G2** | 0,00020 | 0,00062 | 0,00011 |
| 44 | 3572 | IL6ST | **G2** | 0,00020 | 0,00062 | 0,00011 |
| 43 | 1051 | CEBPB | **G2** | 0,00020 | 0,00062 | 0,00011 |
| 40 | 3976 | LIF | **G2** | 0,00020 | 0,00062 | 0,00011 |
| 39 | 7040 | TGFB1 | **G2** | 0,00020 | 0,00062 | 0,00011 |
| 38 | 4318 | MMP9 | **G2** | 0,00020 | 0,00062 | 0,00011 |
| 37 | 2034 | EPAS1 | **G2** | 0,00020 | 0,00062 | 0,00011 |
| 35 | 1071 | CETP | **G2** | 0,00020 | 0,00062 | 0,00011 |
| 34 | 114294 | LACTB | **G2** | 0,00020 | 0,00062 | 0,00011 |
| 32 | 7291 | TWIST1 | **G2** | 0,00020 | 0,00062 | 0,00011 |
| 30 | 652 | BMP4 | **G2** | 0,00020 | 0,00062 | 0,00011 |
| 29 | 948 | CD36 | **G2** | 0,00020 | 0,00062 | 0,00011 |
| 28 | 649 | BMP1 | **G2** | 0,00020 | 0,00062 | 0,00011 |
| 27 | 1675 | CFD | **G2** | 0,00020 | 0,00062 | 0,00011 |
| 24 | 3952 | LEP | **G2** | 0,00020 | 0,00062 | 0,00011 |
| 23 | 9021 | SOCS3 | **G2** | 0,00020 | 0,00062 | 0,00011 |
| 22 | 56729 | RETN | **G2** | 0,00020 | 0,00062 | 0,00011 |
| 21 | 5791 | PTPRE | **G2** | 0,00020 | 0,00062 | 0,00011 |
| 20 | 2180 | ACSL1 | **G2** | 0,00020 | 0,00062 | 0,00011 |
| 19 | 1879 | EBF1 | **G2** | 0,00020 | 0,00062 | 0,00011 |
| 18 | 3553 | IL1B | **G2** | 0,00020 | 0,00062 | 0,00011 |
| 17 | 3479 | IGF1 | **G2** | 0,00020 | 0,00062 | 0,00011 |
| 15 | 1592 | CYP26A1 | **G2** | 0,00020 | 0,00062 | 0,00011 |
| 14 | 6696 | SPP1 | **G2** | 0,00020 | 0,00062 | 0,00011 |
| 13 | 4208 | MEF2C | **G2** | 0,00020 | 0,00062 | 0,00011 |
| 12 | 7099 | TLR4 | **G2** | 0,00020 | 0,00062 | 0,00011 |
| 11 | 5346 | PLIN1 | **G2** | 0,00020 | 0,00062 | 0,00011 |
| 10 | 5008 | OSM | **G2** | 0,00020 | 0,00062 | 0,00011 |
| 9 | 1316 | KLF6 | **G2** | 0,00020 | 0,00062 | 0,00011 |
| 8 | 1959 | EGR2 | **G2** | 0,00020 | 0,00062 | 0,00011 |
| 7 | 3569 | IL6 | **G2** | 0,00020 | 0,00062 | 0,00011 |
| 6 | 114548 | NLRP3 | **G2** | 0,00020 | 0,00062 | 0,00011 |
| 5 | 5054 | SERPINE1 | **G2** | 0,00020 | 0,00062 | 0,00011 |
| 4 | 9370 | ADIPOQ | **G2** | 0,00020 | 0,00062 | 0,00011 |
| 3 | 2167 | FABP4 | **G2** | 0,00020 | 0,00062 | 0,00011 |
| 2 | 6424 | SFRP4 | **G2** | 0,00020 | 0,00062 | 0,00011 |
| 1 | 5740 | PTGIS | **G2** | 0,00020 | 0,00062 | 0,00011 |
