# Supplementary material for: The impact on high‐grade serous ovarian cancer of obesity and lipid metabolism‐related gene expression patterns: the underestimated driving force affecting prognosis
Source: J Cell Mol Med. 2017 Dec 20;22(3):1805–15. doi: 10.1111/jcmm.13463 (PMC5824367; doi:10.1111/jcmm.13463)
Supplement: Supplementary file 4 — Table S3 Significant Obesity and lipid metabolism‐related genes for clustering [file JCMM-22-1805-s004.docx]

**Supplementary Table 3 (S3):** Statistical analysis of differences in distribution of robust RNAseq, copy number alteration, methylation and RPPA subgroups between the two clusters obtained by NMF analysis using obesity and lipid metabolism related gene expression.

|  |  |  |  |  |  |  |  |
| --- | --- | --- | --- | --- | --- | --- | --- |
|  |  |  | **NMF clustering by obesity/metabolism-related genes** | | |  |  |
|  |  |  | **cluster 1** |  | **cluster 2** | **p-value** |  |
|  |  |  |  |  |  |  |  |
|  | **RNAseq clustering** | |  |  |  |  |  |
|  | n |  | 116 |  | 170 |  |  |
|  |  | I | 38 (32.8%) |  | 102 (60%) | <0.0001 |  |
|  |  | II | 7 (6%) |  | 44 (25.9%) |  |  |
|  |  | III | 71 (61.2%) |  | 24 (14.1%) |  |  |
|  |  |  |  |  |  |  |  |
|  | **CNA clustering** | |  |  |  |  |  |
|  | n |  | 178 |  | 342 |  |  |
|  |  | I | 60 (33.7%) |  | 158 (46.2%) | 0.002 |  |
|  |  | II | 55 (30.9%) |  | 62 (18.1%) |  |  |
|  |  | III | 63 (35.4%) |  | 122 (35.7%) |  |  |
|  |  |  |  |  |  |  |  |
|  | **Methylation clustering** | |  |  |  |  |  |
|  | n |  | 186 |  | 350 |  |  |
|  |  | I | 53 (28.5%) |  | 58 (16.6%) | 0.0001 |  |
|  |  | II | 32 (17.2%) |  | 57 (16.3%) |  |  |
|  |  | III | 77 (41.4%) |  | 100 (28.6%) |  |  |
|  |  | IV | 24 (12.9%) |  | 135 (38.6%) |  |  |
|  |  |  |  |  |  |  |  |
|  | **RPPA clustering** | |  |  |  |  |  |
|  | n |  | 138 |  | 261 |  |  |
|  |  | I | 78 (56.5%) |  | 63 (24.1%) | <0.0001 |  |
|  |  | II | 20 (14.5%) |  | 34 (13%) |  |  |
|  |  | III | 14 (10.1%) |  | 43 (16.5%) |  |  |
|  |  | IV | 26 (18.8%) |  | 121 (46.4%) |  |  |
|  |  |  |  |  |  |  |  |
