# Supplementary material for: The impact on high‐grade serous ovarian cancer of obesity and lipid metabolism‐related gene expression patterns: the underestimated driving force affecting prognosis
Source: J Cell Mol Med. 2017 Dec 20;22(3):1805–15. doi: 10.1111/jcmm.13463 (PMC5824367; doi:10.1111/jcmm.13463)
Supplement: Supplementary file 5 — Table S4 List of cancer driver genes and their functions [file JCMM-22-1805-s005.docx]

**Supplementary Table 4 (S4):** List of cancer driver genes and its functions

| **GENE_ID** | **Gene** | **Description** | **Function** |
| --- | --- | --- | --- |
| 48 | **ACO1** | Aconitase 1 | Functions as an essential enzyme in the TCA cycle and interacts with mRNA to control the levels of iron inside cells |
| 71 | **ACTG1** | Actin Gamma 1 | Involved in various types of cell motility, and maintenance of the cytoskeleton |
| 23266 | **ADGRL2** | Adhesion G Protein-Coupled Receptor L2 | Participates in the regulation of exocytosis |
| 27125 | **AFF4** | AF4/FMR2 Family Member 4 | Belongs to the AF4 family of transcription factors involved in leukemia |
| 139285 | **AMER1** | APC Membrane Recruitment Protein 1 | His gene upregulates trancriptional activation by the Wilms tumor protein and interacts with many other proteins, including CTNNB1, APC, AXIN1, and AXIN2 |
| 8289 | **ARID1A** | AT-Rich Interaction Domain 1A | Thought to regulate transcription of certain genes by altering the chromatin structure around those genes |
| 55870 | **ASH1L** | ASH1 Like Histone Lysine Methyltransferase | A member of the trithorax group of transcriptional activators |
| 259266 | **ASPM** | Abnormal Spindle Microtubule Assembly | Essential for normal mitotic spindle function in embryonic neuroblasts |
| 466 | **ATF1** | Activating Transcription Factor 1 | It influences cellular physiologic processes by regulating the expression of downstream target genes, which are related to growth, survival, and other cellular activities |
| 471 | **ATIC** | 5-Aminoimidazole-4-Carboxamide Ribonucleotide Formyltransferase/IMP Cyclohydrolase | Catalyzes the last two steps of the de novo purine biosynthetic pathway |
| 545 | **ATR** | ATR Serine/Threonine Kinase | This kinase has been shown to phosphorylate checkpoint kinase CHK1, checkpoint proteins RAD17, and RAD9, as well as tumor suppressor protein BRCA1 |
| 546 | **ATRX** | ATRX, Chromatin Remodeler | This protein is found to undergo cell cycle-dependent phosphorylation, which regulates its nuclear matrix and chromatin association, and suggests its involvement in the gene regulation at interphase and chromosomal segregation in mitosis |
| 8314 | **BAP1** | BRCA1 Associated Protein 1 | Binds to the breast cancer type 1 susceptibility protein (BRCA1) via the RING finger domain of the latter and acts as a tumor suppressor |
| 29994 | **BAZ2B** | Bromodomain Adjacent To Zinc Finger Domain 2B | Members of this gene family encode proteins that are integral components of chromatin remodeling complexes |
| 659 | **BMPR2** | Bone Morphogenetic Protein Receptor Type 2 | Encodes a member of the bone morphogenetic protein (BMP) receptor family of transmembrane serine/threonine kinases |
| 673 | **BRAF** | B-Raf Proto-Oncogene, Serine/Threonine Kinase | Plays a role in regulating the MAP kinase/ERKs signaling pathway, which affects cell division, differentiation, and secretion |
| 672 | **BRCA1** | BRCA1, DNA Repair Associated | Plays a role in maintaining genomic stability, and it also acts as a tumor suppressor |
| 675 | **BRCA2** | BRCA2, DNA Repair Associated | Involved in maintenance of genome stability, specifically the homologous recombination pathway for double-strand DNA repair |
| 834 | **CASP1** | Caspase 1 | Plays a central role in the execution-phase of cell apoptosis |
| 55749 | **CCAR1** | Cell Division Cycle And Apoptosis Regulator 1 | Associates with components of the Mediator and p160 coactivator complexes that play a role as intermediaries transducing regulatory signals from upstream transcriptional activator proteins to basal transcription machinery at the core promoter |
| 22948 | **CCT5** | Chaperonin Containing TCP1 Subunit 5 | Is a molecular chaperone that is a member of the chaperonin containing TCP1 complex (CCT), also known as the TCP1 ring complex (TRiC) |
| 51755 | **CDK12** | Cyclin Dependent Kinase 12 | Regulates the expression of genes involved in DNA repair and is required for the maintenance of genomic stability |
| 9557 | **CHD1L** | Chromodomain Helicase DNA Binding Protein 1 Like | DNA helicase protein involved in DNA repair |
| 1108 | **CHD4** | Chromodomain Helicase DNA Binding Protein 4 | Plays an important role in epigenetic transcriptional repression |
| 23122 | **CLASP2** | Cytoplasmic Linker Associated Protein 2 | Involved in the nucleation of noncentrosomal microtubules originating from the trans-Golgi network (TGN). Required for the polarization of the cytoplasmic microtubule arrays in migrating cells towards the leading edge of the cell |
| 63967 | **CLSPN** | Claspin | Is an essential upstream regulator of checkpoint kinase 1 and triggers a checkpoint arrest of the cell cycle in response to replicative stress or DNA damage |
| 7812 | **CSDE1** | Cold Shock Domain Containing E1 | Involved in translationally coupled mRNA turnover |
| 1499 | **CTNNB1** | Catenin Beta 1 | Is part of a complex of proteins that constitute adherens junctions (AJs). AJs are necessary for the creation and maintenance of epithelial cell layers by regulating cell growth and adhesion between cells |
| 8453 | **CUL2** | Cullin 2 | Core component of multiple cullin-RING-based ECS (ElonginB/C-CUL2/5-SOCS-box protein) E3 ubiquitin-protein ligase complexes, which mediate the ubiquitination of target proteins |
| 1655 | **DDX5** | DEAD-Box Helicase 5 | Implicated in a number of cellular processes involving alteration of RNA secondary structure, such as translation initiation, nuclear and mitochondrial splicing, and ribosome and spliceosome assembly |
| 1739 | **DLG1** | Discs Large MAGUK Scaffold Protein 1 | May have a role in septate junction formation, signal transduction, cell proliferation, synaptogenesis and lymphocyte activation |
| 1788 | **DNMT3A** | DNA Methyltransferase 3 Alpha | Encodes a DNA methyltransferase that is thought to function in de novo methylation, rather than maintenance methylation |
| 9451 | **EIF2AK3** | Eukaryotic Translation Initiation Factor 2 Alpha Kinase 3 | Phosphorylates the alpha subunit of eukaryotic translation-initiation factor 2, leading to its inactivation, and thus to a rapid reduction of translational initiation and repression of global protein synthesis |
| 1974 | **EIF4A2** | Eukaryotic Translation Initiation Factor 4A2 | ATP-dependent RNA helicase which is a subunit of the eIF4F complex involved in cap recognition and is required for mRNA binding to ribosome |
| 55914 | **ERBB2IP** | Erbb2 Interacting Protein | It binds to the unphosphorylated form of the ERBB2 protein and regulates ERBB2 function and localization. It has also been shown to affect the Ras signaling pathway by disrupting Ras-Raf interaction |
| 2157 | **F8** | Coagulation Factor VIII | Participates in the intrinsic pathway of blood coagulation; factor VIII is a cofactor for factor IXa which, in the presence of Ca+2 and phospholipids, converts factor X to the activated form Xa |
| 55294 | **FBXW7** | F-Box And WD Repeat Domain Containing 7 | Constitutes one of the four subunits of ubiquitin protein ligase complex called SCFs (SKP1-cullin-F-box), which function in phosphorylation-dependent ubiquitination |
| 2322 | **FLT3** | Fms Related Tyrosine Kinase 3 | A class III receptor tyrosine kinase that regulates hematopoiesis. The activated receptor kinase subsequently phosphorylates and activates multiple cytoplasmic effector molecules in pathways involved in apoptosis, proliferation, and differentiation of hematopoietic cells in bone marrow |
| 2332 | **FMR1** | Fragile X Mental Retardation 1 | Involved in mRNA trafficking from the nucleus to the cytoplasm |
| 2778 | **GNAS** | GNAS Complex Locus | Guanine nucleotide-binding proteins (G proteins) function as transducers in numerous signaling pathways controlled by G protein-coupled receptors (GPCRs) |
| 9950 | **GOLGA5** | Golgin A5 | This golgin is a coiled-coil membrane protein that has been postulated to play a role in vesicle tethering and docking |
| 2874 | **GPS2** | G Protein Pathway Suppressor 2 | Encodes a protein involved in G protein-mitogen-activated protein kinase (MAPK) signaling cascades |
| 8841 | **HDAC3** | Histone Deacetylase 3 | Plays a critical role in transcriptional regulation, cell cycle progression, and developmental events. Histone acetylation/deacetylation alters chromosome structure and affects transcription factor access to DNA |
| 3082 | **HGF** | Hepatocyte Growth Factor | Binds to the hepatocyte growth factor receptor to regulate cell growth, cell motility and morphogenesis in numerous cell and tissue types |
| 3320 | **HSP90AA1** | Heat Shock Protein 90 Alpha Family Class A Member 1 | Aids in the proper folding of specific target proteins by use of an ATPase activity that is modulated by co-chaperones |
| 6453 | **ITSN1** | Intersectin 1 | Is a cytoplasmic membrane-associated protein that indirectly coordinates endocytic membrane traffic with the actin assembly machinery |
| 9757 | **KMT2B** | Lysine Methyltransferase 2B | Histone methyltransferase. Methylates Lys-4 of histone H3. H3 Lys-4 methylation represents a specific tag for epigenetic transcriptional activation |
| 3845 | **KRAS** | KRAS Proto-Oncogene, GTPase | Ras proteins bind GDP/GTP and possess intrinsic GTPase activity. Plays an important role in the regulation of cell proliferation |
| 4216 | **MAP3K4** | Mitogen-Activated Protein Kinase Kinase Kinase 4 | Component of a protein kinase signal transduction cascade. Activates the CSBP2, P38 and JNK MAPK pathways, but not the ERK pathway. Specifically phosphorylates and activates MAP2K4 and MAP2K6 |
| 8491 | **MAP4K3** | Mitogen-Activated Protein Kinase Kinase Kinase Kinase 3 | Encodes a member of the mitogen-activated protein kinase kinase kinase kinase family. The encoded protein activates key effectors in cell signalling, among them c-Jun |
| 2122 | **MECOM** | MDS1 And EVI1 Complex Locus | Is a transcriptional regulator and oncoprotein that may be involved in hematopoiesis, apoptosis, development, and cell differentiation and proliferation |
| 9968 | **MED12** | Mediator Complex Subunit 12 | The MED12 protein is essential for activating CDK8 kinase |
| 57591 | **MKL1** | Megakaryoblastic Leukemia (Translocation) 1 | The encoded protein is predominantly nuclear and may help transduce signals from the cytoskeleton to the nucleus |
| 4292 | **MLH1** | MutL Homolog 1 | It is a human homolog of the E. coli DNA mismatch repair gene mutL, consistent with the characteristic alterations in microsatellite sequences (RER+phenotype) found in HNPCC |
| 4628 | **MYH10** | Myosin Heavy Chain 10 | Myosins are actin-dependent motor proteins with diverse functions including regulation of cytokinesis, cell motility, and cell polarity |
| 10787 | **NCKAP1** | NCK Associated Protein 1 | Among its related pathways are Immune System and E-cadherin signaling in the nascent adherens junction |
| 10397 | **NDRG1** | N-Myc Downstream Regulated 1 | The protein encoded by this gene is a cytoplasmic protein involved in stress responses, hormone responses, cell growth, and differentiation. The encoded protein is necessary for p53-mediated caspase activation and apoptosis |
| 4763 | **NF1** | Neurofibromin 1 | This gene product appears to function as a negative regulator of the ras signal transduction pathway |
| 4851 | **NOTCH1** | Notch 1 | Members of this Type I transmembrane protein family share structural characteristics including an extracellular domain consisting of multiple epidermal growth factor-like (EGF) repeats, and an intracellular domain consisting of multiple different domain types |
| 4929 | **NR4A2** | Nuclear Receptor Subfamily 4 Group A Member 2 | This gene encodes a member of the steroid-thyroid hormone-retinoid receptor superfamily. The encoded protein may act as a transcription factor |
| 4893 | **NRAS** | Neuroblastoma RAS Viral Oncogene Homolog | This shuttling is regulated through palmitoylation and depalmitoylation by the ZDHHC9-GOLGA7 complex |
| 64324 | **NSD1** | Nuclear Receptor Binding SET Domain Protein 1 | The encoded protein enhances androgen receptor (AR) transactivation, and this enhancement can be increased further in the presence of other androgen receptor associated coregulators |
| 5290 | **PIK3CA** | Phosphatidylinositol-4,5-Bisphosphate 3-Kinase Catalytic Subunit Alpha | The protein encoded by this gene represents the catalytic subunit, which uses ATP to phosphorylate PtdIns, PtdIns4P and PtdIns(4,5)P2 |
| 5431 | **POLR2B** | RNA Polymerase II Subunit B | This gene encodes the second largest subunit of RNA polymerase II (Pol II), a DNA-dependent RNA polymerase that catalyzes the transcription of DNA into precursors of mRNA, snRNA and microRNA |
| 5728 | **PTEN** | Phosphatase And Tensin Homolog | It contains a tensin like domain as well as a catalytic domain similar to that of the dual specificity protein tyrosine phosphatases |
| 5925 | **RB1** | RB Transcriptional Corepressor 1 | The protein encoded by this gene is a negative regulator of the cell cycle and was the first tumor suppressor gene found |
| 387 | **RHOA** | Ras Homolog Family Member A | Rho proteins promote reorganization of the actin cytoskeleton and regulate cell shape, attachment, and motility |
| 29072 | **SETD2** | SET Domain Containing 2 | This protein is a histone methyltransferase that is specific for lysine-36 of histone H3, and methylation of this residue is associated with active chromatin |
| 9869 | **SETDB1** | SET Domain Bifurcated 1 | This gene encodes a histone methyltransferase which regulates histone methylation, gene silencing, and transcriptional repression |
| 25942 | **SIN3A** | SIN3 Transcription Regulator Family Member A | It contains paired amphipathic helix (PAH) domains, which are important for protein-protein interactions and may mediate repression by the Mad-Max complex |
| 6654 | **SOS1** | SOS Ras/Rac Guanine Nucleotide Exchange Factor 1 | This gene encodes a protein that is a guanine nucleotide exchange factor for RAS proteins, membrane proteins that bind guanine nucleotides and participate in signal transduction pathways |
| 10274 | **STAG1** | Stromal Antigen 1 | It encodes a component of cohesin, a multisubunit protein complex that provides sister chromatid cohesion along the length of a chromosome from DNA replication through prophase and prometaphase, after which it is dissociated in preparation for segregation during anaphase |
| 10735 | **STAG2** | Stromal Antigen 2 | The protein encoded by this gene is a subunit of the cohesin complex, which regulates the separation of sister chromatids during cell division |
| 6926 | **TBX3** | T-Box 3 | T-box genes encode transcription factors involved in the regulation of developmental processes |
| 6934 | **TCF7L2** | Transcription Factor 7 Like 2 | This gene encodes a high mobility group (HMG) box-containing transcription factor that plays a key role in the Wnt signaling pathway |
| 7027 | **TFDP1** | Transcription Factor Dp-1 | The encoded protein functions as part of this complex to control the transcriptional activity of numerous genes involved in cell cycle progression from G1 to S phase |
| 7048 | **TGFBR2** | Transforming Growth Factor Beta Receptor 2 | The encoded protein is a transmembrane protein that has a protein kinase domain, forms a heterodimeric complex with another receptor protein, and binds TGF-beta |
| 7082 | **TJP1** | Tight Junction Protein 1 | This gene encodes a protein located on a cytoplasmic membrane surface of intercellular tight junctions. The encoded protein may be involved in signal transduction at cell-cell junctions |
| 10043 | **TOM1** | Target Of Myb1 Membrane Trafficking Protein | The encoded protein shares its N-terminal domain in common with proteins associated with vesicular trafficking at the endosome. It is recruited to the endosomes by its interaction with endofin |
| 7157 | **TP53** | Tumor Protein P53 | The encoded protein responds to diverse cellular stresses to regulate expression of target genes, thereby inducing cell cycle arrest, apoptosis, senescence, DNA repair, or changes in metabolism |
| 7158 | **TP53BP1** | Tumor Protein P53 Binding Protein 1 | Plays a key role in the response to DNA damage. May have a role in checkpoint signaling during mitosis. Enhances TP53-mediated transcriptional activation |
| 7204 | **TRIO** | Trio Rho Guanine Nucleotide Exchange Factor | This protein promotes the reorganization of the actin cytoskeleton, thereby playing a role in cell migration and growth |
| 4904 | **YBX1** | Y-Box Binding Protein 1 | The encoded protein functions as both a DNA and RNA binding protein and has been implicated in numerous cellular processes including regulation of transcription and translation, pre-mRNA splicing, DNA reparation and mRNA packaging |

*Built up using GeneCards®: The human gene database (Weizmann Institute of Science. v4.4.2 Build 18). Accessible through http://www.genecards.org/
