# Supplementary material for: The impact on high‐grade serous ovarian cancer of obesity and lipid metabolism‐related gene expression patterns: the underestimated driving force affecting prognosis
Source: J Cell Mol Med. 2017 Dec 20;22(3):1805–15. doi: 10.1111/jcmm.13463 (PMC5824367; doi:10.1111/jcmm.13463)
Supplement: Supplementary file 6 — Table S5 The list of driver and obesity/lipid metabolism‐related genes included in NMF clustering using RPPA data from TCGA. [file JCMM-22-1805-s006.docx]

**Supplementary Table 5 (S6):** Driver and obesity/lipid metabolism-related genes included in NMF clustering using RPPA data from TCGA

| **Driver and Obesity-related Genes used for NMF clustering analysis (RPPA mass-spectometer data from TCGA)** | | |
| --- | --- | --- |
|  |  |  |
| **Driver genes** |  | **Obesity and lipid metabolism-related genes** |
| ACO1 |  | ACACA |
| ACTG1 |  | ACAT1 |
| ADGRL2 |  | ACSL1 |
| AFF4 |  | ACSS2 |
| ARID1A |  | ADIPOQ |
| ASH1L |  | AGPAT2 |
| ASPM |  | AGRP |
| ATF1 |  | AGT |
| ATIC |  | AHSG |
| ATR |  | AQP7 |
| ATRX |  | BMP1 |
| BAP1 |  | BMP2 |
| BMPR2 |  | BMP3 |
| BRAF |  | BMP4 |
| BRCA1 |  | BSCL2 |
| BRCA2 |  | CD36 |
| CASP1 |  | CEBPA |
| CCAR1 |  | CEBPB |
| CCT5 |  | CEBPD |
| CDK12 |  | CETP |
| CHD1L |  | CFD |
| CHD4 |  | CNTFR |
| CLASP2 |  | CREB1 |
| CSDE1 |  | CYP26A1 |
| CTNNB1 |  | CYP26B1 |
| CUL2 |  | DDIT3 |
| DDX5 |  | DGAT1 |
| DLG1 |  | DLK1 |
| DNMT3A |  | DVL1 |
| EIF4A2 |  | E2F1 |
| ERBB2IP |  | E2F4 |
| FMR1 |  | EBF1 |
| GNAS |  | EGR2 |
| GOLGA5 |  | EPAS1 |
| GPS2 |  | FABP4 |
| HDAC3 |  | FGF21 |
| HSP90AA1 |  | FOXC2 |
| ITSN1 |  | FOXO1 |
| KRAS |  | GADD45A |
| MAP3K4 |  | GATA2 |
| MAP4K3 |  | GATA3 |
| MECOM |  | GATA4 |
| MED12 |  | GH1 |
| MLH1 |  | GINS3 |
| MYH10 |  | GK |
| NCKAP1 |  | HIF1A |
| NDRG1 |  | HMGA1 |
| NF1 |  | HNF1A |
| NRAS |  | ID3 |
| NSD1 |  | IGF1 |
| PIK3CA |  | IL17A |
| POLR2B |  | IL1B |
| PTEN |  | IL22 |
| RB1 |  | IL6 |
| RHOA |  | IL6R |
| SETD2 |  | IL6ST |
| SETDB1 |  | INS |
| SIN3A |  | IRS1 |
| SOS1 |  | IRS2 |
| STAG1 |  | IRS4 |
| STAG2 |  | KLF15 |
| TFDP1 |  | KLF5 |
| TJP1 |  | KLF6 |
| TOM1 |  | KLF7 |
| TP53 |  | LACTB |
| TP53BP1 |  | LEP |
| TRIO |  | LIF |
| YBX1 |  | LIFR |
|  |  | LIPE |
|  |  | LMNA |
|  |  | LPIN1 |
|  |  | LPIN2 |
|  |  | LPIN3 |
|  |  | LPL |
|  |  | MEF2A |
|  |  | MEF2B |
|  |  | MEF2C |
|  |  | MEF2D |
|  |  | MIF |
|  |  | MKKS |
|  |  | MMP9 |
|  |  | NCOA1 |
|  |  | NCOR1 |
|  |  | NCOR2 |
|  |  | NDN |
|  |  | NDRG4 |
|  |  | NLRP3 |
|  |  | NR1H3 |
|  |  | NR2F1 |
|  |  | NR3C1 |
|  |  | NRIP1 |
|  |  | OSM |
|  |  | PCK1 |
|  |  | PCK2 |
|  |  | PLIN |
|  |  | PNPLA2 |
|  |  | PNPLA3 |
|  |  | PPARD |
|  |  | PPARG |
|  |  | PPARGC1A |
|  |  | PRLR |
|  |  | PTGIS |
|  |  | PTPRE |
|  |  | RARA |
|  |  | RBL1 |
|  |  | RBL2 |
|  |  | RETN |
|  |  | RORA |
|  |  | RXRA |
|  |  | RXRG |
|  |  | SCD |
|  |  | SERPINE1 |
|  |  | SFRP4 |
|  |  | SLC12A2 |
|  |  | SLC6A5 |
|  |  | SMAD3 |
|  |  | SOCS3 |
|  |  | SP1 |
|  |  | SPP1 |
|  |  | SREBF1 |
|  |  | STAT1 |
|  |  | STAT3 |
|  |  | STAT5A |
|  |  | TGFB1 |
|  |  | TLR4 |
|  |  | TRIB3 |
|  |  | TWIST1 |
|  |  | UCP1 |
|  |  | UCP2 |
|  |  | WNT1 |
|  |  | WNT10B |
|  |  | WNT5B |
