# Supplementary material for: The impact on high‐grade serous ovarian cancer of obesity and lipid metabolism‐related gene expression patterns: the underestimated driving force affecting prognosis
Source: J Cell Mol Med. 2017 Dec 20;22(3):1805–15. doi: 10.1111/jcmm.13463 (PMC5824367; doi:10.1111/jcmm.13463)
Supplement: Supplementary file 7 — Table S6 The list of most variable genes in HGSOC according to RPPA from TCGA dataset. [file JCMM-22-1805-s007.docx]

**Supplementary Table 6 (S7):** The 208 most variable genes in HGSOC according to RPPA from TCGA dataset.

| **208 most variable proteins in High-Grade Serous Ovarian Cancer** (based on TCGA data version 2016_01_28 for OV) |
| --- |
|  |
| 14-3-3_epsilon |
| 14-3-3_zeta |
| 4E-BP1 |
| 4E-BP1_pS65 |
| 4E-BP1_pT37_T46 |
| 4E-BP1_pT70 |
| 53BP1 |
| A-Raf_pS299 |
| ACC1 |
| ACC_pS79 |
| ACVRL1 |
| ADAR1 |
| AMPK_alpha |
| AMPK_pT172 |
| AR |
| ARHI |
| ARID1A |
| ASNS |
| ATM |
| Acetyl-a-Tubulin-Lys40 |
| Akt |
| Akt_pS473 |
| Akt_pT308 |
| Annexin-1 |
| Annexin_VII |
| Axl |
| B-Raf |
| BRCA2 |
| BRD4 |
| Bad_pS112 |
| Bak |
| Bap1-c-4 |
| Bax |
| Bcl-2 |
| Bcl-xL |
| Beclin |
| Bid |
| Bim |
| C-Raf |
| C-Raf_pS338 |
| CD20 |
| CD31 |
| CD49b |
| CDK1 |
| CDK1_pY15 |
| Caspase-7_cleavedD198 |
| Caspase-8 |
| Caveolin-1 |
| Chk1 |
| Chk1_pS345 |
| Chk2 |
| Chk2_pT68 |
| Claudin-7 |
| Collagen_VI |
| Cyclin_B1 |
| Cyclin_D1 |
| Cyclin_E1 |
| Cyclin_E2 |
| DJ-1 |
| Dvl3 |
| E-Cadherin |
| EGFR |
| EGFR_pY1068 |
| EGFR_pY1173 |
| ER-alpha |
| ER-alpha_pS118 |
| ERCC1 |
| ERK2 |
| ETS-1 |
| FASN |
| FOXO3a |
| FOXO3a_pS318_S321 |
| Fibronectin |
| FoxM1 |
| G6PD |
| GAB2 |
| GAPDH |
| GATA3 |
| GATA6 |
| GSK3-alpha-beta |
| GSK3-alpha-beta_pS21_S9 |
| GSK3_pS9 |
| HER2 |
| HER2_pY1248 |
| HER3 |
| HER3_pY1289 |
| HSP70 |
| Heregulin |
| IGFBP2 |
| INPP4B |
| IRS1 |
| JAB1 |
| JNK2 |
| JNK_pT183_pY185 |
| Ku80 |
| LKB1 |
| Lck |
| MAPK_pT202_Y204 |
| MEK1 |
| MEK1_pS217_S221 |
| MIG-6 |
| MSH2 |
| MSH6 |
| MYH11 |
| Mre11 |
| Myosin-IIa |
| Myosin-IIa_pS1943 |
| N-Cadherin |
| N-Ras |
| NDRG1_pT346 |
| NF-kB-p65_pS536 |
| NF2 |
| Notch1 |
| P-Cadherin |
| PAI-1 |
| PARP1 |
| PARP_cleaved |
| PCNA |
| PDCD4 |
| PDK1 |
| PDK1_pS241 |
| PEA15 |
| PEA15_pS116 |
| PI3K-p110-alpha |
| PI3K-p85 |
| PKC-alpha |
| PKC-alpha_pS657 |
| PKC-delta_pS664 |
| PKC-pan_BetaII_pS660 |
| PR |
| PRAS40_pT246 |
| PRDX1 |
| PREX1 |
| PTEN |
| Paxillin |
| RBM15 |
| Rab11 |
| Rab25 |
| Rad50 |
| Rad51 |
| Raptor |
| Rb |
| Rb_pS807_S811 |
| Rictor |
| Rictor_pT1135 |
| S6 |
| S6_pS235_S236 |
| S6_pS240_S244 |
| SCD |
| SETD2 |
| SF2 |
| SLC1A5 |
| STAT3_pY705 |
| STAT5-alpha |
| Shc_pY317 |
| Smac |
| Smad1 |
| Smad3 |
| Smad4 |
| Snail |
| Src |
| Src_pY416 |
| Src_pY527 |
| Stathmin |
| Syk |
| TAZ |
| TFRC |
| TIGAR |
| TSC1 |
| TTF1 |
| Transglutaminase |
| Tuberin |
| Tuberin_pT1462 |
| VEGFR2 |
| VHL |
| XBP1 |
| XRCC1 |
| YAP |
| YAP_pS127 |
| YB-1 |
| YB-1_pS102 |
| alpha-Catenin |
| beta-Catenin |
| c-Jun_pS73 |
| c-Kit |
| c-Met |
| c-Met_pY1235 |
| c-Myc |
| cIAP |
| eEF2 |
| eEF2K |
| eIF4E |
| eIF4G |
| mTOR |
| mTOR_pS2448 |
| p21 |
| p27 |
| p27_pT157 |
| p27_pT198 |
| p38_MAPK |
| p38_pT180_Y182 |
| p53 |
| p62-LCK-ligand |
| p70S6K |
| p70S6K_pT389 |
| p90RSK |
| p90RSK_pT359_S363 |
|  |
| Input file for selecting top 208 genes: *.antibody_annotation.txt from RPPA_AnnotateWithGene |
| Input file for the clustering module: /xchip/cga/gdac-prod/tcga-gdac/jobResults/GDAC_TopgenesforCluster/OV-TP/22507675/OV-TP.expclu.gct |
|  |
| All data accessible at http://firebrowse.org |
