# Supplementary material for: The impact on high‐grade serous ovarian cancer of obesity and lipid metabolism‐related gene expression patterns: the underestimated driving force affecting prognosis
Source: J Cell Mol Med. 2017 Dec 20;22(3):1805–15. doi: 10.1111/jcmm.13463 (PMC5824367; doi:10.1111/jcmm.13463)
Supplement: Supplementary file 8 — Table S7 Summary of co‐occurrence and mutually exclusive analyses carried out among TP53 mutant genes and the list of obesity and lipid metabolism‐related genes significant for NMF clustering of the HGOSC cohorts. [file JCMM-22-1805-s008.docx]

**Supplementary Table 7 (S8):** Summary of co-occurrence and mutually exclusive analyses carried out among TP53 mutant gene and the list of obesity and lipid metabolism-related genes significant for NMF clustering of the HGOSC cohorts.

|  |  |  |  |  |  |  |
| --- | --- | --- | --- | --- | --- | --- |
|  | **Gene A** | **Gene B** | **p-value** | **Log Odd Ratio** | **Association** |  |
|  | **SPP1** | **AHSG** | <0.001 | >3 | Tendency towards co-occurrence |  |
|  | **PTGIS** | **CFD** | <0.001 | >3 | Tendency towards co-occurrence |  |
|  | **PTGIS** | **IGF1** | <0.001 | >3 | Tendency towards co-occurrence |  |
|  | **PTGIS** | **SFRP4** | <0.001 | >3 | Tendency towards co-occurrence |  |
|  | **PTGIS** | **BSCL2** | <0.001 | >3 | Tendency towards co-occurrence |  |
|  | **CD36** | **ACSL1** | <0.001 | >3 | Tendency towards co-occurrence |  |
|  | **CD36** | **CEBPB** | <0.001 | >3 | Tendency towards co-occurrence |  |
|  | **CD36** | **PLIN1** | <0.001 | >3 | Tendency towards co-occurrence |  |
|  | **CD36** | **LIPE** | <0.001 | >3 | Tendency towards co-occurrence |  |
|  | **CD36** | **IGF1** | <0.001 | >3 | Tendency towards co-occurrence |  |
|  | **CD36** | **PLIN2** | <0.001 | >3 | Tendency towards co-occurrence |  |
|  | **SERPINE1** | **BMP1** | <0.001 | >3 | Tendency towards co-occurrence |  |
|  | **SERPINE1** | **BSCL2** | <0.001 | >3 | Tendency towards co-occurrence |  |
|  | **TGFB1** | **RETN** | <0.001 | >3 | Tendency towards co-occurrence |  |
|  | **TGFB1** | **MMP9** | <0.001 | >3 | Tendency towards co-occurrence |  |
|  | **ACSL1** | **PLIN1** | <0.001 | >3 | Tendency towards co-occurrence |  |
|  | **ACSL1** | **LIPE** | <0.001 | >3 | Tendency towards co-occurrence |  |
|  | **AHSG** | **AGT** | <0.001 | >3 | Tendency towards co-occurrence |  |
|  | **BMP1** | **BSCL2** | <0.001 | >3 | Tendency towards co-occurrence |  |
|  | **CEBPB** | **PLIN1** | <0.001 | >3 | Tendency towards co-occurrence |  |
|  | **PLIN1** | **LIPE** | <0.001 | >3 | Tendency towards co-occurrence |  |
|  | **PLIN1** | **IGF1** | <0.001 | >3 | Tendency towards co-occurrence |  |
|  | **LIPE** | **IGF1** | <0.001 | >3 | Tendency towards co-occurrence |  |
|  | **LIPE** | **PLIN2** | <0.001 | >3 | Tendency towards co-occurrence |  |
|  | **CFD** | **RETN** | <0.001 | >3 | Tendency towards co-occurrence |  |
|  | **IGF1** | **SFRP4** | <0.001 | >3 | Tendency towards co-occurrence |  |
|  | **HLA-DRB5** | **HLA-DQB1** | <0.001 | >3 | Tendency towards co-occurrence |  |
|  | **HLA-DRB5** | **HLA-DQA1** | <0.001 | >3 | Tendency towards co-occurrence |  |
|  | **HLA-DQB1** | **HLA-DQA1** | <0.001 | >3 | Tendency towards co-occurrence |  |
|  | **NAMPT** | **MMP9** | <0.001 | >3 | Tendency towards co-occurrence |  |
|  | **SPP1** | **IRS2** | 0.001 | >3 | Tendency towards co-occurrence |  |
|  | **ACSL1** | **BSCL2** | 0.001 | >3 | Tendency towards co-occurrence |  |
|  | **LIPE** | **SCD** | 0.001 | >3 | Tendency towards co-occurrence |  |
|  | **SCD** | **IRS2** | 0.001 | >3 | Tendency towards co-occurrence |  |
|  | **CFD** | **IGF1** | 0.001 | >3 | Tendency towards co-occurrence |  |
|  | **CFD** | **SFRP4** | 0.001 | >3 | Tendency towards co-occurrence |  |
|  | **CD36** | **SCD** | 0.002 | >3 | Tendency towards co-occurrence |  |
|  | **TGFB1** | **BMP1** | 0.002 | >3 | Tendency towards co-occurrence |  |
|  | **TGFB1** | **IGF1** | 0.002 | >3 | Tendency towards co-occurrence |  |
|  | **AHSG** | **IRS2** | 0.002 | >3 | Tendency towards co-occurrence |  |
|  | **ENPP2** | **IGF1** | 0.002 | >3 | Tendency towards co-occurrence |  |
|  | **ENPP2** | **SFRP4** | 0.002 | >3 | Tendency towards co-occurrence |  |
|  | **CFD** | **BSCL2** | 0.002 | >3 | Tendency towards co-occurrence |  |
|  | **AGT** | **MMP9** | 0.002 | >3 | Tendency towards co-occurrence |  |
|  | **HMGA1** | **TP53** | 0.002 | >3 | Tendency towards co-occurrence |  |
|  | **SPP1** | **IGF1** | 0.003 | >3 | Tendency towards co-occurrence |  |
|  | **CD36** | **IRS4** | 0.003 | >3 | Tendency towards co-occurrence |  |
|  | **BMP1** | **MMP9** | 0.003 | >3 | Tendency towards co-occurrence |  |
|  | **PLIN1** | **IRS4** | 0.003 | >3 | Tendency towards co-occurrence |  |
|  | **AGT** | **RETN** | 0.003 | >3 | Tendency towards co-occurrence |  |
|  | **RETN** | **MMP9** | 0.003 | >3 | Tendency towards co-occurrence |  |
|  | **SPP1** | **SFRP4** | 0.004 | >3 | Tendency towards co-occurrence |  |
|  | **AHSG** | **IGF1** | 0.004 | >3 | Tendency towards co-occurrence |  |
|  | **BMP1** | **RETN** | 0.004 | >3 | Tendency towards co-occurrence |  |
|  | **BMP1** | **IRS4** | 0.004 | >3 | Tendency towards co-occurrence |  |
|  | **RETN** | **IRS4** | 0.004 | >3 | Tendency towards co-occurrence |  |
|  | **SERPINE1** | **SFRP4** | 0.005 | >3 | Tendency towards co-occurrence |  |
|  | **AHSG** | **SFRP4** | 0.005 | >3 | Tendency towards co-occurrence |  |
|  | **BMP1** | **IGF1** | 0.005 | >3 | Tendency towards co-occurrence |  |
|  | **CEBPB** | **NR3C1** | 0.005 | >3 | Tendency towards co-occurrence |  |
|  | **LIPE** | **BSCL2** | 0.005 | >3 | Tendency towards co-occurrence |  |
|  | **IGF1** | **RETN** | 0.005 | >3 | Tendency towards co-occurrence |  |
|  | **CD36** | **BSCL2** | 0.007 | >3 | Tendency towards co-occurrence |  |
|  | **BMP1** | **SFRP4** | 0.007 | >3 | Tendency towards co-occurrence |  |
|  | **PLIN1** | **BSCL2** | 0.007 | >3 | Tendency towards co-occurrence |  |
|  | **SFRP4** | **IRS4** | 0.007 | >3 | Tendency towards co-occurrence |  |
|  | **MMP9** | **BSCL2** | 0.007 | >3 | Tendency towards co-occurrence |  |
|  | **LACTB** | **HLA-DRB5** | 0.008 | >3 | Tendency towards co-occurrence |  |
|  | **SPP1** | **AHR** | 0.010 | >3 | Tendency towards co-occurrence |  |
|  | **ACSL1** | **PLIN2** | 0.010 | >3 | Tendency towards co-occurrence |  |
|  | **LIPE** | **NR3C1** | 0.010 | >3 | Tendency towards co-occurrence |  |
|  | **AHR** | **IRS2** | 0.010 | >3 | Tendency towards co-occurrence |  |
|  | **CD36** | **NR3C1** | 0.012 | >3 | Tendency towards co-occurrence |  |
|  | **AHSG** | **AHR** | 0.012 | >3 | Tendency towards co-occurrence |  |
|  | **AHSG** | **E2F4** | 0.012 | >3 | Tendency towards co-occurrence |  |
|  | **PLIN1** | **NR3C1** | 0.012 | >3 | Tendency towards co-occurrence |  |
|  | **IGF1** | **BSCL2** | 0.012 | 2.815 | Tendency towards co-occurrence |  |
|  | **AGT** | **E2F4** | 0.012 | >3 | Tendency towards co-occurrence |  |
|  | **CFD** | **PLIN2** | 0.013 | >3 | Tendency towards co-occurrence |  |
|  | **IGF1** | **NR3C1** | 0.015 | >3 | Tendency towards co-occurrence |  |
|  | **SFRP4** | **BSCL2** | 0.015 | 2.679 | Tendency towards co-occurrence |  |
|  | **PTGIS** | **LPIN2** | 0.017 | >3 | Tendency towards co-occurrence |  |
|  | **PTGIS** | **PLIN2** | 0.017 | >3 | Tendency towards co-occurrence |  |
|  | **TGFB1** | **PLIN2** | 0.017 | >3 | Tendency towards co-occurrence |  |
|  | **SFRP4** | **AHR** | 0.017 | >3 | Tendency towards co-occurrence |  |
|  | **ACSL1** | **CFD** | 0.020 | >3 | Tendency towards co-occurrence |  |
|  | **SCD** | **LPIN2** | 0.020 | >3 | Tendency towards co-occurrence |  |
|  | **SCD** | **PLIN2** | 0.020 | >3 | Tendency towards co-occurrence |  |
|  | **LPIN2** | **IRS2** | 0.020 | >3 | Tendency towards co-occurrence |  |
|  | **SERPINE1** | **PLIN2** | 0.023 | >3 | Tendency towards co-occurrence |  |
|  | **SERPINE1** | **FASN** | 0.023 | >3 | Tendency towards co-occurrence |  |
|  | **PLIN1** | **PLIN2** | 0.023 | >3 | Tendency towards co-occurrence |  |
|  | **PTGIS** | **ACSL1** | 0.025 | >3 | Tendency towards co-occurrence |  |
|  | **TGFB1** | **ACSL1** | 0.025 | >3 | Tendency towards co-occurrence |  |
|  | **TGFB1** | **NAMPT** | 0.025 | >3 | Tendency towards co-occurrence |  |
|  | **ENPP2** | **CEBPB** | 0.025 | >3 | Tendency towards co-occurrence |  |
|  | **ENPP2** | **NAMPT** | 0.025 | >3 | Tendency towards co-occurrence |  |
|  | **BMP1** | **PLIN2** | 0.026 | >3 | Tendency towards co-occurrence |  |
|  | **PLIN2** | **RETN** | 0.026 | >3 | Tendency towards co-occurrence |  |
|  | **PLIN2** | **IRS4** | 0.026 | >3 | Tendency towards co-occurrence |  |
|  | **ACSL1** | **SCD** | 0.030 | >3 | Tendency towards co-occurrence |  |
|  | **ACSL1** | **IRS2** | 0.030 | >3 | Tendency towards co-occurrence |  |
|  | **CEBPB** | **LIPE** | 0.030 | >3 | Tendency towards co-occurrence |  |
|  | **IGF1** | **PLIN2** | 0.030 | >3 | Tendency towards co-occurrence |  |
|  | **TGFB1** | **CFD** | 0.033 | >3 | Tendency towards co-occurrence |  |
|  | **ENPP2** | **CFD** | 0.033 | >3 | Tendency towards co-occurrence |  |
|  | **PLIN2** | **SFRP4** | 0.033 | >3 | Tendency towards co-occurrence |  |
|  | **SERPINE1** | **ACSL1** | 0.034 | >3 | Tendency towards co-occurrence |  |
|  | **ACSL1** | **MMP9** | 0.034 | >3 | Tendency towards co-occurrence |  |
|  | **AGT** | **NAMPT** | 0.034 | >3 | Tendency towards co-occurrence |  |
|  | **ACSL1** | **BMP1** | 0.039 | >3 | Tendency towards co-occurrence |  |
|  | **ACSL1** | **RETN** | 0.039 | >3 | Tendency towards co-occurrence |  |
|  | **ACSL1** | **IRS4** | 0.039 | >3 | Tendency towards co-occurrence |  |
|  | **CEBPB** | **IRS4** | 0.039 | >3 | Tendency towards co-occurrence |  |
|  | **LIPE** | **CFD** | 0.039 | >3 | Tendency towards co-occurrence |  |
|  | **PLIN2** | **BSCL2** | 0.039 | >3 | Tendency towards co-occurrence |  |
|  | **BSCL2** | **FASN** | 0.039 | >3 | Tendency towards co-occurrence |  |
|  | **PTGIS** | **TGFB1** | 0.041 | >3 | Tendency towards co-occurrence |  |
|  | **PTGIS** | **ENPP2** | 0.041 | >3 | Tendency towards co-occurrence |  |
|  | **TGFB1** | **ENPP2** | 0.041 | >3 | Tendency towards co-occurrence |  |
|  | **ACSL1** | **IGF1** | 0.044 | >3 | Tendency towards co-occurrence |  |
|  | **CEBPB** | **IGF1** | 0.044 | >3 | Tendency towards co-occurrence |  |
|  | **CD36** | **CFD** | 0.046 | >3 | Tendency towards co-occurrence |  |
|  | **SERPINE1** | **CFD** | 0.046 | >3 | Tendency towards co-occurrence |  |
|  | **AHSG** | **CFD** | 0.046 | >3 | Tendency towards co-occurrence |  |
|  | **PLIN1** | **CFD** | 0.046 | >3 | Tendency towards co-occurrence |  |
|  | **CFD** | **AGT** | 0.046 | >3 | Tendency towards co-occurrence |  |
|  | **SPP1** | **TGFB1** | 0.049 | >3 | Tendency towards co-occurrence |  |
|  | **PTGIS** | **LIPE** | 0.049 | >3 | Tendency towards co-occurrence |  |
|  | **PTGIS** | **SCD** | 0.049 | >3 | Tendency towards co-occurrence |  |
|  | **PTGIS** | **IRS2** | 0.049 | >3 | Tendency towards co-occurrence |  |
|  | **TGFB1** | **LIPE** | 0.049 | >3 | Tendency towards co-occurrence |  |
|  | **TGFB1** | **SCD** | 0.049 | >3 | Tendency towards co-occurrence |  |
|  | **ACSL1** | **SFRP4** | 0.049 | >3 | Tendency towards co-occurrence |  |
|  |  |  |  |  |  |  |
