# Supplementary material for: The impact on high‐grade serous ovarian cancer of obesity and lipid metabolism‐related gene expression patterns: the underestimated driving force affecting prognosis
Source: J Cell Mol Med. 2017 Dec 20;22(3):1805–15. doi: 10.1111/jcmm.13463 (PMC5824367; doi:10.1111/jcmm.13463)
Supplement: Supplementary file 9 [file JCMM-22-1805-s009.docx]

**Supplementary Table 1 (S1):** List of obesity and lipid metabolism related genes and its main functions

| **Gene** | **Description** | **Function** |
| --- | --- | --- |
| **ACACA** | Acetyl-CoA Carboxylase Alpha | Catalyzes the carboxylation of acetyl-CoA to malonyl-CoA, the rate-limiting step in fatty acid synthesis |
| **ACACB** | Acetyl-CoA Carboxylase Beta | Catalyzes the carboxylation of acetyl-CoA to malonyl-CoA, the rate-limiting step in fatty acid synthesis |
| **ACAT1** | Acetyl-CoA Acetyltransferase 1 | Catalyzes the reversible formation of acetoacetyl-CoA from two molecules of acetyl-CoA |
| **ACSL1** | Acyl-CoA Synthetase Long-Chain Family Member 1 | Converts free long-chain fatty acids into fatty acyl-CoA esters, and thereby play a key role in lipid biosynthesis and fatty acid degradation |
| **ACSS2** | Acyl-CoA Synthetase Short-Chain Family Member 2 | Catalyzes the activation of acetate for use in lipid synthesis and energy generation |
| **ADIPOQ** | Adiponectin, C1Q And Collagen Domain Containing | Involved with metabolic and hormonal processes |
| **AGPAT2** | 1-Acylglycerol-3-Phosphate O-Acyltransferase 2 | Converts lysophosphatidic acid to phosphatidic acid, the second step in de novo phospholipid biosynthesis |
| **AGRP** | Agouti Related Neuropeptide | Regulates hypothalamic control of feeding behavior via melanocortin receptor and/or intracellular calcium regulation, and thus plays a role in weight homeostasis |
| **AGT** | Angiotensinogen | Involved in maintaining blood pressure and in the pathogenesis of essential hypertension |
| **AHR** | Aryl Hydrocarbon Receptor | Is a ligand-activated helix-loop-helix transcription factor involved in the regulation of biological responses to planar aromatic hydrocarbons |
| **AHSG** | Alpha 2-HS Glycoprotein | Involved in several functions, such as endocytosis, brain development and the formation of bone tissue |
| **AQP7** | Aquaporin 7 | Highly expressed in the adipose tissue where the encoded protein facilitates efflux of glycerol |
| **BMP1** | Bone Morphogenetic Protein 1 | Protein that is capable of inducing formation of cartilage in vivo |
| **BMP2** | Bone Morphogenetic Protein 2 | Encodes a secreted ligand of the TGF-beta (transforming growth factor-beta) superfamily of proteins |
| **BMP3** | Bone Morphogenetic Protein 3 | Encodes a secreted ligand of the TGF-beta (transforming growth factor-beta) superfamily of proteins |
| **BMP4** | Bone Morphogenetic Protein 4 | Encodes a secreted ligand of the TGF-beta (transforming growth factor-beta) superfamily of proteins |
| **BSCL2** | BSCL2, Seipin Lipid Droplet Biogenesis Associated | This protein localizes to the endoplasmic reticulum and may be important for lipid droplet morphology |
| **CD36** | CD36 Molecule | Binds long chain fatty acids and may function in the transport and/or as a regulator of fatty acid transport |
| **CEBPA** | CCAAT/Enhancer Binding Protein Alpha | Modulates the expression of genes involved in cell cycle regulation as well as in body weight homeostasis |
| **CEBPB** | CCAAT/Enhancer Binding Protein Beta | This protein is important in the regulation of genes involved in immune and inflammatory responses |
| **CEBPD** | CCAAT/Enhancer Binding Protein Delta | This protein is important in the regulation of genes involved in immune and inflammatory responses |
| **CELF1** | CUGBP, Elav-Like Family Member 1 | Regulates pre-mRNA alternative splicing and may also be involved in mRNA editing, and translation |
| **CETP** | Cholesteryl Ester Transfer Protein | Involved in the transfer of cholesteryl ester from high density lipoprotein (HDL) to other lipoproteins |
| **CFD** | Complement Factor D | Functions as an adipokine, a cell signaling protein secreted by adipocytes, which regulates insulin secretion |
| **CNTFR** | Ciliary Neurotrophic Factor Receptor | Plays a critical role in neuronal cell survival, differentiation and gene expression |
| **CREB1** | cAMP Responsive Element Binding Protein 1 | Induces transcription of genes in response to hormonal stimulation of the cAMP pathway |
| **CYP26A1** | Cytochrome P450 Family 26 Subfamily A Member 1 | Monooxygenase which catalyzes many reactions involved in drug metabolism and synthesis of cholesterol, steroids and other lipids |
| **CYP26B1** | Cytochrome P450 Family 26 Subfamily B Member 1 | Monooxygenase which catalyzes many reactions involved in drug metabolism and synthesis of cholesterol, steroids and other lipids |
| **DDIT3** | DNA Damage Inducible Transcript 3 | Implicated in adipogenesis and erythropoiesis, is activated by endoplasmic reticulum stress, and promotes apoptosis |
| **DGAT1** | Diacylglycerol O-Acyltransferase 1 | Key metabolic enzyme, its activity may be associated with obesity and other metabolic diseases |
| **DLK1** | Delta Like Non-Canonical Notch Ligand 1 | Involved in the differentiation of several cell types including adipocytes |
| **HLA-DQA1** | Major Histocompatibility Complex, Class II, DQ Alpha 1 | Plays a central role in the immune system by presenting peptides derived from extracellular proteins |
| **HLA-DQB1** | Major Histocompatibility Complex, Class II, DQ Beta 1 | Plays a central role in the immune system by presenting peptides derived from extracellular proteins |
| **HLA-DRB5** | Major Histocompatibility Complex, Class II, DR Beta 5 | Plays a central role in the immune system by presenting peptides derived from extracellular proteins |
| **DVL1** | Dishevelled Segment Polarity Protein 1 | Regulates cell proliferation, acting as a transducer molecule for developmental processes, including segmentation and neuroblast specification |
| **E2F1** | E2F Transcription Factor 1 | Can mediate both cell proliferation and p53-dependent/independent apoptosis |
| **E2F4** | E2F Transcription Factor 4 | Plays an important role in the suppression of proliferation-associated genes, and its gene mutation and increased expression may be associated with human cancer |
| **EBF1** | Early B-Cell Factor 1 | Among its related pathways are Regulation of lipid metabolism by Peroxisome proliferator-activated receptor alpha (PPARalpha) and Developmental Biology |
| **EGR2** | Early Growth Response 2 | Is a transcription factor with three tandem C2H2-type zinc fingers |
| **ENPP2** | Ectonucleotide Pyrophosphatase/Phosphodiesterase 2 | Phosphodiesterase and a phospholipase, which catalyzes production of lysophosphatidic acid (LPA) in extracellular fluids |
| **EPAS1** | Endothelial PAS Domain Protein 1 | Transcription factor involved in the induction of genes regulated by oxygen, which is induced as oxygen levels fall |
| **FABP4** | Fatty Acid Binding Protein 4 | Cytoplasmic protein that binds long-chain fatty acids and other hydrophobic ligands, found in adipocytes |
| **FASN** | Fatty Acid Synthase | Catalyzes the synthesis of palmitate from acetyl-CoA and malonyl-CoA, in the presence of NADPH, into long-chain saturated fatty acids |
| **FGF21** | Fibroblast Growth Factor 21 | Is a secreted endocrine factor that functions as a major metabolic regulator |
| **FOXC2** | Forkhead Box C2 | It may play a role in the development of mesenchymal tissues |
| **FOXO1** | Forkhead Box O1 | It may play a role in myogenic growth and differentiation |
| **FTO** | FTO, Alpha-Ketoglutarate Dependent Dioxygenase | Function unknown, studies in mice and humans indicate a role in nervous and cardiovascular systems and a strong association with body mass index, obesity risk, and type 2 diabetes |
| **GADD45A** | Growth Arrest And DNA Damage Inducible Alpha | Is a member of a group of genes whose transcript levels are increased following stressful growth arrest conditions and treatment with DNA-damaging agents |
| **GATA2** | GATA Binding Protein 2 | Plays an essential role in regulating transcription of genes involved in the development and proliferation of hematopoietic and endocrine cell lineages |
| **GATA3** | GATA Binding Protein 3 | Is an important regulator of T-cell development and plays an important role in endothelial cell biology |
| **GATA4** | GATA Binding Protein 4 | Is thought to regulate genes involved in embryogenesis and in myocardial differentiation and function, and is necessary for normal testicular development |
| **GH1** | Growth Hormone 1 | Is a member of the somatotropin/prolactin family of hormones which play an important role in growth control |
| **GINS3** | GINS Complex Subunit 3 | Is essential for the initiation of DNA replication and replisome progression in eukaryotes |
| **GK** | Glycerol Kinase | Is a key enzyme in the regulation of glycerol uptake and metabolism |
| **HIF1A** | Hypoxia Inducible Factor 1 Alpha Subunit | Master regulator of cellular and systemic homeostatic response to hypoxia by activating transcription of many genes, including those involved in energy metabolism, angiogenesis, apoptosis, and other genes whose protein products increase oxygen delivery or facilitate metabolic adaptation to hypoxia |
| **HMGA1** | High Mobility Group AT-Hook 1 | Involved in the regulation of gene transcription, integration of retroviruses into chromosomes, and the metastatic progression of cancer cells |
| **HNF1A** | HNF1 Homeobox A | Is a transcription factor required for the expression of several liver-specific genes |
| **ID3** | Inhibitor Of DNA Binding 3, HLH Protein | Inhibits the DNA binding of any HLH protein with which it interacts |
| **IGF1** | Insulin Like Growth Factor 1 | Is similar to insulin in function and structure and is a member of a family of proteins involved in mediating growth and development |
| **IL17A** | Interleukin 17A | This cytokine can stimulate the expression of IL6 and cyclooxygenase-2 (PTGS2/COX-2), as well as enhance the production of nitric oxide (NO) |
| **IL1B** | Interleukin 1 Beta | This cytokine is an important mediator of the inflammatory response, and is involved in a variety of cellular activities, including cell proliferation, differentiation, and apoptosis |
| **IL22** | Interleukin 22 | Cytokine that contributes to the inflammatory response in vivo |
| **IL6** | Interleukin 6 | Cytokine that functions in inflammation and the maturation of B cells |
| **IL6R** | Interleukin 6 Receptor | Is a potent pleiotropic cytokine that regulates cell growth and differentiation and plays an important role in the immune response |
| **IL6ST** | Interleukin 6 Signal Transducer | Is a signal transducer shared by many cytokines, including interleukin 6 (IL6), ciliary neurotrophic factor (CNTF), leukemia inhibitory factor (LIF), and oncostatin M (OSM) |
| **INS** | Insulin | Binding of insulin to the insulin receptor (INSR) stimulates glucose uptake |
| **IRS1** | Insulin Receptor Substrate 1 | May mediate the control of various cellular processes by insulin |
| **IRS2** | Insulin Receptor Substrate 2 | A cytoplasmic signaling molecule that mediates effects of insulin, insulin-like growth factor 1, and other cytokines by acting as a molecular adaptor between diverse receptor tyrosine kinases and downstream effectors |
| **IRS4** | Insulin Receptor Substrate 4 | Has been shown to associate with cytoplasmic signalling molecules that contain SH2 domains |
| **KLF15** | Kruppel Like Factor 15 | Is a negative regulator of TP53 acetylation. Inhibits NF-kappa-B activation through repression of EP300-dependent RELA acetylation |
| **KLF5** | Kruppel Like Factor 5 | This protein acts downstream of multiple different signaling pathways and is regulated by post-translational modification. It may participate in both promoting and suppressing cell proliferation. |
| **KLF6** | Kruppel Like Factor 6 | The zinc finger protein is a transcriptional activator, and functions as a tumor suppressor |
| **KLF7** | Kruppel Like Factor 7 | This protein may contribute to the progression of type 2 diabetes by inhibiting insulin expression and secretion in pancreatic beta-cells and by deregulating adipocytokine secretion in adipocytes |
| **LACTB** | Lactamase Beta | Increased expression of the related mouse gene was found to be associated with obesity |
| **LEP** | Leptin | This protein, which acts through the leptin receptor, functions as part of a signaling pathway that can inhibit food intake and/or regulate energy expenditure to maintain constancy of the adipose mass |
| **LIF** | Leukemia Inhibitory Factor | Is a pleiotropic cytokine with roles in several different systems. It is involved in the induction of hematopoietic differentiation in normal and myeloid leukemia cells |
| **LIFR** | Leukemia Inhibitory Factor Receptor Alpha | This protein combines with a high-affinity converter subunit, gp130, to form a receptor complex that mediates the action of the leukemia inhibitory factor, a polyfunctional cytokine that is involved in cellular differentiation, proliferation and survival in the adult and the embryo |
| **LIPE** | Lipase E, Hormone Sensitive Type | The long form is expressed in steroidogenic tissues such as testis, where it converts cholesteryl esters to free cholesterol for steroid hormone production. The short form is expressed in adipose tissue, among others, where it hydrolyzes stored triglycerides to free fatty acids |
| **LMNA** | Lamin A/C | Lamin proteins are thought to be involved in nuclear stability, chromatin structure and gene expression |
| **LPIN1** | Lipin 1 | Expression of this gene is required for adipocyte differentiation and it also functions as a nuclear transcriptional coactivator with some peroxisome proliferator-activated receptors to modulate expression of other genes involved in lipid metabolism |
| **LPIN2** | Lipin 2 | This gene functions during normal adipose tissue development and may play a role in human triglyceride metabolism |
| **LPIN3** | Lipin 3 | Lipin complexes act in the cytoplasm to catalyze the dephosphorylation of phosphatidic acid to produce diacylglycerol, which is the precursor of both triglycerides and phospholipids |
| **LPL** | Lipoprotein Lipase | functions as a homodimer, and has the dual functions of triglyceride hydrolase and ligand/bridging factor for receptor-mediated lipoprotein uptake |
| **MEF2A** | Myocyte Enhancer Factor 2A | Can act as a homodimer or as a heterodimer and is involved in several cellular processes, including muscle development, neuronal differentiation, cell growth control, and apoptosis |
| **MEF2B** | Myocyte Enhancer Factor 2B | Is thought to regulate gene expression, including expression of the smooth muscle myosin heavy chain gene |
| **MEF2C** | Myocyte Enhancer Factor 2C | May play a role in maintaining the differentiated state of muscle cells |
| **MEF2D** | Myocyte Enhancer Factor 2D | Involved in control of muscle and neuronal cell differentiation and development, and are regulated by class II histone deacetylases |
| **MIF** | Macrophage Migration Inhibitory Factor (Glycosylation-Inhibiting Factor) | It plays a role in the regulation of macrophage function in host defense through the suppression of anti-inflammatory effects of glucocorticoids |
| **MKKS** | McKusick-Kaufman Syndrome | Is a centrosome-shuttling protein and plays an important role in cytokinesis |
| **MMP9** | Matrix Metallopeptidase 9 | Involved in the breakdown of extracellular matrix in normal physiological processes, such as embryonic development, reproduction, and tissue remodeling, as well as in disease processes, such as arthritis and metastasis |
| **NAMPT** | Nicotinamide Phosphoribosyltransferase | The protein belongs to the nicotinic acid phosphoribosyltransferase (NAPRTase) family and is thought to be involved in many important biological processes, including metabolism, stress response and aging |
| **NCOA1** | Nuclear Receptor Coactivator 1 | Stimulates the transcriptional activities in a hormone-dependent fashion |
| **NCOR1** | Nuclear Receptor Corepressor 1 | Mediates ligand-independent transcription repression of thyroid-hormone and retinoic-acid receptors by promoting chromatin condensation and preventing access of the transcription machinery |
| **NCOR2** | Nuclear Receptor Corepressor 2 | Nuclear receptor co-repressor that mediates transcriptional silencing of certain target genes |
| **NDN** | Necdin, MAGE Family Member | May suppress growth in postmitotic neurons |
| **NDRG4** | NDRG Family Member 4 | Is a cytoplasmic protein that is required for cell cycle progression and survival in primary astrocytes and may be involved in the regulation of mitogenic signalling in vascular smooth muscles cells |
| **NLRP3** | NLR Family Pyrin Domain Containing 3 | Functions as an upstream activator of NF-kappaB signaling, and it plays a role in the regulation of inflammation, the immune response, and apoptosis |
| **NR1H3** | Nuclear Receptor Subfamily 1 Group H Member 3 | Key regulators of macrophage function, controlling transcriptional programs involved in lipid homeostasis and inflammation |
| **NR2F1** | Nuclear Receptor Subfamily 2 Group F Member 1 | Is a nuclear hormone receptor and transcriptional regulator |
| **NR3C1** | Nuclear Receptor Subfamily 3 Group C Member 1 | It is involved in inflammatory responses, cellular proliferation, and differentiation in target tissues |
| **NRIP1** | Nuclear Receptor Interacting Protein 1 | Modulates transcriptional activity of the estrogen receptor |
| **OSM** | Oncostatin M | Is a secreted cytokine and growth regulator that inhibits the proliferation of a number of tumor cell lines |
| **PCK1** | Phosphoenolpyruvate Carboxykinase 1 | This gene can be regulated by insulin, glucocorticoids, glucagon, cAMP, and diet |
| **PCK2** | Phosphoenolpyruvate Carboxykinase 2, Mitochondrial | Enzyme that catalyzes the conversion of oxaloacetate to phosphoenolpyruvate in the presence of guanosine triphosphate (GTP) |
| **PLIN1** | Perilipin 1 | Coats lipid storage droplets in adipocytes, thereby protecting them until they can be broken down by hormone-sensitive lipase |
| **PLIN2** | Perilipin 2 | Is associated with the lipid globule surface membrane material, and maybe involved in development and maintenance of adipose tissue |
| **PNPLA2** | Patatin Like Phospholipase Domain Containing 2 | Catalyzes the first step in the hydrolysis of triglycerides in adipose tissue |
| **PNPLA3** | Patatin Like Phospholipase Domain Containing 3 | Is a triacylglycerol lipase that mediates triacylglycerol hydrolysis in adipocytes |
| **PPARA** | Peroxisome Proliferator Activated Receptor Alpha | PPARs affect the expression of target genes involved in cell proliferation, cell differentiation and in immune and inflammation responses |
| **PPARD** | Peroxisome Proliferator Activated Receptor Delta | PPARs mediate a variety of biological processes, and may be involved in the development of several chronic diseases, including diabetes, obesity, atherosclerosis, and cancer |
| **PPARG** | Peroxisome Proliferator Activated Receptor Gamma | Is a regulator of adipocyte differentiation |
| **PPARGC1A** | PPARG Coactivator 1 Alpha | Is a transcriptional coactivator that regulates the genes involved in energy metabolism |
| **PRLR** | Prolactin Receptor | Receptor for the anterior pituitary hormone, prolactin, may function to modulate the endocrine and autocrine effects of prolactin in normal tissue and cancer |
| **PTGIS** | Prostaglandin I2 Synthase | The cytochrome P450 proteins are monooxygenases which catalyze many reactions involved in drug metabolism and synthesis of cholesterol, steroids and other lipids |
| **PTPRE** | Protein Tyrosine Phosphatase, Receptor Type E | PTPs are known to be signaling molecules that regulate a variety of cellular processes including cell growth, differentiation, mitotic cycle, and oncogenic transformation |
| **RARA** | Retinoic Acid Receptor Alpha | This gene has been implicated in regulation of development, differentiation, apoptosis, granulopoeisis, and transcription of clock genes |
| **RBL1** | RB Transcriptional Corepressor Like 1 | Due to the sequence and biochemical similarities with the RB1 protein, it is thought that the protein encoded by this gene may also be a tumor suppressor |
| **RBL2** | RB Transcriptional Corepressor Like 2 | Key regulator of entry into cell division |
| **RETN** | Resistin | Is secreted by adipocytes, and may be the hormone potentially linking obesity to type II diabetes |
| **RORA** | RAR Related Orphan Receptor A | Is a member of the NR1 subfamily of nuclear hormone receptors |
| **RXRA** | Retinoid X Receptor Alpha | Nuclear receptor that mediates the biological effects of retinoids by their involvement in retinoic acid-mediated gene activation |
| **RXRG** | Retinoid X Receptor Gamma | Nuclear receptor which is involved in mediating the antiproliferative effects of retinoic acid (RA) |
| **SCD** | Stearoyl-CoA Desaturase | Enzyme involved in fatty acid biosynthesis, primarily the synthesis of oleic acid |
| **SERPINE1** | Serpin Family E Member 1 | Is the principal inhibitor of tissue plasminogen activator (tPA) and urokinase (uPA), and hence is an inhibitor of fibrinolysis |
| **SFRP4** | Secreted Frizzled Related Protein 4 | Acts as soluble modulators of Wnt signaling |
| **SLC2A2** | Solute Carrier Family 2 Member 2 | Mediates facilitated bidirectional glucose transport. Because of its low affinity for glucose, it has been suggested as a glucose sensor |
| **SLC6A5** | Solute Carrier Family 6 Member 5 | Is responsible for the clearance of extracellular glycine during glycine-mediated neurotransmission |
| **SMAD3** | SMAD Family Member 3 | This protein functions as a transcriptional modulator activated by transforming growth factor-beta and is thought to play a role in the regulation of carcinogenesis |
| **SOCS3** | Suppressor Of Cytokine Signaling 3 | SSI family members are cytokine-inducible negative regulators of cytokine signaling |
| **SP1** | Sp1 Transcription Factor | Involved in many cellular processes, including cell differentiation, cell growth, apoptosis, immune responses, response to DNA damage, and chromatin remodeling |
| **SPP1** | Secreted Phosphoprotein 1 | Is involved in the attachment of osteoclasts to the mineralized bone matrix |
| **SREBF1** | Sterol Regulatory Element Binding Transcription Factor 1 | Transcription factor that binds to the sterol regulatory element-1 (SRE1), which is a decamer flanking the low density lipoprotein receptor gene and some genes involved in sterol biosynthesis |
| **STAT1** | Signal Transducer And Activator Of Transcription 1 | This protein mediates the expression of a variety of genes, which is thought to be important for cell viability in response to different cell stimuli and pathogens |
| **STAT3** | Signal Transducer And Activator Of Transcription 3 | This protein mediates the expression of a variety of genes in response to cell stimuli, and thus plays a key role in many cellular processes such as cell growth and apoptosis |
| **STAT5A** | Signal Transducer And Activator Of Transcription 5A | Activation of this protein in myeloma and lymphoma associated with a TEL/JAK2 gene fusion is independent of cell stimulus and has been shown to be essential for tumorigenesis |
| **TGFB1** | Transforming Growth Factor Beta 1 | Regulates cell proliferation, differentiation and growth, and can modulate expression and activation of other growth factors including interferon gamma and tumor necrosis factor alpha |
| **TLR4** | Toll Like Receptor 4 | Plays a fundamental role in pathogen recognition and activation of innate immunity |
| **TRIB3** | Tribbles Pseudokinase 3 | Is a negative regulator of NF-kappaB and can also sensitize cells to TNF- and TRAIL-induced apoptosis |
| **TWIST1** | Twist Family BHLH Transcription Factor 1 | Basic helix-loop-helix (bHLH) transcription factors have been implicated in cell lineage determination and differentiation |
| **UCP1** | Uncoupling Protein 1 | UCPs separate oxidative phosphorylation from ATP synthesis with energy dissipated as heat, also referred to as the mitochondrial proton leak |
| **UCP2** | Uncoupling Protein 2 | UCPs facilitate the transfer of anions from the inner to the outer mitochondrial membrane and the return transfer of protons from the outer to the inner mitochondrial membrane |
| **WNT1** | Wnt Family Member 1 | Implicated in oncogenesis and in several developmental processes, including regulation of cell fate and patterning during embryogenesis |
| **WNT10B** | Wnt Family Member 10B | It may be involved in breast cancer, and its protein signaling is likely a molecular switch that governs adipogenesis |
| **WNT5B** | Wnt Family Member 5B | Implicated in oncogenesis and in several developmental processes, including regulation of cell fate and patterning during embryogenesis |

*Built up using GeneCards®: The human gene database (Weizmann Institute of Science. v4.4.2 Build 18). Accessible through <http://www.genecards.org/>

**Supplementary Table 2 (S2):** Significant Obesity and lipid metabolism-related genes for US-OVCA Cohort NMF Clustering (RNA microarray data)

| **Rank** | **Feature** | **Description** | **Upregulated in Cluster [C]** | **Feature P** | **FDR(BH)** | **Q Value** |
| --- | --- | --- | --- | --- | --- | --- |
| 25 | 100271849 | MEF2B | **G1** | 0,00020 | 0,00062 | 0,00011 |
| 26 | 55902 | ACSS2 | **G1** | 0,00020 | 0,00062 | 0,00011 |
| 31 | 6927 | HNF1A | **G1** | 0,00040 | 0,00109 | 0,00011 |
| 33 | 7350 | UCP1 | **G1** | 0,00040 | 0,00109 | 0,00011 |
| 36 | 5618 | PRLR | **G1** | 0,00040 | 0,00109 | 0,00011 |
| 41 | 6258 | RXRG | **G1** | 0,00040 | 0,00109 | 0,00011 |
| 42 | 8195 | MKKS | **G1** | 0,00040 | 0,00109 | 0,00011 |
| 45 | 3159 | HMGA1 | **G1** | 0,00140 | 0,00314 | 0,00030 |
| 49 | 5106 | PCK2 | **G1** | 0,00180 | 0,00391 | 0,00037 |
| 51 | 1855 | DVL1 | **G1** | 0,00080 | 0,00199 | 0,00019 |
| 53 | 57761 | TRIB3 | **G1** | 0,00180 | 0,00391 | 0,00037 |
| 54 | 1869 | E2F1 | **G1** | 0,00100 | 0,00236 | 0,00022 |
| 55 | 3570 | IL6R | **G1** | 0,00080 | 0,00199 | 0,00019 |
| 58 | 5933 | RBL1 | **G1** | 0,00140 | 0,00314 | 0,00030 |
| 61 | 64900 | LPIN3 | **G1** | 0,00260 | 0,00531 | 0,00050 |
| 62 | 2688 | GH1 | **G1** | 0,00300 | 0,00587 | 0,00055 |
| 65 | 2626 | GATA4 | **G1** | 0,00260 | 0,00531 | 0,00050 |
| 66 | 2624 | GATA2 | **G1** | 0,00280 | 0,00556 | 0,00052 |
| 67 | 3977 | LIFR | **G1** | 0,00380 | 0,00723 | 0,00068 |
| 68 | 197 | AHSG | **G1** | 0,00600 | 0,01097 | 0,00103 |
| 69 | 3605 | IL17A | **G1** | 0,00480 | 0,00901 | 0,00085 |
| 73 | 1271 | CNTFR | **G1** | 0,00780 | 0,01408 | 0,00133 |
| 77 | 8204 | NRIP1 | **G1** | 0,01000 | 0,01737 | 0,00164 |
| 16 | 1649 | DDIT3 | **G1** | 0,00020 | 0,00062 | 0,00011 |
| 85 | 2908 | NR3C1 | **G2** | 0,00940 | 0,01654 | 0,00156 |
| 82 | 181 | AGRP | **G2** | 0,00800 | 0,01425 | 0,00134 |
| 79 | 56603 | CYP26B1 | **G2** | 0,00580 | 0,01075 | 0,00101 |
| 78 | 688 | KLF5 | **G2** | 0,00220 | 0,00470 | 0,00044 |
| 76 | 8609 | KLF7 | **G2** | 0,00380 | 0,00723 | 0,00068 |
| 74 | 6774 | STAT3 | **G2** | 0,00140 | 0,00314 | 0,00030 |
| 72 | 4205 | MEF2A | **G2** | 0,00260 | 0,00531 | 0,00050 |
| 71 | 2308 | FOXO1 | **G2** | 0,00100 | 0,00236 | 0,00022 |
| 70 | 8694 | DGAT1 | **G2** | 0,00280 | 0,00556 | 0,00052 |
| 64 | 4000 | LMNA | **G2** | 0,00060 | 0,00154 | 0,00015 |
| 63 | 5468 | PPARG | **G2** | 0,00040 | 0,00109 | 0,00011 |
| 60 | 8660 | IRS2 | **G2** | 0,00060 | 0,00154 | 0,00015 |
| 59 | 4023 | LPL | **G2** | 0,00060 | 0,00154 | 0,00015 |
| 57 | 3991 | LIPE | **G2** | 0,00020 | 0,00062 | 0,00011 |
| 56 | 10135 | NAMPT | **G2** | 0,00100 | 0,00236 | 0,00022 |
| 52 | 3399 | ID3 | **G2** | 0,00020 | 0,00062 | 0,00011 |
| 50 | 9612 | NCOR2 | **G2** | 0,00020 | 0,00062 | 0,00011 |
| 48 | 1647 | GADD45A | **G2** | 0,00020 | 0,00062 | 0,00011 |
| 47 | 364 | AQP7 | **G2** | 0,00020 | 0,00062 | 0,00011 |
| 46 | 650 | BMP2 | **G2** | 0,00020 | 0,00062 | 0,00011 |
| 44 | 3572 | IL6ST | **G2** | 0,00020 | 0,00062 | 0,00011 |
| 43 | 1051 | CEBPB | **G2** | 0,00020 | 0,00062 | 0,00011 |
| 40 | 3976 | LIF | **G2** | 0,00020 | 0,00062 | 0,00011 |
| 39 | 7040 | TGFB1 | **G2** | 0,00020 | 0,00062 | 0,00011 |
| 38 | 4318 | MMP9 | **G2** | 0,00020 | 0,00062 | 0,00011 |
| 37 | 2034 | EPAS1 | **G2** | 0,00020 | 0,00062 | 0,00011 |
| 35 | 1071 | CETP | **G2** | 0,00020 | 0,00062 | 0,00011 |
| 34 | 114294 | LACTB | **G2** | 0,00020 | 0,00062 | 0,00011 |
| 32 | 7291 | TWIST1 | **G2** | 0,00020 | 0,00062 | 0,00011 |
| 30 | 652 | BMP4 | **G2** | 0,00020 | 0,00062 | 0,00011 |
| 29 | 948 | CD36 | **G2** | 0,00020 | 0,00062 | 0,00011 |
| 28 | 649 | BMP1 | **G2** | 0,00020 | 0,00062 | 0,00011 |
| 27 | 1675 | CFD | **G2** | 0,00020 | 0,00062 | 0,00011 |
| 24 | 3952 | LEP | **G2** | 0,00020 | 0,00062 | 0,00011 |
| 23 | 9021 | SOCS3 | **G2** | 0,00020 | 0,00062 | 0,00011 |
| 22 | 56729 | RETN | **G2** | 0,00020 | 0,00062 | 0,00011 |
| 21 | 5791 | PTPRE | **G2** | 0,00020 | 0,00062 | 0,00011 |
| 20 | 2180 | ACSL1 | **G2** | 0,00020 | 0,00062 | 0,00011 |
| 19 | 1879 | EBF1 | **G2** | 0,00020 | 0,00062 | 0,00011 |
| 18 | 3553 | IL1B | **G2** | 0,00020 | 0,00062 | 0,00011 |
| 17 | 3479 | IGF1 | **G2** | 0,00020 | 0,00062 | 0,00011 |
| 15 | 1592 | CYP26A1 | **G2** | 0,00020 | 0,00062 | 0,00011 |
| 14 | 6696 | SPP1 | **G2** | 0,00020 | 0,00062 | 0,00011 |
| 13 | 4208 | MEF2C | **G2** | 0,00020 | 0,00062 | 0,00011 |
| 12 | 7099 | TLR4 | **G2** | 0,00020 | 0,00062 | 0,00011 |
| 11 | 5346 | PLIN1 | **G2** | 0,00020 | 0,00062 | 0,00011 |
| 10 | 5008 | OSM | **G2** | 0,00020 | 0,00062 | 0,00011 |
| 9 | 1316 | KLF6 | **G2** | 0,00020 | 0,00062 | 0,00011 |
| 8 | 1959 | EGR2 | **G2** | 0,00020 | 0,00062 | 0,00011 |
| 7 | 3569 | IL6 | **G2** | 0,00020 | 0,00062 | 0,00011 |
| 6 | 114548 | NLRP3 | **G2** | 0,00020 | 0,00062 | 0,00011 |
| 5 | 5054 | SERPINE1 | **G2** | 0,00020 | 0,00062 | 0,00011 |
| 4 | 9370 | ADIPOQ | **G2** | 0,00020 | 0,00062 | 0,00011 |
| 3 | 2167 | FABP4 | **G2** | 0,00020 | 0,00062 | 0,00011 |
| 2 | 6424 | SFRP4 | **G2** | 0,00020 | 0,00062 | 0,00011 |
| 1 | 5740 | PTGIS | **G2** | 0,00020 | 0,00062 | 0,00011 |

**Supplementary Table 3 (S3):** Statistical analysis of differences in distribution of robust RNAseq, copy number alteration, methylation and RPPA subgroups between the two clusters obtained by NMF analysis using obesity and lipid metabolism related gene expression.

|  |  |  |  |  |  |  |  |
| --- | --- | --- | --- | --- | --- | --- | --- |
|  |  |  | **NMF clustering by obesity/metabolism-related genes** | | |  |  |
|  |  |  | **cluster 1** |  | **cluster 2** | **p-value** |  |
|  |  |  |  |  |  |  |  |
|  | **RNAseq clustering** | |  |  |  |  |  |
|  | n |  | 116 |  | 170 |  |  |
|  |  | I | 38 (32.8%) |  | 102 (60%) | <0.0001 |  |
|  |  | II | 7 (6%) |  | 44 (25.9%) |  |  |
|  |  | III | 71 (61.2%) |  | 24 (14.1%) |  |  |
|  |  |  |  |  |  |  |  |
|  | **CNA clustering** | |  |  |  |  |  |
|  | n |  | 178 |  | 342 |  |  |
|  |  | I | 60 (33.7%) |  | 158 (46.2%) | 0.002 |  |
|  |  | II | 55 (30.9%) |  | 62 (18.1%) |  |  |
|  |  | III | 63 (35.4%) |  | 122 (35.7%) |  |  |
|  |  |  |  |  |  |  |  |
|  | **Methylation clustering** | |  |  |  |  |  |
|  | n |  | 186 |  | 350 |  |  |
|  |  | I | 53 (28.5%) |  | 58 (16.6%) | 0.0001 |  |
|  |  | II | 32 (17.2%) |  | 57 (16.3%) |  |  |
|  |  | III | 77 (41.4%) |  | 100 (28.6%) |  |  |
|  |  | IV | 24 (12.9%) |  | 135 (38.6%) |  |  |
|  |  |  |  |  |  |  |  |
|  | **RPPA clustering** | |  |  |  |  |  |
|  | n |  | 138 |  | 261 |  |  |
|  |  | I | 78 (56.5%) |  | 63 (24.1%) | <0.0001 |  |
|  |  | II | 20 (14.5%) |  | 34 (13%) |  |  |
|  |  | III | 14 (10.1%) |  | 43 (16.5%) |  |  |
|  |  | IV | 26 (18.8%) |  | 121 (46.4%) |  |  |
|  |  |  |  |  |  |  |  |

**Supplementary Table 4 (S4):** List of cancer driver genes and its functions

| **GENE_ID** | **Gene** | **Description** | **Function** |
| --- | --- | --- | --- |
| 48 | **ACO1** | Aconitase 1 | Functions as an essential enzyme in the TCA cycle and interacts with mRNA to control the levels of iron inside cells |
| 71 | **ACTG1** | Actin Gamma 1 | Involved in various types of cell motility, and maintenance of the cytoskeleton |
| 23266 | **ADGRL2** | Adhesion G Protein-Coupled Receptor L2 | Participates in the regulation of exocytosis |
| 27125 | **AFF4** | AF4/FMR2 Family Member 4 | Belongs to the AF4 family of transcription factors involved in leukemia |
| 139285 | **AMER1** | APC Membrane Recruitment Protein 1 | His gene upregulates trancriptional activation by the Wilms tumor protein and interacts with many other proteins, including CTNNB1, APC, AXIN1, and AXIN2 |
| 8289 | **ARID1A** | AT-Rich Interaction Domain 1A | Thought to regulate transcription of certain genes by altering the chromatin structure around those genes |
| 55870 | **ASH1L** | ASH1 Like Histone Lysine Methyltransferase | A member of the trithorax group of transcriptional activators |
| 259266 | **ASPM** | Abnormal Spindle Microtubule Assembly | Essential for normal mitotic spindle function in embryonic neuroblasts |
| 466 | **ATF1** | Activating Transcription Factor 1 | It influences cellular physiologic processes by regulating the expression of downstream target genes, which are related to growth, survival, and other cellular activities |
| 471 | **ATIC** | 5-Aminoimidazole-4-Carboxamide Ribonucleotide Formyltransferase/IMP Cyclohydrolase | Catalyzes the last two steps of the de novo purine biosynthetic pathway |
| 545 | **ATR** | ATR Serine/Threonine Kinase | This kinase has been shown to phosphorylate checkpoint kinase CHK1, checkpoint proteins RAD17, and RAD9, as well as tumor suppressor protein BRCA1 |
| 546 | **ATRX** | ATRX, Chromatin Remodeler | This protein is found to undergo cell cycle-dependent phosphorylation, which regulates its nuclear matrix and chromatin association, and suggests its involvement in the gene regulation at interphase and chromosomal segregation in mitosis |
| 8314 | **BAP1** | BRCA1 Associated Protein 1 | Binds to the breast cancer type 1 susceptibility protein (BRCA1) via the RING finger domain of the latter and acts as a tumor suppressor |
| 29994 | **BAZ2B** | Bromodomain Adjacent To Zinc Finger Domain 2B | Members of this gene family encode proteins that are integral components of chromatin remodeling complexes |
| 659 | **BMPR2** | Bone Morphogenetic Protein Receptor Type 2 | Encodes a member of the bone morphogenetic protein (BMP) receptor family of transmembrane serine/threonine kinases |
| 673 | **BRAF** | B-Raf Proto-Oncogene, Serine/Threonine Kinase | Plays a role in regulating the MAP kinase/ERKs signaling pathway, which affects cell division, differentiation, and secretion |
| 672 | **BRCA1** | BRCA1, DNA Repair Associated | Plays a role in maintaining genomic stability, and it also acts as a tumor suppressor |
| 675 | **BRCA2** | BRCA2, DNA Repair Associated | Involved in maintenance of genome stability, specifically the homologous recombination pathway for double-strand DNA repair |
| 834 | **CASP1** | Caspase 1 | Plays a central role in the execution-phase of cell apoptosis |
| 55749 | **CCAR1** | Cell Division Cycle And Apoptosis Regulator 1 | Associates with components of the Mediator and p160 coactivator complexes that play a role as intermediaries transducing regulatory signals from upstream transcriptional activator proteins to basal transcription machinery at the core promoter |
| 22948 | **CCT5** | Chaperonin Containing TCP1 Subunit 5 | Is a molecular chaperone that is a member of the chaperonin containing TCP1 complex (CCT), also known as the TCP1 ring complex (TRiC) |
| 51755 | **CDK12** | Cyclin Dependent Kinase 12 | Regulates the expression of genes involved in DNA repair and is required for the maintenance of genomic stability |
| 9557 | **CHD1L** | Chromodomain Helicase DNA Binding Protein 1 Like | DNA helicase protein involved in DNA repair |
| 1108 | **CHD4** | Chromodomain Helicase DNA Binding Protein 4 | Plays an important role in epigenetic transcriptional repression |
| 23122 | **CLASP2** | Cytoplasmic Linker Associated Protein 2 | Involved in the nucleation of noncentrosomal microtubules originating from the trans-Golgi network (TGN). Required for the polarization of the cytoplasmic microtubule arrays in migrating cells towards the leading edge of the cell |
| 63967 | **CLSPN** | Claspin | Is an essential upstream regulator of checkpoint kinase 1 and triggers a checkpoint arrest of the cell cycle in response to replicative stress or DNA damage |
| 7812 | **CSDE1** | Cold Shock Domain Containing E1 | Involved in translationally coupled mRNA turnover |
| 1499 | **CTNNB1** | Catenin Beta 1 | Is part of a complex of proteins that constitute adherens junctions (AJs). AJs are necessary for the creation and maintenance of epithelial cell layers by regulating cell growth and adhesion between cells |
| 8453 | **CUL2** | Cullin 2 | Core component of multiple cullin-RING-based ECS (ElonginB/C-CUL2/5-SOCS-box protein) E3 ubiquitin-protein ligase complexes, which mediate the ubiquitination of target proteins |
| 1655 | **DDX5** | DEAD-Box Helicase 5 | Implicated in a number of cellular processes involving alteration of RNA secondary structure, such as translation initiation, nuclear and mitochondrial splicing, and ribosome and spliceosome assembly |
| 1739 | **DLG1** | Discs Large MAGUK Scaffold Protein 1 | May have a role in septate junction formation, signal transduction, cell proliferation, synaptogenesis and lymphocyte activation |
| 1788 | **DNMT3A** | DNA Methyltransferase 3 Alpha | Encodes a DNA methyltransferase that is thought to function in de novo methylation, rather than maintenance methylation |
| 9451 | **EIF2AK3** | Eukaryotic Translation Initiation Factor 2 Alpha Kinase 3 | Phosphorylates the alpha subunit of eukaryotic translation-initiation factor 2, leading to its inactivation, and thus to a rapid reduction of translational initiation and repression of global protein synthesis |
| 1974 | **EIF4A2** | Eukaryotic Translation Initiation Factor 4A2 | ATP-dependent RNA helicase which is a subunit of the eIF4F complex involved in cap recognition and is required for mRNA binding to ribosome |
| 55914 | **ERBB2IP** | Erbb2 Interacting Protein | It binds to the unphosphorylated form of the ERBB2 protein and regulates ERBB2 function and localization. It has also been shown to affect the Ras signaling pathway by disrupting Ras-Raf interaction |
| 2157 | **F8** | Coagulation Factor VIII | Participates in the intrinsic pathway of blood coagulation; factor VIII is a cofactor for factor IXa which, in the presence of Ca+2 and phospholipids, converts factor X to the activated form Xa |
| 55294 | **FBXW7** | F-Box And WD Repeat Domain Containing 7 | Constitutes one of the four subunits of ubiquitin protein ligase complex called SCFs (SKP1-cullin-F-box), which function in phosphorylation-dependent ubiquitination |
| 2322 | **FLT3** | Fms Related Tyrosine Kinase 3 | A class III receptor tyrosine kinase that regulates hematopoiesis. The activated receptor kinase subsequently phosphorylates and activates multiple cytoplasmic effector molecules in pathways involved in apoptosis, proliferation, and differentiation of hematopoietic cells in bone marrow |
| 2332 | **FMR1** | Fragile X Mental Retardation 1 | Involved in mRNA trafficking from the nucleus to the cytoplasm |
| 2778 | **GNAS** | GNAS Complex Locus | Guanine nucleotide-binding proteins (G proteins) function as transducers in numerous signaling pathways controlled by G protein-coupled receptors (GPCRs) |
| 9950 | **GOLGA5** | Golgin A5 | This golgin is a coiled-coil membrane protein that has been postulated to play a role in vesicle tethering and docking |
| 2874 | **GPS2** | G Protein Pathway Suppressor 2 | Encodes a protein involved in G protein-mitogen-activated protein kinase (MAPK) signaling cascades |
| 8841 | **HDAC3** | Histone Deacetylase 3 | Plays a critical role in transcriptional regulation, cell cycle progression, and developmental events. Histone acetylation/deacetylation alters chromosome structure and affects transcription factor access to DNA |
| 3082 | **HGF** | Hepatocyte Growth Factor | Binds to the hepatocyte growth factor receptor to regulate cell growth, cell motility and morphogenesis in numerous cell and tissue types |
| 3320 | **HSP90AA1** | Heat Shock Protein 90 Alpha Family Class A Member 1 | Aids in the proper folding of specific target proteins by use of an ATPase activity that is modulated by co-chaperones |
| 6453 | **ITSN1** | Intersectin 1 | Is a cytoplasmic membrane-associated protein that indirectly coordinates endocytic membrane traffic with the actin assembly machinery |
| 9757 | **KMT2B** | Lysine Methyltransferase 2B | Histone methyltransferase. Methylates Lys-4 of histone H3. H3 Lys-4 methylation represents a specific tag for epigenetic transcriptional activation |
| 3845 | **KRAS** | KRAS Proto-Oncogene, GTPase | Ras proteins bind GDP/GTP and possess intrinsic GTPase activity. Plays an important role in the regulation of cell proliferation |
| 4216 | **MAP3K4** | Mitogen-Activated Protein Kinase Kinase Kinase 4 | Component of a protein kinase signal transduction cascade. Activates the CSBP2, P38 and JNK MAPK pathways, but not the ERK pathway. Specifically phosphorylates and activates MAP2K4 and MAP2K6 |
| 8491 | **MAP4K3** | Mitogen-Activated Protein Kinase Kinase Kinase Kinase 3 | Encodes a member of the mitogen-activated protein kinase kinase kinase kinase family. The encoded protein activates key effectors in cell signalling, among them c-Jun |
| 2122 | **MECOM** | MDS1 And EVI1 Complex Locus | Is a transcriptional regulator and oncoprotein that may be involved in hematopoiesis, apoptosis, development, and cell differentiation and proliferation |
| 9968 | **MED12** | Mediator Complex Subunit 12 | The MED12 protein is essential for activating CDK8 kinase |
| 57591 | **MKL1** | Megakaryoblastic Leukemia (Translocation) 1 | The encoded protein is predominantly nuclear and may help transduce signals from the cytoskeleton to the nucleus |
| 4292 | **MLH1** | MutL Homolog 1 | It is a human homolog of the E. coli DNA mismatch repair gene mutL, consistent with the characteristic alterations in microsatellite sequences (RER+phenotype) found in HNPCC |
| 4628 | **MYH10** | Myosin Heavy Chain 10 | Myosins are actin-dependent motor proteins with diverse functions including regulation of cytokinesis, cell motility, and cell polarity |
| 10787 | **NCKAP1** | NCK Associated Protein 1 | Among its related pathways are Immune System and E-cadherin signaling in the nascent adherens junction |
| 10397 | **NDRG1** | N-Myc Downstream Regulated 1 | The protein encoded by this gene is a cytoplasmic protein involved in stress responses, hormone responses, cell growth, and differentiation. The encoded protein is necessary for p53-mediated caspase activation and apoptosis |
| 4763 | **NF1** | Neurofibromin 1 | This gene product appears to function as a negative regulator of the ras signal transduction pathway |
| 4851 | **NOTCH1** | Notch 1 | Members of this Type I transmembrane protein family share structural characteristics including an extracellular domain consisting of multiple epidermal growth factor-like (EGF) repeats, and an intracellular domain consisting of multiple different domain types |
| 4929 | **NR4A2** | Nuclear Receptor Subfamily 4 Group A Member 2 | This gene encodes a member of the steroid-thyroid hormone-retinoid receptor superfamily. The encoded protein may act as a transcription factor |
| 4893 | **NRAS** | Neuroblastoma RAS Viral Oncogene Homolog | This shuttling is regulated through palmitoylation and depalmitoylation by the ZDHHC9-GOLGA7 complex |
| 64324 | **NSD1** | Nuclear Receptor Binding SET Domain Protein 1 | The encoded protein enhances androgen receptor (AR) transactivation, and this enhancement can be increased further in the presence of other androgen receptor associated coregulators |
| 5290 | **PIK3CA** | Phosphatidylinositol-4,5-Bisphosphate 3-Kinase Catalytic Subunit Alpha | The protein encoded by this gene represents the catalytic subunit, which uses ATP to phosphorylate PtdIns, PtdIns4P and PtdIns(4,5)P2 |
| 5431 | **POLR2B** | RNA Polymerase II Subunit B | This gene encodes the second largest subunit of RNA polymerase II (Pol II), a DNA-dependent RNA polymerase that catalyzes the transcription of DNA into precursors of mRNA, snRNA and microRNA |
| 5728 | **PTEN** | Phosphatase And Tensin Homolog | It contains a tensin like domain as well as a catalytic domain similar to that of the dual specificity protein tyrosine phosphatases |
| 5925 | **RB1** | RB Transcriptional Corepressor 1 | The protein encoded by this gene is a negative regulator of the cell cycle and was the first tumor suppressor gene found |
| 387 | **RHOA** | Ras Homolog Family Member A | Rho proteins promote reorganization of the actin cytoskeleton and regulate cell shape, attachment, and motility |
| 29072 | **SETD2** | SET Domain Containing 2 | This protein is a histone methyltransferase that is specific for lysine-36 of histone H3, and methylation of this residue is associated with active chromatin |
| 9869 | **SETDB1** | SET Domain Bifurcated 1 | This gene encodes a histone methyltransferase which regulates histone methylation, gene silencing, and transcriptional repression |
| 25942 | **SIN3A** | SIN3 Transcription Regulator Family Member A | It contains paired amphipathic helix (PAH) domains, which are important for protein-protein interactions and may mediate repression by the Mad-Max complex |
| 6654 | **SOS1** | SOS Ras/Rac Guanine Nucleotide Exchange Factor 1 | This gene encodes a protein that is a guanine nucleotide exchange factor for RAS proteins, membrane proteins that bind guanine nucleotides and participate in signal transduction pathways |
| 10274 | **STAG1** | Stromal Antigen 1 | It encodes a component of cohesin, a multisubunit protein complex that provides sister chromatid cohesion along the length of a chromosome from DNA replication through prophase and prometaphase, after which it is dissociated in preparation for segregation during anaphase |
| 10735 | **STAG2** | Stromal Antigen 2 | The protein encoded by this gene is a subunit of the cohesin complex, which regulates the separation of sister chromatids during cell division |
| 6926 | **TBX3** | T-Box 3 | T-box genes encode transcription factors involved in the regulation of developmental processes |
| 6934 | **TCF7L2** | Transcription Factor 7 Like 2 | This gene encodes a high mobility group (HMG) box-containing transcription factor that plays a key role in the Wnt signaling pathway |
| 7027 | **TFDP1** | Transcription Factor Dp-1 | The encoded protein functions as part of this complex to control the transcriptional activity of numerous genes involved in cell cycle progression from G1 to S phase |
| 7048 | **TGFBR2** | Transforming Growth Factor Beta Receptor 2 | The encoded protein is a transmembrane protein that has a protein kinase domain, forms a heterodimeric complex with another receptor protein, and binds TGF-beta |
| 7082 | **TJP1** | Tight Junction Protein 1 | This gene encodes a protein located on a cytoplasmic membrane surface of intercellular tight junctions. The encoded protein may be involved in signal transduction at cell-cell junctions |
| 10043 | **TOM1** | Target Of Myb1 Membrane Trafficking Protein | The encoded protein shares its N-terminal domain in common with proteins associated with vesicular trafficking at the endosome. It is recruited to the endosomes by its interaction with endofin |
| 7157 | **TP53** | Tumor Protein P53 | The encoded protein responds to diverse cellular stresses to regulate expression of target genes, thereby inducing cell cycle arrest, apoptosis, senescence, DNA repair, or changes in metabolism |
| 7158 | **TP53BP1** | Tumor Protein P53 Binding Protein 1 | Plays a key role in the response to DNA damage. May have a role in checkpoint signaling during mitosis. Enhances TP53-mediated transcriptional activation |
| 7204 | **TRIO** | Trio Rho Guanine Nucleotide Exchange Factor | This protein promotes the reorganization of the actin cytoskeleton, thereby playing a role in cell migration and growth |
| 4904 | **YBX1** | Y-Box Binding Protein 1 | The encoded protein functions as both a DNA and RNA binding protein and has been implicated in numerous cellular processes including regulation of transcription and translation, pre-mRNA splicing, DNA reparation and mRNA packaging |

*Built up using GeneCards®: The human gene database (Weizmann Institute of Science. v4.4.2 Build 18). Accessible through http://www.genecards.org/

**Supplementary Table 5 (S6):** Driver and obesity/lipid metabolism-related genes included in NMF clustering using RPPA data from TCGA

| **Driver and Obesity-related Genes used for NMF clustering analysis (RPPA mass-spectometer data from TCGA)** | | |
| --- | --- | --- |
|  |  |  |
| **Driver genes** |  | **Obesity and lipid metabolism-related genes** |
| ACO1 |  | ACACA |
| ACTG1 |  | ACAT1 |
| ADGRL2 |  | ACSL1 |
| AFF4 |  | ACSS2 |
| ARID1A |  | ADIPOQ |
| ASH1L |  | AGPAT2 |
| ASPM |  | AGRP |
| ATF1 |  | AGT |
| ATIC |  | AHSG |
| ATR |  | AQP7 |
| ATRX |  | BMP1 |
| BAP1 |  | BMP2 |
| BMPR2 |  | BMP3 |
| BRAF |  | BMP4 |
| BRCA1 |  | BSCL2 |
| BRCA2 |  | CD36 |
| CASP1 |  | CEBPA |
| CCAR1 |  | CEBPB |
| CCT5 |  | CEBPD |
| CDK12 |  | CETP |
| CHD1L |  | CFD |
| CHD4 |  | CNTFR |
| CLASP2 |  | CREB1 |
| CSDE1 |  | CYP26A1 |
| CTNNB1 |  | CYP26B1 |
| CUL2 |  | DDIT3 |
| DDX5 |  | DGAT1 |
| DLG1 |  | DLK1 |
| DNMT3A |  | DVL1 |
| EIF4A2 |  | E2F1 |
| ERBB2IP |  | E2F4 |
| FMR1 |  | EBF1 |
| GNAS |  | EGR2 |
| GOLGA5 |  | EPAS1 |
| GPS2 |  | FABP4 |
| HDAC3 |  | FGF21 |
| HSP90AA1 |  | FOXC2 |
| ITSN1 |  | FOXO1 |
| KRAS |  | GADD45A |
| MAP3K4 |  | GATA2 |
| MAP4K3 |  | GATA3 |
| MECOM |  | GATA4 |
| MED12 |  | GH1 |
| MLH1 |  | GINS3 |
| MYH10 |  | GK |
| NCKAP1 |  | HIF1A |
| NDRG1 |  | HMGA1 |
| NF1 |  | HNF1A |
| NRAS |  | ID3 |
| NSD1 |  | IGF1 |
| PIK3CA |  | IL17A |
| POLR2B |  | IL1B |
| PTEN |  | IL22 |
| RB1 |  | IL6 |
| RHOA |  | IL6R |
| SETD2 |  | IL6ST |
| SETDB1 |  | INS |
| SIN3A |  | IRS1 |
| SOS1 |  | IRS2 |
| STAG1 |  | IRS4 |
| STAG2 |  | KLF15 |
| TFDP1 |  | KLF5 |
| TJP1 |  | KLF6 |
| TOM1 |  | KLF7 |
| TP53 |  | LACTB |
| TP53BP1 |  | LEP |
| TRIO |  | LIF |
| YBX1 |  | LIFR |
|  |  | LIPE |
|  |  | LMNA |
|  |  | LPIN1 |
|  |  | LPIN2 |
|  |  | LPIN3 |
|  |  | LPL |
|  |  | MEF2A |
|  |  | MEF2B |
|  |  | MEF2C |
|  |  | MEF2D |
|  |  | MIF |
|  |  | MKKS |
|  |  | MMP9 |
|  |  | NCOA1 |
|  |  | NCOR1 |
|  |  | NCOR2 |
|  |  | NDN |
|  |  | NDRG4 |
|  |  | NLRP3 |
|  |  | NR1H3 |
|  |  | NR2F1 |
|  |  | NR3C1 |
|  |  | NRIP1 |
|  |  | OSM |
|  |  | PCK1 |
|  |  | PCK2 |
|  |  | PLIN |
|  |  | PNPLA2 |
|  |  | PNPLA3 |
|  |  | PPARD |
|  |  | PPARG |
|  |  | PPARGC1A |
|  |  | PRLR |
|  |  | PTGIS |
|  |  | PTPRE |
|  |  | RARA |
|  |  | RBL1 |
|  |  | RBL2 |
|  |  | RETN |
|  |  | RORA |
|  |  | RXRA |
|  |  | RXRG |
|  |  | SCD |
|  |  | SERPINE1 |
|  |  | SFRP4 |
|  |  | SLC12A2 |
|  |  | SLC6A5 |
|  |  | SMAD3 |
|  |  | SOCS3 |
|  |  | SP1 |
|  |  | SPP1 |
|  |  | SREBF1 |
|  |  | STAT1 |
|  |  | STAT3 |
|  |  | STAT5A |
|  |  | TGFB1 |
|  |  | TLR4 |
|  |  | TRIB3 |
|  |  | TWIST1 |
|  |  | UCP1 |
|  |  | UCP2 |
|  |  | WNT1 |
|  |  | WNT10B |
|  |  | WNT5B |

**Supplementary Table 6 (S7):** The 208 most variable genes in HGSOC according to RPPA from TCGA dataset.

| **208 most variable proteins in High-Grade Serous Ovarian Cancer** (based on TCGA data version 2016_01_28 for OV) |
| --- |
|  |
| 14-3-3_epsilon |
| 14-3-3_zeta |
| 4E-BP1 |
| 4E-BP1_pS65 |
| 4E-BP1_pT37_T46 |
| 4E-BP1_pT70 |
| 53BP1 |
| A-Raf_pS299 |
| ACC1 |
| ACC_pS79 |
| ACVRL1 |
| ADAR1 |
| AMPK_alpha |
| AMPK_pT172 |
| AR |
| ARHI |
| ARID1A |
| ASNS |
| ATM |
| Acetyl-a-Tubulin-Lys40 |
| Akt |
| Akt_pS473 |
| Akt_pT308 |
| Annexin-1 |
| Annexin_VII |
| Axl |
| B-Raf |
| BRCA2 |
| BRD4 |
| Bad_pS112 |
| Bak |
| Bap1-c-4 |
| Bax |
| Bcl-2 |
| Bcl-xL |
| Beclin |
| Bid |
| Bim |
| C-Raf |
| C-Raf_pS338 |
| CD20 |
| CD31 |
| CD49b |
| CDK1 |
| CDK1_pY15 |
| Caspase-7_cleavedD198 |
| Caspase-8 |
| Caveolin-1 |
| Chk1 |
| Chk1_pS345 |
| Chk2 |
| Chk2_pT68 |
| Claudin-7 |
| Collagen_VI |
| Cyclin_B1 |
| Cyclin_D1 |
| Cyclin_E1 |
| Cyclin_E2 |
| DJ-1 |
| Dvl3 |
| E-Cadherin |
| EGFR |
| EGFR_pY1068 |
| EGFR_pY1173 |
| ER-alpha |
| ER-alpha_pS118 |
| ERCC1 |
| ERK2 |
| ETS-1 |
| FASN |
| FOXO3a |
| FOXO3a_pS318_S321 |
| Fibronectin |
| FoxM1 |
| G6PD |
| GAB2 |
| GAPDH |
| GATA3 |
| GATA6 |
| GSK3-alpha-beta |
| GSK3-alpha-beta_pS21_S9 |
| GSK3_pS9 |
| HER2 |
| HER2_pY1248 |
| HER3 |
| HER3_pY1289 |
| HSP70 |
| Heregulin |
| IGFBP2 |
| INPP4B |
| IRS1 |
| JAB1 |
| JNK2 |
| JNK_pT183_pY185 |
| Ku80 |
| LKB1 |
| Lck |
| MAPK_pT202_Y204 |
| MEK1 |
| MEK1_pS217_S221 |
| MIG-6 |
| MSH2 |
| MSH6 |
| MYH11 |
| Mre11 |
| Myosin-IIa |
| Myosin-IIa_pS1943 |
| N-Cadherin |
| N-Ras |
| NDRG1_pT346 |
| NF-kB-p65_pS536 |
| NF2 |
| Notch1 |
| P-Cadherin |
| PAI-1 |
| PARP1 |
| PARP_cleaved |
| PCNA |
| PDCD4 |
| PDK1 |
| PDK1_pS241 |
| PEA15 |
| PEA15_pS116 |
| PI3K-p110-alpha |
| PI3K-p85 |
| PKC-alpha |
| PKC-alpha_pS657 |
| PKC-delta_pS664 |
| PKC-pan_BetaII_pS660 |
| PR |
| PRAS40_pT246 |
| PRDX1 |
| PREX1 |
| PTEN |
| Paxillin |
| RBM15 |
| Rab11 |
| Rab25 |
| Rad50 |
| Rad51 |
| Raptor |
| Rb |
| Rb_pS807_S811 |
| Rictor |
| Rictor_pT1135 |
| S6 |
| S6_pS235_S236 |
| S6_pS240_S244 |
| SCD |
| SETD2 |
| SF2 |
| SLC1A5 |
| STAT3_pY705 |
| STAT5-alpha |
| Shc_pY317 |
| Smac |
| Smad1 |
| Smad3 |
| Smad4 |
| Snail |
| Src |
| Src_pY416 |
| Src_pY527 |
| Stathmin |
| Syk |
| TAZ |
| TFRC |
| TIGAR |
| TSC1 |
| TTF1 |
| Transglutaminase |
| Tuberin |
| Tuberin_pT1462 |
| VEGFR2 |
| VHL |
| XBP1 |
| XRCC1 |
| YAP |
| YAP_pS127 |
| YB-1 |
| YB-1_pS102 |
| alpha-Catenin |
| beta-Catenin |
| c-Jun_pS73 |
| c-Kit |
| c-Met |
| c-Met_pY1235 |
| c-Myc |
| cIAP |
| eEF2 |
| eEF2K |
| eIF4E |
| eIF4G |
| mTOR |
| mTOR_pS2448 |
| p21 |
| p27 |
| p27_pT157 |
| p27_pT198 |
| p38_MAPK |
| p38_pT180_Y182 |
| p53 |
| p62-LCK-ligand |
| p70S6K |
| p70S6K_pT389 |
| p90RSK |
| p90RSK_pT359_S363 |
|  |
| Input file for selecting top 208 genes: *.antibody_annotation.txt from RPPA_AnnotateWithGene |
| Input file for the clustering module: /xchip/cga/gdac-prod/tcga-gdac/jobResults/GDAC_TopgenesforCluster/OV-TP/22507675/OV-TP.expclu.gct |
|  |
| All data accessible at http://firebrowse.org |

**Supplementary Table 7 (S8):** Summary of co-occurrence and mutually exclusive analyses carried out among TP53 mutant gene and the list of obesity and lipid metabolism-related genes significant for NMF clustering of the HGOSC cohorts.

|  |  |  |  |  |  |  |
| --- | --- | --- | --- | --- | --- | --- |
|  | **Gene A** | **Gene B** | **p-value** | **Log Odd Ratio** | **Association** |  |
|  | **SPP1** | **AHSG** | <0.001 | >3 | Tendency towards co-occurrence |  |
|  | **PTGIS** | **CFD** | <0.001 | >3 | Tendency towards co-occurrence |  |
|  | **PTGIS** | **IGF1** | <0.001 | >3 | Tendency towards co-occurrence |  |
|  | **PTGIS** | **SFRP4** | <0.001 | >3 | Tendency towards co-occurrence |  |
|  | **PTGIS** | **BSCL2** | <0.001 | >3 | Tendency towards co-occurrence |  |
|  | **CD36** | **ACSL1** | <0.001 | >3 | Tendency towards co-occurrence |  |
|  | **CD36** | **CEBPB** | <0.001 | >3 | Tendency towards co-occurrence |  |
|  | **CD36** | **PLIN1** | <0.001 | >3 | Tendency towards co-occurrence |  |
|  | **CD36** | **LIPE** | <0.001 | >3 | Tendency towards co-occurrence |  |
|  | **CD36** | **IGF1** | <0.001 | >3 | Tendency towards co-occurrence |  |
|  | **CD36** | **PLIN2** | <0.001 | >3 | Tendency towards co-occurrence |  |
|  | **SERPINE1** | **BMP1** | <0.001 | >3 | Tendency towards co-occurrence |  |
|  | **SERPINE1** | **BSCL2** | <0.001 | >3 | Tendency towards co-occurrence |  |
|  | **TGFB1** | **RETN** | <0.001 | >3 | Tendency towards co-occurrence |  |
|  | **TGFB1** | **MMP9** | <0.001 | >3 | Tendency towards co-occurrence |  |
|  | **ACSL1** | **PLIN1** | <0.001 | >3 | Tendency towards co-occurrence |  |
|  | **ACSL1** | **LIPE** | <0.001 | >3 | Tendency towards co-occurrence |  |
|  | **AHSG** | **AGT** | <0.001 | >3 | Tendency towards co-occurrence |  |
|  | **BMP1** | **BSCL2** | <0.001 | >3 | Tendency towards co-occurrence |  |
|  | **CEBPB** | **PLIN1** | <0.001 | >3 | Tendency towards co-occurrence |  |
|  | **PLIN1** | **LIPE** | <0.001 | >3 | Tendency towards co-occurrence |  |
|  | **PLIN1** | **IGF1** | <0.001 | >3 | Tendency towards co-occurrence |  |
|  | **LIPE** | **IGF1** | <0.001 | >3 | Tendency towards co-occurrence |  |
|  | **LIPE** | **PLIN2** | <0.001 | >3 | Tendency towards co-occurrence |  |
|  | **CFD** | **RETN** | <0.001 | >3 | Tendency towards co-occurrence |  |
|  | **IGF1** | **SFRP4** | <0.001 | >3 | Tendency towards co-occurrence |  |
|  | **HLA-DRB5** | **HLA-DQB1** | <0.001 | >3 | Tendency towards co-occurrence |  |
|  | **HLA-DRB5** | **HLA-DQA1** | <0.001 | >3 | Tendency towards co-occurrence |  |
|  | **HLA-DQB1** | **HLA-DQA1** | <0.001 | >3 | Tendency towards co-occurrence |  |
|  | **NAMPT** | **MMP9** | <0.001 | >3 | Tendency towards co-occurrence |  |
|  | **SPP1** | **IRS2** | 0.001 | >3 | Tendency towards co-occurrence |  |
|  | **ACSL1** | **BSCL2** | 0.001 | >3 | Tendency towards co-occurrence |  |
|  | **LIPE** | **SCD** | 0.001 | >3 | Tendency towards co-occurrence |  |
|  | **SCD** | **IRS2** | 0.001 | >3 | Tendency towards co-occurrence |  |
|  | **CFD** | **IGF1** | 0.001 | >3 | Tendency towards co-occurrence |  |
|  | **CFD** | **SFRP4** | 0.001 | >3 | Tendency towards co-occurrence |  |
|  | **CD36** | **SCD** | 0.002 | >3 | Tendency towards co-occurrence |  |
|  | **TGFB1** | **BMP1** | 0.002 | >3 | Tendency towards co-occurrence |  |
|  | **TGFB1** | **IGF1** | 0.002 | >3 | Tendency towards co-occurrence |  |
|  | **AHSG** | **IRS2** | 0.002 | >3 | Tendency towards co-occurrence |  |
|  | **ENPP2** | **IGF1** | 0.002 | >3 | Tendency towards co-occurrence |  |
|  | **ENPP2** | **SFRP4** | 0.002 | >3 | Tendency towards co-occurrence |  |
|  | **CFD** | **BSCL2** | 0.002 | >3 | Tendency towards co-occurrence |  |
|  | **AGT** | **MMP9** | 0.002 | >3 | Tendency towards co-occurrence |  |
|  | **HMGA1** | **TP53** | 0.002 | >3 | Tendency towards co-occurrence |  |
|  | **SPP1** | **IGF1** | 0.003 | >3 | Tendency towards co-occurrence |  |
|  | **CD36** | **IRS4** | 0.003 | >3 | Tendency towards co-occurrence |  |
|  | **BMP1** | **MMP9** | 0.003 | >3 | Tendency towards co-occurrence |  |
|  | **PLIN1** | **IRS4** | 0.003 | >3 | Tendency towards co-occurrence |  |
|  | **AGT** | **RETN** | 0.003 | >3 | Tendency towards co-occurrence |  |
|  | **RETN** | **MMP9** | 0.003 | >3 | Tendency towards co-occurrence |  |
|  | **SPP1** | **SFRP4** | 0.004 | >3 | Tendency towards co-occurrence |  |
|  | **AHSG** | **IGF1** | 0.004 | >3 | Tendency towards co-occurrence |  |
|  | **BMP1** | **RETN** | 0.004 | >3 | Tendency towards co-occurrence |  |
|  | **BMP1** | **IRS4** | 0.004 | >3 | Tendency towards co-occurrence |  |
|  | **RETN** | **IRS4** | 0.004 | >3 | Tendency towards co-occurrence |  |
|  | **SERPINE1** | **SFRP4** | 0.005 | >3 | Tendency towards co-occurrence |  |
|  | **AHSG** | **SFRP4** | 0.005 | >3 | Tendency towards co-occurrence |  |
|  | **BMP1** | **IGF1** | 0.005 | >3 | Tendency towards co-occurrence |  |
|  | **CEBPB** | **NR3C1** | 0.005 | >3 | Tendency towards co-occurrence |  |
|  | **LIPE** | **BSCL2** | 0.005 | >3 | Tendency towards co-occurrence |  |
|  | **IGF1** | **RETN** | 0.005 | >3 | Tendency towards co-occurrence |  |
|  | **CD36** | **BSCL2** | 0.007 | >3 | Tendency towards co-occurrence |  |
|  | **BMP1** | **SFRP4** | 0.007 | >3 | Tendency towards co-occurrence |  |
|  | **PLIN1** | **BSCL2** | 0.007 | >3 | Tendency towards co-occurrence |  |
|  | **SFRP4** | **IRS4** | 0.007 | >3 | Tendency towards co-occurrence |  |
|  | **MMP9** | **BSCL2** | 0.007 | >3 | Tendency towards co-occurrence |  |
|  | **LACTB** | **HLA-DRB5** | 0.008 | >3 | Tendency towards co-occurrence |  |
|  | **SPP1** | **AHR** | 0.010 | >3 | Tendency towards co-occurrence |  |
|  | **ACSL1** | **PLIN2** | 0.010 | >3 | Tendency towards co-occurrence |  |
|  | **LIPE** | **NR3C1** | 0.010 | >3 | Tendency towards co-occurrence |  |
|  | **AHR** | **IRS2** | 0.010 | >3 | Tendency towards co-occurrence |  |
|  | **CD36** | **NR3C1** | 0.012 | >3 | Tendency towards co-occurrence |  |
|  | **AHSG** | **AHR** | 0.012 | >3 | Tendency towards co-occurrence |  |
|  | **AHSG** | **E2F4** | 0.012 | >3 | Tendency towards co-occurrence |  |
|  | **PLIN1** | **NR3C1** | 0.012 | >3 | Tendency towards co-occurrence |  |
|  | **IGF1** | **BSCL2** | 0.012 | 2.815 | Tendency towards co-occurrence |  |
|  | **AGT** | **E2F4** | 0.012 | >3 | Tendency towards co-occurrence |  |
|  | **CFD** | **PLIN2** | 0.013 | >3 | Tendency towards co-occurrence |  |
|  | **IGF1** | **NR3C1** | 0.015 | >3 | Tendency towards co-occurrence |  |
|  | **SFRP4** | **BSCL2** | 0.015 | 2.679 | Tendency towards co-occurrence |  |
|  | **PTGIS** | **LPIN2** | 0.017 | >3 | Tendency towards co-occurrence |  |
|  | **PTGIS** | **PLIN2** | 0.017 | >3 | Tendency towards co-occurrence |  |
|  | **TGFB1** | **PLIN2** | 0.017 | >3 | Tendency towards co-occurrence |  |
|  | **SFRP4** | **AHR** | 0.017 | >3 | Tendency towards co-occurrence |  |
|  | **ACSL1** | **CFD** | 0.020 | >3 | Tendency towards co-occurrence |  |
|  | **SCD** | **LPIN2** | 0.020 | >3 | Tendency towards co-occurrence |  |
|  | **SCD** | **PLIN2** | 0.020 | >3 | Tendency towards co-occurrence |  |
|  | **LPIN2** | **IRS2** | 0.020 | >3 | Tendency towards co-occurrence |  |
|  | **SERPINE1** | **PLIN2** | 0.023 | >3 | Tendency towards co-occurrence |  |
|  | **SERPINE1** | **FASN** | 0.023 | >3 | Tendency towards co-occurrence |  |
|  | **PLIN1** | **PLIN2** | 0.023 | >3 | Tendency towards co-occurrence |  |
|  | **PTGIS** | **ACSL1** | 0.025 | >3 | Tendency towards co-occurrence |  |
|  | **TGFB1** | **ACSL1** | 0.025 | >3 | Tendency towards co-occurrence |  |
|  | **TGFB1** | **NAMPT** | 0.025 | >3 | Tendency towards co-occurrence |  |
|  | **ENPP2** | **CEBPB** | 0.025 | >3 | Tendency towards co-occurrence |  |
|  | **ENPP2** | **NAMPT** | 0.025 | >3 | Tendency towards co-occurrence |  |
|  | **BMP1** | **PLIN2** | 0.026 | >3 | Tendency towards co-occurrence |  |
|  | **PLIN2** | **RETN** | 0.026 | >3 | Tendency towards co-occurrence |  |
|  | **PLIN2** | **IRS4** | 0.026 | >3 | Tendency towards co-occurrence |  |
|  | **ACSL1** | **SCD** | 0.030 | >3 | Tendency towards co-occurrence |  |
|  | **ACSL1** | **IRS2** | 0.030 | >3 | Tendency towards co-occurrence |  |
|  | **CEBPB** | **LIPE** | 0.030 | >3 | Tendency towards co-occurrence |  |
|  | **IGF1** | **PLIN2** | 0.030 | >3 | Tendency towards co-occurrence |  |
|  | **TGFB1** | **CFD** | 0.033 | >3 | Tendency towards co-occurrence |  |
|  | **ENPP2** | **CFD** | 0.033 | >3 | Tendency towards co-occurrence |  |
|  | **PLIN2** | **SFRP4** | 0.033 | >3 | Tendency towards co-occurrence |  |
|  | **SERPINE1** | **ACSL1** | 0.034 | >3 | Tendency towards co-occurrence |  |
|  | **ACSL1** | **MMP9** | 0.034 | >3 | Tendency towards co-occurrence |  |
|  | **AGT** | **NAMPT** | 0.034 | >3 | Tendency towards co-occurrence |  |
|  | **ACSL1** | **BMP1** | 0.039 | >3 | Tendency towards co-occurrence |  |
|  | **ACSL1** | **RETN** | 0.039 | >3 | Tendency towards co-occurrence |  |
|  | **ACSL1** | **IRS4** | 0.039 | >3 | Tendency towards co-occurrence |  |
|  | **CEBPB** | **IRS4** | 0.039 | >3 | Tendency towards co-occurrence |  |
|  | **LIPE** | **CFD** | 0.039 | >3 | Tendency towards co-occurrence |  |
|  | **PLIN2** | **BSCL2** | 0.039 | >3 | Tendency towards co-occurrence |  |
|  | **BSCL2** | **FASN** | 0.039 | >3 | Tendency towards co-occurrence |  |
|  | **PTGIS** | **TGFB1** | 0.041 | >3 | Tendency towards co-occurrence |  |
|  | **PTGIS** | **ENPP2** | 0.041 | >3 | Tendency towards co-occurrence |  |
|  | **TGFB1** | **ENPP2** | 0.041 | >3 | Tendency towards co-occurrence |  |
|  | **ACSL1** | **IGF1** | 0.044 | >3 | Tendency towards co-occurrence |  |
|  | **CEBPB** | **IGF1** | 0.044 | >3 | Tendency towards co-occurrence |  |
|  | **CD36** | **CFD** | 0.046 | >3 | Tendency towards co-occurrence |  |
|  | **SERPINE1** | **CFD** | 0.046 | >3 | Tendency towards co-occurrence |  |
|  | **AHSG** | **CFD** | 0.046 | >3 | Tendency towards co-occurrence |  |
|  | **PLIN1** | **CFD** | 0.046 | >3 | Tendency towards co-occurrence |  |
|  | **CFD** | **AGT** | 0.046 | >3 | Tendency towards co-occurrence |  |
|  | **SPP1** | **TGFB1** | 0.049 | >3 | Tendency towards co-occurrence |  |
|  | **PTGIS** | **LIPE** | 0.049 | >3 | Tendency towards co-occurrence |  |
|  | **PTGIS** | **SCD** | 0.049 | >3 | Tendency towards co-occurrence |  |
|  | **PTGIS** | **IRS2** | 0.049 | >3 | Tendency towards co-occurrence |  |
|  | **TGFB1** | **LIPE** | 0.049 | >3 | Tendency towards co-occurrence |  |
|  | **TGFB1** | **SCD** | 0.049 | >3 | Tendency towards co-occurrence |  |
|  | **ACSL1** | **SFRP4** | 0.049 | >3 | Tendency towards co-occurrence |  |
|  |  |  |  |  |  |  |
